# Stage-specific effects of early-life tobacco exposure on mental health, inflammation, and brain structure-function coupling

**DOI:** 10.1038/s41398-026-04182-4

**Published:** 2026-07-10

**Authors:** Yanqing Zhang, Junhui Zhang, Yuanyuan Chen, Jianjun Zhu

**Affiliations:** 1 Center for Early Environment and Brain Development, School of Education, Guangzhou University, Guangzhou, China; 2 Department of Psychology, Guangzhou University, Guangzhou, China; 3 Department of Special Education, Guangzhou University, Guangzhou, China

**Keywords:** Prognostic markers, Human behaviour

## Abstract

Early-life tobacco exposure is a significant risk factor for depression and anxiety. Understanding its immune and neural mechanisms—and how risks differ by age of smoking initiation—is essential for designing effective prevention and intervention strategies. Using data from 375,903 participants in the UK Biobank, we found that in utero tobacco exposure was associated with increased risk of depression and anxiety, elevated inflammatory markers, and reduced structure-function (SF) coupling in the thalamus and putamen. Childhood smoking initiation was associated with a significantly higher risk of depression, anxiety, and systemic inflammation compared to adulthood initiation, whereas adolescent initiation did not differ significantly from adulthood initiation. Both childhood and adolescent initiation were associated with reduced SF coupling in brain regions involved in reward processing and cognitive control, relative to never-smokers. Mediation analyses revealed that systemic inflammation and SF coupling jointly mediated the association between early-life tobacco exposure and psychiatric risk. CRP emerged as a key mediator linking early smoking initiation to SF coupling alterations. Moreover, interaction models revealed that in utero tobacco exposure exacerbated the pro-inflammatory effects of childhood smoking initiation. Together, these findings underscore that early-life tobacco exposure contributes to long-term mental health vulnerability via neuroimmune pathways, with childhood representing a particularly sensitive period—underscoring the need for developmental-stage prevention and mechanism-based interventions.

## Key points

- Early-life tobacco exposure has been linked to increased risk of depression and anxiety, yet its underlying neuroimmune mechanisms remain unclear.
- In utero tobacco exposure was associated with heightened psychiatric risk, elevated inflammation, and reduced SF coupling in the thalamus and putamen.
- Childhood initiation conferred the highest postnatal risk, including increased inflammation and disrupted SF coupling in reward and control-related brain regions.
- In utero tobacco exposure exacerbated the pro-inflammatory effects of childhood smoking initiation.
- Findings highlight a neuroimmune pathway linking early tobacco exposure to long-term mental health vulnerability, with childhood as a critical period for intervention.

## Introduction

Depression and anxiety are highly prevalent psychiatric disorders [1, 2]. Early-life tobacco exposure—whether occurring in utero or through smoking initiation during childhood or adolescence—has been linked to elevated risk for these disorders in early developmental stages [3–5]. However, it remains unclear whether such exposure confers enduring mental health risks into midlife or older adulthood, and whether the timing of smoking initiation (i.e., childhood vs. adolescence) differentially influences these long-term outcomes. The biological mechanisms underlying such long-term vulnerability, particularly involving immune and neural pathways, are also poorly understood. Clarifying these mechanisms is essential for advancing our understanding of the lifespan risk architecture for psychiatric disorders and for informing mechanism-based prevention strategies.

In utero tobacco exposure has been linked to elevated levels of C-reactive protein (CRP) and interleukin-8 in neonates and infants [6, 7]; a critical next step is to determine whether these inflammatory effects persist into mid-to-late adulthood. While evidence suggests that longer smoking duration may be associated with increased systemic inflammation [8], little is known about how the timing of postnatal smoking initiation affects inflammatory profiles [9, 10]. Notably, prenatal insults may disrupt developmental trajectories [11] and heighten susceptibility to subsequent exposures [12], underscoring the need to examine whether in utero tobacco exposure interacts with the age of smoking initiation to shape inflammatory outcomes.

Peripheral cytokines can cross the blood–brain barrier and influence brain development and maintenance [13, 14], raising the possibility that neuroimmune pathways may link early tobacco exposure to long-term brain alterations. In utero tobacco exposure has been shown to be associated with reduced total brain, gray matter, and white matter volumes in children aged 6–11 years [15, 16]. Regional abnormalities have also been documented, including smaller frontal lobes in preterm infants [17], hippocampal atrophy in adults [18], and diminished ventral striatum activation during reward processing in adolescents [19]. Furthermore, earlier smoking initiation among youth correlates with reduced total grey matter volume (GMV), particularly in the frontal and temporal lobes [18, 20], and with an increase in ventral striatal GMV [18]. Structure-function (SF) coupling — a key indicator of the brain’s integrative capacity [21]— declines with age [22] and is implicated in affective disorders [23]. However, whether early-life tobacco exposure disrupts SF coupling remains unknown. Investigating SF coupling in mid-to-late adulthood provides a unique opportunity to determine whether early tobacco exposure is associated with accelerated age-related decline in integrative brain organization.

To address these gaps, we leveraged data from the UK Biobank. We first examined whether in utero tobacco exposure and early smoking initiation predicted the onset of depression and anxiety in mid- to late adulthood using Cox proportional hazards models. We then evaluated their associations with peripheral inflammation and regional SF coupling, and tested whether these neurobiological markers mediated the relationship between early-life tobacco exposure and psychiatric outcomes. Finally, we examined whether in utero tobacco exposure moderated the impact of smoking initiation age on mental health and biological indicators. Elucidating these long-term effects is critical for identifying at-risk populations and informing developmentally targeted prevention strategies.

## Methods

### Participants

The UK Biobank is a large prospective cohort comprising approximately 460,000 participants aged 37–73 years at baseline. The data received approval from the National Information Governance Board for Health and Social Care and the North West Multi-Centre Research Ethics Committee (21/NW/0157). All participants provided electronic informed consent. The current study used data accessed under Application Number 138512. Between 2006 and 2010, participants were recruited from 22 assessment centers, where they completed touchscreen questionnaires, participated in nurse-led interviews, underwent standardized physical measures, and contributed biological samples. Beginning in 2014, a subset of participants meeting specific criteria was selected for follow-up imaging assessments. Participants were excluded if key data were missing.

### Assessment of early-life tobacco exposure

Early-life tobacco exposure comprised two variables. First, in utero tobacco exposure among non-adopted participants was assessed using a self-reported questionnaire item: “Did your mother smoke regularly around the time when you were born?” Maternal smoking during the perinatal period (Data-Field 1787) was determined as present or absent. Second, age of smoking initiation was derived from three variables: (1) self-reported smoking status (Data-Field 20116), (2) age at smoking initiation for current smokers (Data-Field 3436), and (3) age at smoking initiation for former smokers (Data-Field 2867). Based on prior research [12], participants were classified into four mutually exclusive groups: (a) never-smokers, (b) adulthood initiators ( > 18 years), (c) adolescent initiators (15–18 years), and (d) childhood initiators (5–14 years).

### Case identification for depression and anxiety disorders

Case ascertainment at baseline and follow-up was conducted using hospital admission records, death registry data, self-reported medical conditions, and primary care clinical records. Depression cases were identified using ICD-10 codes F32-F33 (depressive disorders) from the UK Biobank’s first occurrence fields (Data Category 1712), while anxiety disorders were identified using ICD-10 codes F40-F41 (phobic anxiety and other anxiety disorders). We removed individuals with: (a) depression or anxiety diagnoses recorded in ICD-10 fields (Data-Field 41202 and 41204); or (b) a self-reported history of depression or anxiety (Data-Field 20002) at baseline. The validity of depression and anxiety diagnoses using these data sources has been partially confirmed through comprehensive clinical assessments, with a positive predictive value exceeding 80%.

### Assessment of inflammatory markers

Nine inflammatory markers were selected for their relevance in distinct inflammatory pathways [24]. Blood samples were collected during the UK Biobank baseline visit (2006–2010). Complete blood count analyses were conducted using the Beckman Coulter LH750, measuring total leukocytes, neutrophils, platelets, monocytes, and lymphocytes, along with their respective proportions within leukocytes. Serum CRP was assessed via high-sensitivity immunoturbidimetric assay on the Beckman Coulter AU5800. Biospecimen processing protocols are publicly available in UK Biobank Resource 100080. To account for skewness and enable parametric analyses, biomarker distributions were log-transformed and *z*-score standardized.

### Brain imaging data and calculation of SF coupling

Brain imaging generated measures of functional connectivity (FC) and structural connectivity (SC). FC was assessed using resting-state functional magnetic resonance imaging (fMRI) time series for 148 cortical and 16 subcortical regions extracted from the UK Biobank using the Destrieux atlas [25] for cortical parcellation (Data-Field 31014) and the Tian subcortical atlas [26] for subcortical structures (Data-Field 31019). Individual-level time series data were used to construct 164 × 164 region-of-interest (ROI) FC matrices. SC was assessed using precomputed, individual level 164 × 164 ROI-to-ROI SC matrices from the UK Biobank (Data-Field 31014), derived from diffusion tensor imaging (DTI) data using the same parcellation schemes as those used in the measure of FC. Details on data acquisition and preprocessing methods can be found in the UK Biobank resources [27], as well as Supplementary Information.

**Fig. 1:**
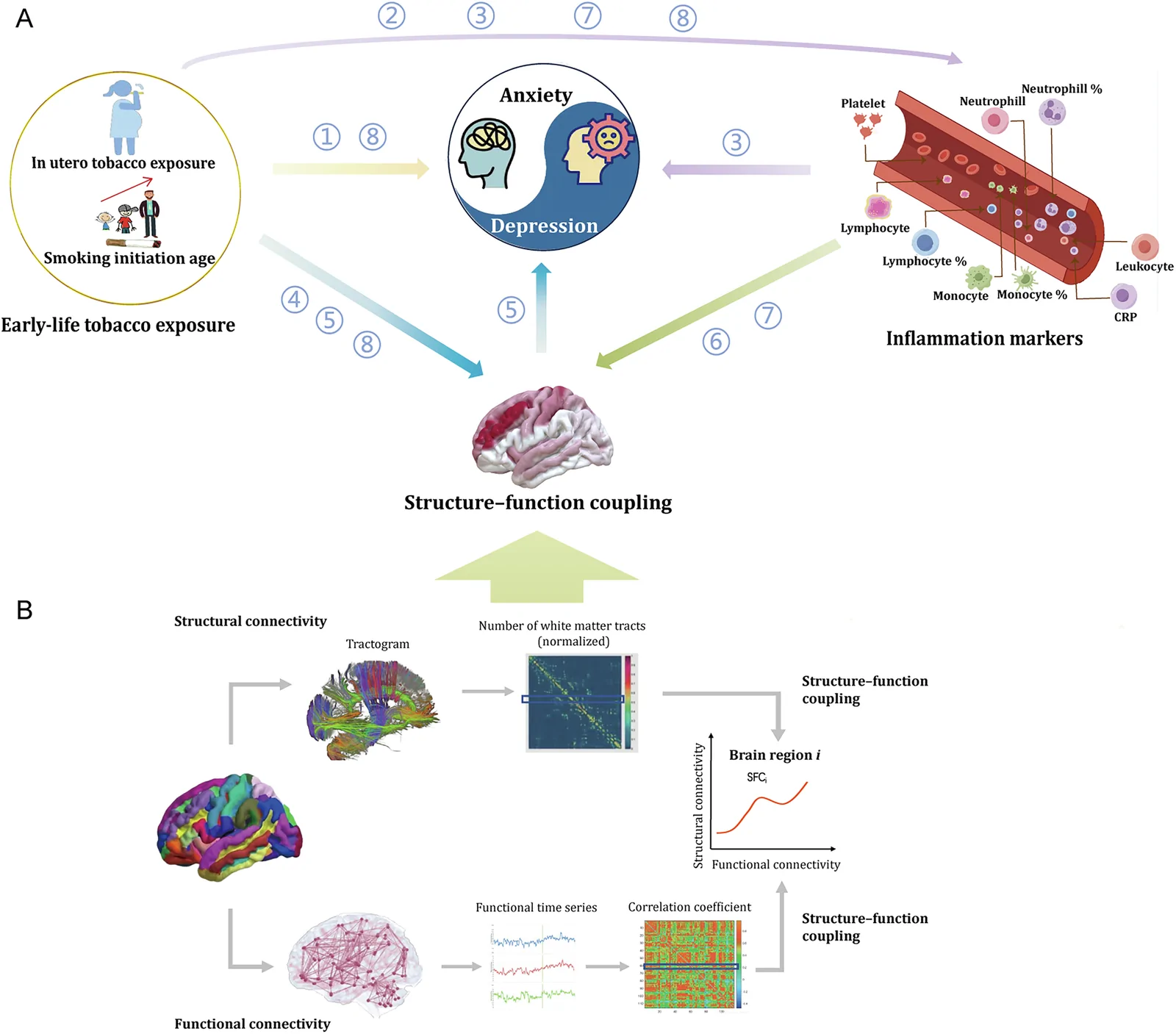
Analytical framework. **A** Schematic illustration of the comprehensive methodological approach. This study examines the association between early-life tobacco exposure—including in utero tobacco exposure and age of smoking initiation—and the risk of anxiety or depressive disorders. Eight pathways were studied: ① Early-life tobacco exposure → anxiety/depression; ② Early-life tobacco exposure → inflammation; ③ Early-life tobacco exposure → inflammation → anxiety/depression; ④ Early-life tobacco exposure → structure-function (SF) coupling; ⑤ Early-life tobacco exposure → SF coupling → anxiety/depression; ⑥ Inflammation → SF coupling; ⑦ Early-life tobacco exposure → inflammation → SF coupling; ⑧ Interaction effects: in utero tobacco exposure x age of smoking initiation on depression/anxiety incidence, inflammation and regional SF coupling. **B** Workflow for quantifying brain SF coupling. Structural connectivity is determined by reconstructing white matter fiber tracts followed by generation of a whole-brain structural connectivity matrix. Matrix rows and columns represent brain regions, with the normalized number of connecting fiber tracts reflecting connection strength (higher values indicate stronger structural connectivity). Functional connectivity is assessed by extracting time-series signals from brain regions and computing a functional connectivity matrix, where the statistical correlation between time series captures functional connectivity strength. SF coupling for each brain region *i* (SF coupling *i*) is calculated as the correlation coefficient between its structural and functional connectivity vectors.

We quantified SF coupling for each of the 164 brain regions (Fig. 1B). First, we extracted the nonzero structural connections of each region to form a SC vector and rescaled these values to an approximately Gaussian distribution with a mean of 0.5 and a standard deviation of 0.1 [28]. Second, we extracted the corresponding FC values as a vector. Finally, we computed the Pearson correlation between these two vectors to quantify the relationship between structural and functional connectivity [21].

### Assessment of covariates

All covariates were selected based on prior literature [12, 24], and each has been individually implicated in depression and anxiety [24]. Most information was self-reported; exceptions are noted below. Age at baseline was calculated from birthdate (Data-Field 21003) and categorized as < 65 years (middle-aged) vs. ≥ 65 years (older adults) based on UK retirement thresholds. Sex (Data-Field 31) was coded as male/female. Ethnicity (Data-Field 21000) was coded as White or ethnic minority. Education level (Data-Field 6138) was dichotomized as university/college degree vs. no degree. Annual household income (Data-Field 738) was classified as low, middle, and high. Alcohol intake (Data-Field 1558) was categorized as never vs. ever drinkers. The Townsend deprivation index (Data-Field 22189) was the sum of four census-derived components (unemployment, non-car ownership, overcrowded household, and non-home ownership), with higher scores indicating greater deprivation. BMI (Data-Field 21001) was calculated in the baseline assessment as a function of weight and height (kg/m^2^) and categorized as normal weight, overweight, and obesity. Because there was substantial missingness (44.9%) in birth weight (Data-Field 20022), we instead used a correlate of birth weight, namely childhood body composition, as a covariate [12]. This variable was based on parent-reported body habitus at age 10. Childhood body size at age 10 (Data-Field 1687) was categorized as about average, plumper, or thinner, while childhood height at age 10 (Data-Field 1697) was classified as about average, shorter, or taller.

### Statistical analysis

Figure 1A summarizes the overall analytical strategy. We used Cox proportional hazards models to examine the longitudinal association between early-life tobacco exposure and the incidence of depression and anxiety, with exposure-specific reference groups. Differences in inflammatory marker levels and SF coupling across exposure groups were assessed using t-tests and ANCOVA, with covariate adjustment and post hoc comparisons. Additionally, we investigated whether in utero tobacco exposure interacted with age of smoking initiation to influence depression/anxiety risk, inflammation levels and regional SF coupling.

We used Cox proportional hazards models to examine the linear associations between early-life tobacco exposure–related inflammatory markers and SF coupling with depression and anxiety risk. To assess potential nonlinear effects, we also conducted tertile-based analyses using the lowest tertile as the reference. We further examined associations between inflammatory markers and regional SF coupling using linear regression models. Multiple comparisons were addressed using Bonferroni correction for inflammatory markers and FDR adjustment for SF coupling, given the number of brain regions tested.

Mediation analyses were conducted using the R “mediation” package to test whether inflammatory markers and SF coupling mediated the association between early-life tobacco exposure and the incidence of depression and anxiety. Additional mediation models tested the pathway from exposure to SF coupling via inflammation. Stratified analyses were performed to assess covariate moderation, with interaction terms included to test effect modification. Details are provided in the Supplementary Materials.

## Results

### Participant characteristics at baseline

eTable 1 presents participant characteristics by early-life tobacco exposure status. Individuals with in utero tobacco exposure were more likely to be middle-aged, male, and White; have lower socioeconomic status (e.g., greater deprivation, lower income, and less educational attainment); be overweight or obese; report alcohol use; and describe themselves as shorter and plumper at age 10. Early smoking initiation was similarly associated with lower socioeconomic status, higher BMI, and being middle-aged, female, and White.

### Cox regression of early-life tobacco exposure and depression/anxiety risk

**Fig. 2:**
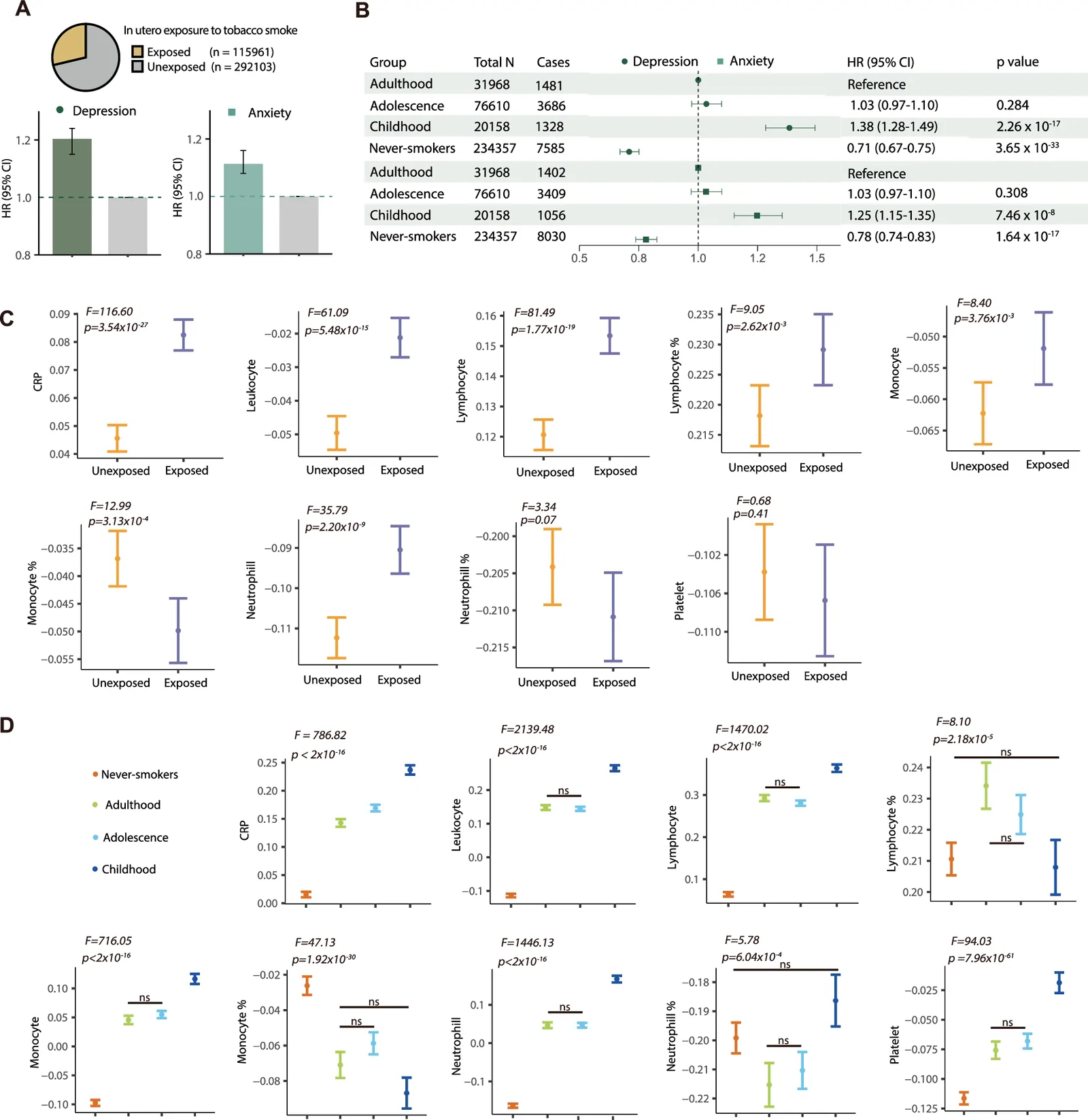
Associations between early-life tobacco exposure and risks for depression, anxiety, and inflammatory markers. **A** and **B** show hazard ratios with 95% confidence intervals. **C** and **D** show adjusted group means with 95% confidence intervals. **A** Results of Cox proportional hazards models with in utero tobacco exposure as predictor of adult depression and anxiety. **B** Results of Cox proportional hazards models examining age of smoking initiation as a predictor of adult depression and anxiety, using adulthood initiators as the reference group. **C** Comparison of Inflammatory marker levels in individuals exposed to tobacco in utero with unexposed individuals. **D** Inflammatory marker levels by age of smoking initiation. Comparisons marked ns are non-significant; all others are significant.

In utero tobacco exposure was significantly associated with elevated risk of both depression and anxiety (Fig. 2A). When using adulthood initiation as the reference, only childhood initiation remained significantly associated with increased risk of depression and anxiety (Fig. 2B). Compared with never-smokers, individuals who initiated smoking during childhood or adolescence exhibited increased psychiatric risk (eTable 2–3). All associations remained significant after Bonferroni correction.

### Associations between early-life tobacco exposure, inflammatory markers, and depression/anxiety risk

As shown in Fig. 2C, in utero tobacco exposure was associated with elevated levels in seven of nine inflammatory markers. Meanwhile, individuals who initiated smoking in childhood exhibited significantly elevated levels of most inflammatory markers—except monocyte percentage—compared to those who initiated in adulthood (Fig. 2D). In contrast, adolescent initiation differed from adulthood initiation only in CRP levels. All associations remained significant after Bonferroni correction.

**Fig. 3:**
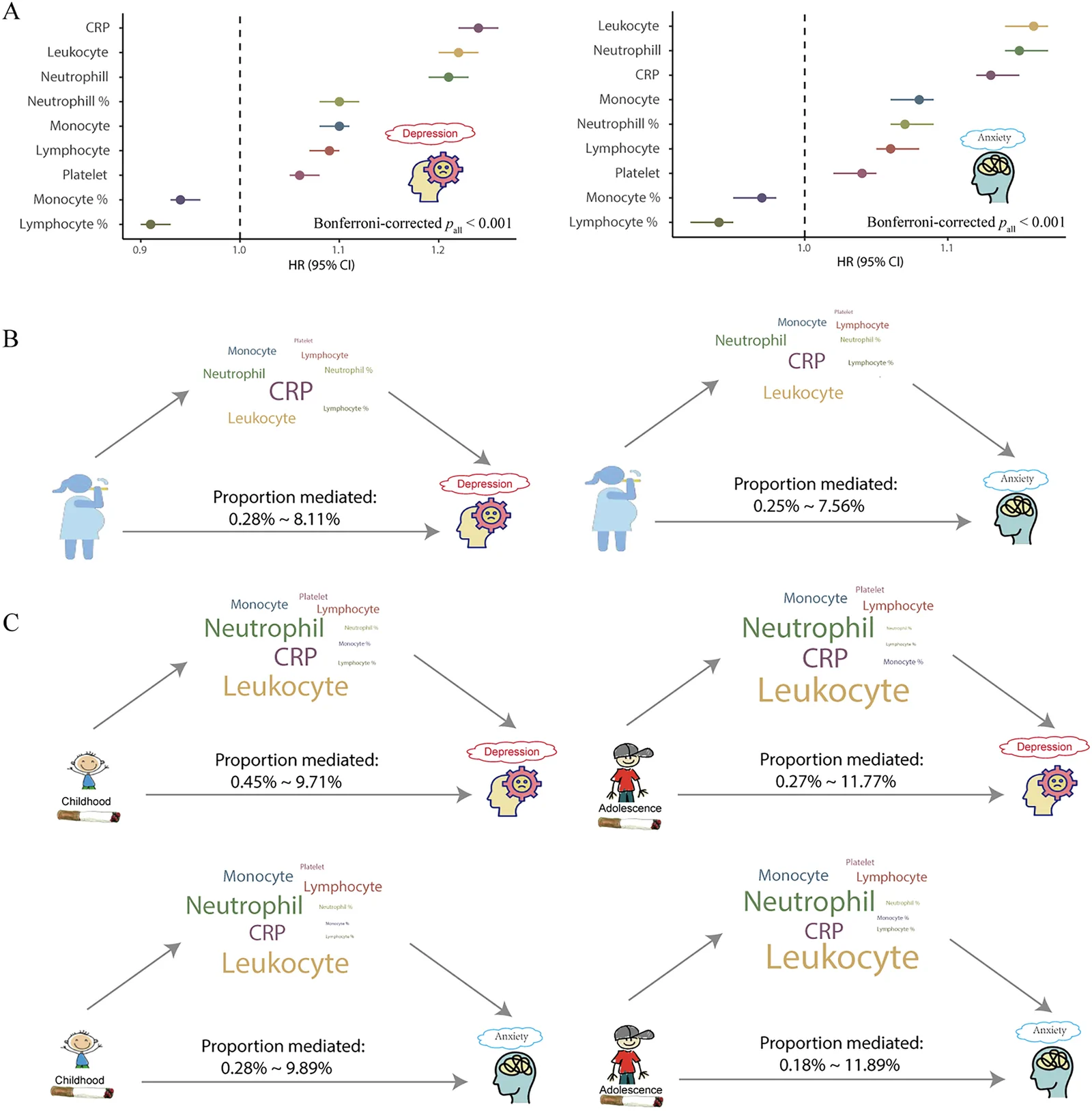
Longitudinal associations between inflammatory markers and the incidence of depression and anxiety. **A** Cox proportional hazards models showed significant linear associations between all nine inflammatory markers and incident depression/anxiety. Dots represent mean hazard ratios (HRs); horizontal lines indicate 95% confidence intervals (CIs). **B** Mediation effects of inflammatory markers on the association between in utero tobacco exposure and incident depression/anxiety. Linear regression was used to test the exposure–inflammation path and Cox models for the inflammation–outcome path. In the word cloud, font size reflects strength of the mediation effect. **C** Mediation effects of inflammatory markers on the association between early smoking initiation and incident depression/anxiety. In the mediation models, the “childhood” and “adolescence” predictors represent contrasts with the never-smoking group.

All nine inflammatory markers were significantly associated with depression/anxiety incidence (Fig. 3A and eFigure 1). Eight markers—excluding monocyte%—significantly mediated the effect of in utero tobacco exposure on both outcomes, with CRP showing the strongest mediation (Fig. 3B; eTable 4–5). Moreover, all markers mediated the associations between early smoking initiation and depression/anxiety (Fig. 3C; eTable 6–7). Additional details on inflammatory mediation effects are provided in the Supplementary Materials.

### Associations between in utero tobacco exposure, brain SF coupling, and depression/anxiety risk

**Fig. 4:**
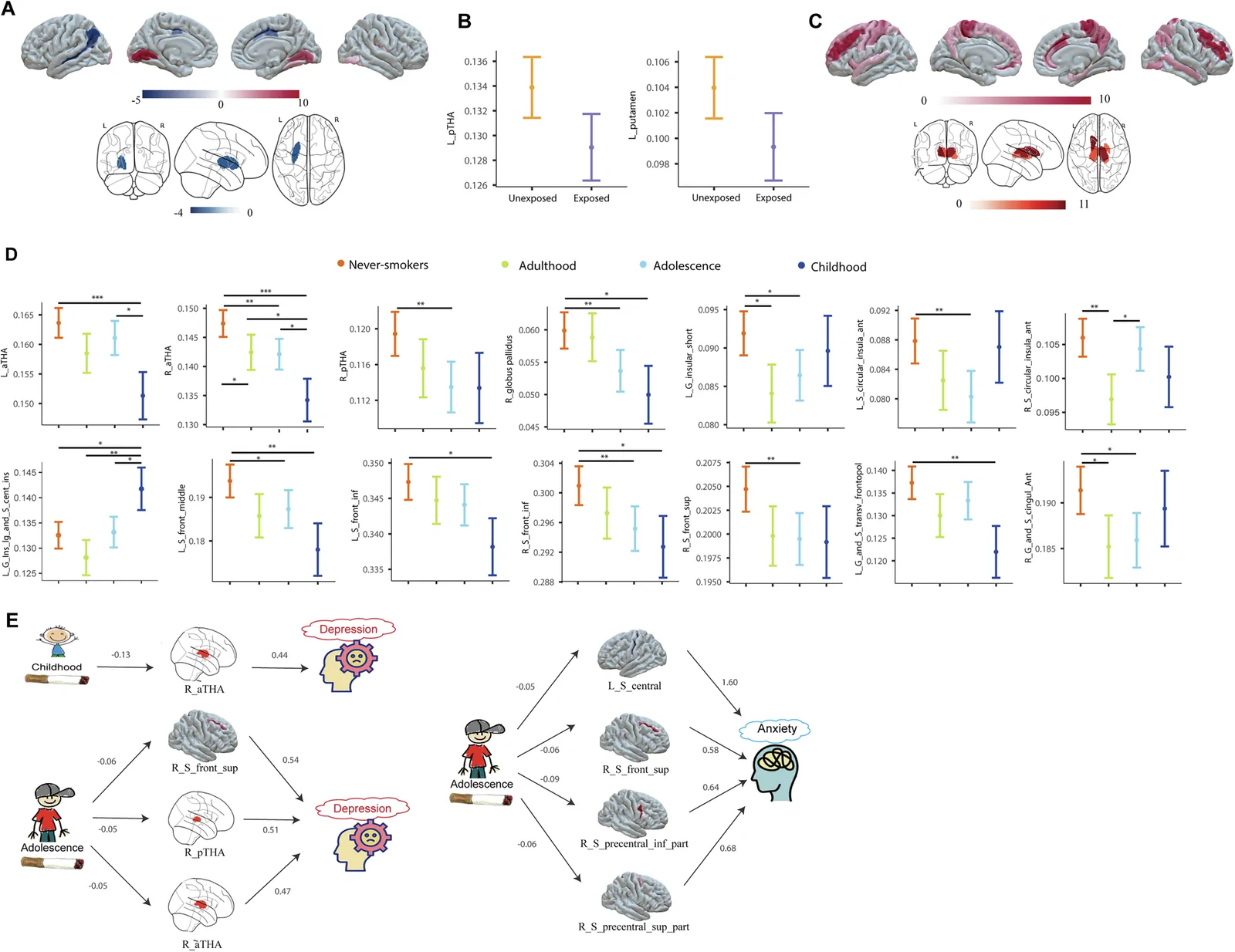
Associations between early-life tobacco exposure and brain SF coupling. **A** After whole-brain FDR correction (*p* < 0.05), brain regions showing significant SF coupling differences between individuals exposed to tobacco in utero and unexposed controls are displayed on the cortical surface and glass brain for subcortical regions. The color bar represents *t* values. Seventeen regions involved in reward processing, visual processing, and higher-order cognitive functions showed significant differences. L, left; R, right. **B** Mean values with 95% confidence intervals depict SF coupling in the left thalamus and left putamen for individuals exposed and unexposed to in utero tobacco. **C** Brain regions showing significant SF coupling differences by age of smoking initiation, after whole-brain FDR correction. The color bar represents *F* values. Significant differences were observed in 50 regions, including the anterior and posterior thalamus, caudate nucleus, and multiple frontal regions. **D** Mean values with 95% confidence intervals illustrate group differences in SF coupling across smoking initiation age groups in brain regions involved in reward and emotion processing. Asterisks indicate significant group differences (*p < 0.05; **p < 0.01; ***p < 0.001). Full pairwise post hoc comparison p-values for all regions are reported in eTable 13. **E** Analysis of regional SF coupling as a mediator of the association between smoking initiation age and depression/anxiety incidence. Pathways are illustrated with standardized regression coefficients (exposure–SF coupling path) and hazard ratios (SF coupling–outcome path). Only significant mediation models are presented. In these models, the predictors “childhood” and “adolescence” reflect contrasts with the never-smoking group.

Among 37,331 participants with complete SF coupling data, in utero tobacco exposure was associated with altered SF coupling in 17 brain regions, including reduced coupling in the left posterior thalamus and left putamen (Fig. 4A–B; eTable 8). Four of these regions were linked to depression incidence, and one—the right inferior precentral sulcus—was associated with anxiety (eTable 9–10; eFigure 2A-B).

Mediation analyses identified that SF coupling in the left superior temporal sulcus, right inferior occipital gyrus and sulcus and right posterior lateral fissure significantly mediated the association between in utero tobacco exposure and depression incidence (eTable 11). No significant mediation effects were observed for anxiety.

### Associations between age of smoking initiation, brain SF coupling, and the incidence of depression and anxiety

One-way ANCOVA revealed a significant effect of smoking initiation age on SF coupling in 50 brain regions, including the thalamus, caudate, and frontal areas (Fig. 4C-D; eTable 12). Post hoc tests showed that both childhood (23 regions) and adolescent smoking initiation (30 regions) were associated with altered SF coupling compared to never-smokers (eTable 13), particularly in networks related to reward, cognitive control, and sensory processing. Additional group differences across childhood, adolescent, and adulthood initiation groups were observed in a few regions, most notably the anterior thalamus and insula (Fig. 4D; eTable 13). All results remained significant after FDR correction.

Among the 50 regions identified in prior analyses, six were associated with depression and five with anxiety incidence (eFigure 2C-D; eTable 14–15). For depression, SF coupling in the right anterior thalamus significantly mediated risk among childhood initiators, while three frontal and thalamic regions showed mediation among adolescent initiators (Fig. 4E; eTable 16–17). No mediation effects were found among adulthood initiators. For anxiety, significant mediation was observed only among adolescent initiators, involving a frontoparietal network including the left central sulcus, right superior frontal sulcus, and right inferior/superior precentral sulcus (Fig. 4E; eTable 16–17).

### Inflammation as a mediator of the association between early exposure and SF coupling

After controlling for covariates and using FDR correction, six inflammatory markers—CRP, leukocyte count, lymphocyte percentage, lymphocyte count, monocyte count, and neutrophil count—showed significant associations with SF coupling in brain regions previously identified as affected by in utero tobacco exposure (eTable 18). These regions included the left angular gyrus, bilateral lingual gyrus, left superior temporal sulcus, right precentral gyrus, right posterior collateral transverse sulcus, right posterior lateral fissure, right mid-posterior cingulate gyrus and right inferior occipital gyrus and sulcus. The proportion of variance due to the mediating effect of inflammatory pathways in the association between in utero tobacco exposure and SF coupling ranged from 0.41–2.99% (eTable 19). The strongest mediation occurred when in utero tobacco exposure elevated CRP levels, subsequently reducing SF coupling in the right inferior precentral sulcus.

Similarly, there were significant associations between the nine inflammatory markers and SF coupling in regions affected by earlier smoking initiation. These regions encompassed the thalamus, caudate, middle and superior parts of frontal gyri, inferior parietal lobule, precentral gyrus, and anterior cingulate cortex (eTable 20).

Mediation analysis revealed that smoking initiation during childhood, adolescence, and adulthood influenced frontal pathways, the left caudate, and precentral regions through inflammatory markers, particularly C-reactive protein (CRP) and lymphocyte (eTable 21-23). The proportion of mediated variance ranged from 3.81–11.69% for childhood initiation, 1.39–11.22% for adolescent initiation, and 2.07–8.04% for adulthood initiation. CRP levels mediated the association between childhood or adolescent initiation (but not adulthood initiation) and SF coupling in the bilateral inferior frontal cortex. Notably, across different initiation periods, childhood smoking initiation exhibited the highest proportion of inflammation-mediated effects on SF coupling, particularly in the middle frontal cortex, superior frontal cortex, superior precentral cortex, and caudate (eFigure 3C).

### Interaction between in utero tobacco exposure and early smoking initiation

**Fig. 5:**
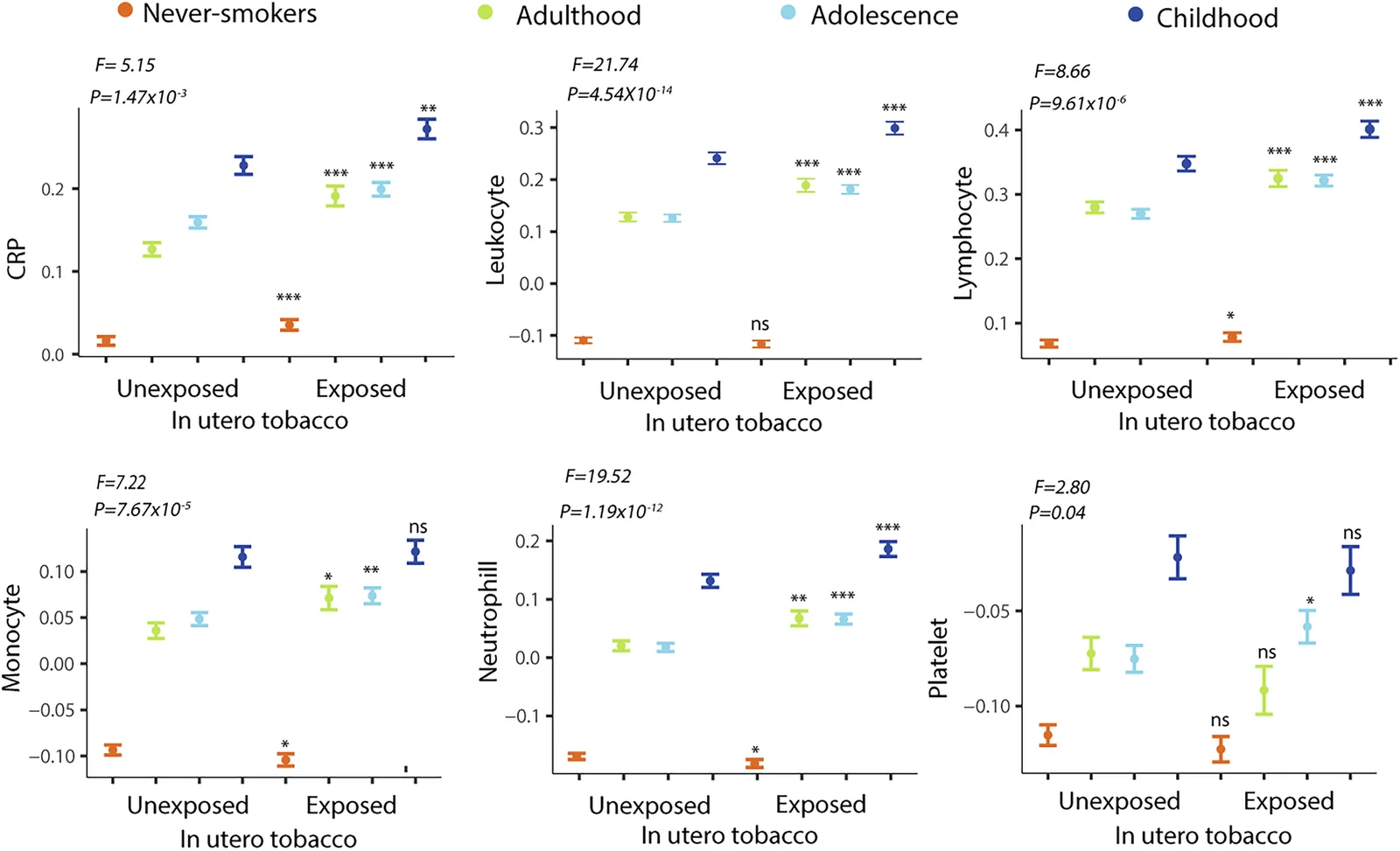
Interactive effects of in utero tobacco exposure and smoking initiation age on inflammatory markers. Each panel shows one inflammatory marker, presenting group means with 95% confidence intervals. Only markers with significant interaction effects between in utero tobacco exposure and smoking initiation age are shown, including CRP, leukocyte count, lymphocyte count, monocyte count, neutrophil count, and platelet count (F and p values are reported in each panel). Asterisks indicate whether significant differences exist between individuals exposed and unexposed to in utero tobacco exposure within each smoking initiation age group (*p < 0.05; **p < 0.01; ***p < 0.001; ns not significant, p > 0.05).

A significant interaction was found between in utero tobacco exposure and smoking initiation across six inflammatory markers (Fig. 5; eTable 24). In utero tobacco exposure amplified the pro-inflammatory effects of early smoking initiation, with the highest levels of inflammation observed in individuals exposed both prenatally and during childhood (Fig. 5). However, no significant interaction was found for depression or anxiety incidence, nor for SF coupling in any brain region.

### Stratified analyses

Analyses that stratified the sample by participant characteristics showed that the age of smoking initiation interacted with several covariates to predict mental health outcomes. The association between smoking initiation and depression was moderated by BMI (*p* = 2.57 × 10⁻¹⁴), ethnicity (*p* = 1.59 × 10⁻⁶), alcohol use (*p* = 4.04 × 10⁻⁴), deprivation (*p* = 1.06 × 10⁻³), and age (*p* = 2.69 × 10⁻³) (eTable 25; eFigure 4A). The association between smoking initiation and anxiety was moderated by deprivation (*p* = 1.23 × 10⁻³), alcohol use (*p* = 4.16 × 10⁻³), and ethnicity (*p* = 4.44 × 10⁻³) (eTable 26; eFigure 4B). No covariates moderated the relationship between maternal smoking during pregnancy and mental health outcomes.

Additionally, the effects of early-life tobacco exposure on inflammation levels (eTable 27–28) appeared to be influenced by multiple covariates. However, only BMI showed a significant interaction with early-life tobacco exposure on SF coupling in the right insula and left ACC (eTable 29). No other significant moderating effects were observed after Bonferroni correction.

## Discussion

Using the UK Biobank cohort, we identified robust dose-response associations between early-life tobacco exposure—including in utero tobacco exposure and early smoking initiation—and elevated risk of depression and anxiety in mid-to-late adulthood. Prior findings on the psychiatric consequences of in utero tobacco exposure have been mixed [29]. While some studies link prenatal tobacco exposure to later mood disorders [30], others report null or even protective effects [31]. Our analysis confirmed that in utero tobacco exposure significantly increased psychiatric risk over time. Notably, smoking initiation during childhood conferred the highest vulnerability, whereas initiation during adolescence or adulthood did not differ in psychiatric outcomes.

Our multi-marker approach revealed widespread immunomodulatory effects of early-life tobacco exposure. In utero tobacco exposure was associated with elevated levels of several inflammatory markers, likely reflecting chronic low-grade inflammation driven by fetal immune dysregulation and epigenetic modifications [6, 7, 32, 33]. These findings extend prior evidence of lasting immune alterations following maternal smoking, which were often based on relatively small samples [6, 7]. Similar to the depression and anxiety results, childhood smoking initiation—compared to adulthood initiation—was also associated with widespread inflammatory elevations, whereas adolescent initiation differed from adulthood initiation only in CRP levels. This may reflect both increased biological sensitivity during immune system maturation in childhood [34] and the cumulative burden of prolonged tobacco exposure [35, 36]. Inflammatory markers significantly mediated the association between early-life tobacco exposure and later depression and anxiety, aligning with prior research [37–39] and reinforcing the role of immune mechanisms in linking early environmental exposures to psychiatric risk.

In parallel, our neuroimaging analyses provide the first evidence that in utero tobacco exposure is associated with reduced SF coupling in key regions, including the left thalamus and left putamen. Early life is a sensitive window for brain development [40], during which prenatal nicotine exposure can disrupt neurotransmitter systems and induce oxidative damage, leading to long-term alterations in learning, memory, and reward processing [41, 42]. The thalamus, a central integrative hub [43], and the caudate, critical for reward learning and habit formation [44], showed functional impairments, consistent with previous reports of reduced caudate volume [45] and altered reward-related activation [19, 46], following early tobacco exposure.

Extending beyond prenatal exposure, we also found that individuals who initiated smoking during childhood or adolescence exhibited reduced SF coupling in prefrontal, cingulate, thalamic, and insular regions, compared with never-smokers. The heightened plasticity during these developmental stages may increase vulnerability to nicotine’s neurotoxic effects [47], potentially mediated by nicotinic acetylcholine receptors (nAChRs), which are highly expressed early in life and implicated in disrupted circuit maturation and accelerated neural aging [48, 49]. While prior studies have linked adolescent smoking to structural reductions in frontal and insular regions [18], our findings suggest that SF coupling may reveal functional alterations not captured by morphology alone. Notably, childhood and adolescent smoking initiation were differentially associated with SF coupling in the insula and anterior thalamus, potentially reflecting stage-specific plasticity in response to nicotine and highlighting distinct neurodevelopmental consequences of exposure during different sensitive periods.

Reductions in SF coupling in key brain regions mediated the relationship between early-life tobacco exposure (in utero or early initiation) and the incidence of depression and anxiety. This aligns with prior evidence linking reduced SF coupling in cognitive and emotional circuits to increased psychiatric risk [23, 50] and points to a plausible neural pathway. Further, immune system overactivity can impair brain structure and function by disrupting connectivity, plasticity, and neurotransmitter balance via effects on the blood–brain barrier [13]. Our mediation analyses showed that early-life tobacco exposure influences SF coupling through inflammation. In utero tobacco exposure may alter fetal brain development by allowing inflammation to cross the immature blood–brain barrier and suppress neural progenitor proliferation. Childhood smoking showed the strongest inflammation-mediated effects on SF coupling, particularly in frontal regions and the caudate, highlighting childhood as a critical window of vulnerability [51]. This study provides the first evidence of a biological pathway linking early tobacco exposure, inflammation, and disrupted brain network integration.

Notably, individuals exposed both in utero and during early childhood exhibited the greatest inflammatory burden, suggesting a compounding “double-hit” effect of early-life tobacco exposure. This may reflect synergistic impacts of prenatal immune programming [7, 33] and postnatal immune stimulation, possibly reinforcing early nicotine dependence via dopaminergic pathways [36, 52]. Together, these findings highlight a cycle of exposure, behavior, and inflammation that may heighten long-term psychiatric and physical health risks. Although we did not observe a significant interaction between in utero tobacco exposure and early smoking initiation on brain SF coupling, it remains possible that inflammation mediates their joint impact on neural circuitry [13].

This study has several limitations that warrant consideration. First, there could have been recall bias because early-life tobacco exposure was retrospectively self-reported. However, prior research has shown substantial concordance between offspring-reported in utero tobacco exposure and maternal reports of smoking during pregnancy [12, 53]. Moreover, due to social desirability biases, self-reported tobacco exposure is more likely to be underestimated than overestimated, suggesting that our observed associations may be conservative estimates of the true effects. Second, the UK Biobank dataset lacks detailed information on smoking quantity, duration of exposure, and secondhand exposure in early life. This limitation constrains our ability to conduct finer-grained dose-response analyses and to distinguish different modes of tobacco exposure. Third, while our findings suggest that high inflammation and low SF coupling mediate the relationship between early-life tobacco exposure and the risk of depression and anxiety, the cross-sectional nature of these associations does not fully establish causality, although the temporal precedence of exposure supports a causal interpretation. Mendelian randomization or intervention cohort studies could test the causal mediating role of these biomarkers. Fourth, only systemic inflammatory markers were assayed; no neuroinflammatory markers (e.g., microglial activation or central cytokines) were available. Thus, the extent to which peripheral inflammation reflects central neuroinflammation remains unclear. Future studies incorporating central immune measures are needed. Finally, the UK Biobank sample consists predominantly of middle-aged and older adults of European ancestry with relatively high socioeconomic status. This demographic homogeneity limits the generalizability of our findings to more diverse populations across different age groups, racial/ethnic backgrounds, and cultural contexts. Future research should aim to replicate these findings in more heterogeneous and representative cohorts.

## Conclusions

Using UK Biobank data, we found that in utero tobacco exposure increased depression/anxiety risk, elevated inflammation, and reduced SF coupling in the thalamus and putamen. Childhood smoking initiation was linked to greater psychiatric risk and inflammation than adulthood initiation, while adolescent and adulthood initiation showed no significant difference. In utero tobacco exposure further amplified the inflammatory effects of early smoking. Compared to adolescent initiation, childhood initiation was linked to lower SF coupling in the thalamus and higher coupling in the insula. These findings underscore the need for early tobacco prevention and highlight inflammation and SF coupling as potential intervention targets.

## Materials availability

The comprehensive dataset underpinning the conclusions of this investigation is accessible to qualified researchers through the UK Biobank data repository (www.ukbiobank.ac.uk), subject to completion of their standardized application protocol. Custom computational scripts and analytical code essential for replicating our findings are available from the corresponding author upon reasonable request. The sections 'Materials availability' and 'Data availability' in this proof contain identical duplicate text. To streamline the layout and comply with journal formatting guidelines, you may need to merge them and remove one redundant paragraph.

## Supplementary information

Supplementary Materials for Stage-Specific Effects of Early Tobacco Exposure on Mental Health, Inflammation, and Brain Coupling

## Data Availability

The comprehensive dataset underpinning the conclusions of this investigation is accessible to qualified researchers through the UK Biobank data repository (www.ukbiobank.ac.uk), subject to completion of their standardized application protocol. Custom computational scripts and analytical code essential for replicating our findings are available from the corresponding author upon reasonable request.
