# Supplementary material for: Stage-specific effects of early-life tobacco exposure on mental health, inflammation, and brain structure-function coupling
Source: Transl Psychiatry. 2026 Jul 10;16:518. doi: 10.1038/s41398-026-04182-4 (PMC13639059; doi:10.1038/s41398-026-04182-4)
Supplement: Supplementary file 1 [file 41398_2026_4182_MOESM1_ESM.docx]

Supplementary Materials for

**Stage-Specific Effects of Early Tobacco Exposure on Mental Health, Inflammation, and Brain Coupling**

**This file includes:**

eSupplementary Texts

1. MRI Data Acquisition
2. Statistical analysis
3. Inflammation as mediator of the association between early exposure and SF coupling

eTables 1 to 30

eFigures 1 to 4

eReferences (1 to 2)

**eSupplementary Text**

1. **MRI Data Acquisition**

Resting-state and diffusion MRI data were acquired from the UK Biobank imaging cohort using Siemens 3T Skyra scanners (Siemens Healthcare, Erlangen, Germany) at three sites (Manchester, Newcastle, and Reading) following a harmonized acquisition protocol and a standard Siemens 32-channel RF receive head coil ([http://www.fmrib.ox.ac.uk/ukbiobank/protocol/V4_23092014.pdf](http://www.fmrib.ox.ac.uk/ukbiobank/protocol/V4_23092014.pdf)). Resting-state functional MRI (rs-fMRI) data were collected using a gradient-echo echo-planar imaging (GE-EPI) sequence with the following parameters: repetition time (TR) = 735 ms; echo time (TE) = 39 ms; voxel size = 2.4 × 2.4 × 2.4 mm³; matrix size = 88 × 88 × 64; flip angle = 52°; multiband acceleration factor = 8; no in-plane acceleration (iPAT); and fat saturation. Each rs-fMRI scan lasted 6 minutes. Diffusion-weighted imaging (DWI) was performed using a spin-echo EPI sequence with repetition time (TR) = 3600 ms and echo time (TE) = 92 ms. The acquisition included 10 baseline (b ≈ 0 s/mm²) volumes, 50 volumes with b = 1000 s/mm², and 50 volumes with b = 2000 s/mm², each acquired along distinct diffusion-encoding directions, yielding 100 unique directions. High‑resolution T1‑weighted structural images were acquired using a 3D magnetization‑prepared rapid gradient‑echo (MPRAGE) sequence with the following parameters: repetition time (TR) = 2000 ms; echo time (TE) = 2.01 ms; inversion time (TI) = 880 ms; flip angle = 8°; voxel size = 1.0 × 1.0 × 1.0 mm³; field of view = 256×256×208 mm³; GRAPPA acceleration factor = 2; and total acquisition time approximately 5 minutes. These parameters are consistent with the UK Biobank imaging protocol. All DWI volumes were acquired with an isotropic voxel size of 2 mm. The total scan time for DWI was 6 minutes and 32 seconds. Imaging data were preprocessed by the UK Biobank imaging team. Full details of the image processing and quality control (QC) pipeline are available in prior publications^1,2^.

2. **Statistical analysis**

Figure 1A provides a visual summary of the methodological approaches implemented throughout our research process. **To investigate the longitudinal association between early-life tobacco exposure and the incidence of depression and anxiety, we employed a Cox proportional hazards regression model, using follow-up time as the underlying time scale. Baseline mental disorder assessment served as the entry time, while the exit time was defined as the occurrence of an event or censoring due to death, loss to follow-up, or study completion. For analyses where in utero tobacco exposure was the independent variable, the unexposed group was used as the reference category. When examining age of smoking initiation, never-smokers and adulthood initiators were used as separate reference groups. The models were adjusted for gender, age, ethnicity, socioeconomic deprivation, educational attainment, family income, body mass index (BMI), alcohol status, and childhood body size/height.**

**We conducted independent t-tests to examine differences in inflammatory marker levels and SF coupling across 164 brain regions between individuals exposed and unexposed to in utero tobacco. Analysis of covariance (ANCOVA) was employed to assess differences in inflammatory markers and regional SF coupling across age of smoking initiation groups, with post hoc tests performed for pairwise comparisons. Additionally, we investigated whether in utero tobacco exposure interacted with age of smoking initiation to influence depression/anxiety risk, inflammation levels and regional SF coupling. The same set of covariates as previously listed was included in these analyses.**

**Using** **Cox proportional hazards models, we further treated inflammatory markers and regional SF coupling that showed significant associations with early-life tobacco exposure as continuous variables to examine their linear associations with the incidence of depression and anxiety. We also constructed models stratified by tertiles for each inflammatory marker and regional SF coupling, with the lowest tertile serving as the reference, to evaluate their nonlinear associations with the incidence of depression and anxiety. Meanwhile, we also examined the relationship between inflammatory markers and regional SF coupling using linear regression models. To correct for multiple comparisons, Bonferroni correction was applied for analyses of inflammatory marker levels, whereas the Benjamini-Hochberg false discovery rate (FDR) method was used for SF coupling analyses, given the large number of brain regions examined.**

**Mediation analyses were performed in R (“mediation” package) to test whether inflammatory levels and regional SF coupling mediated the association between early-life tobacco exposure and the incidence of depression and anxiety. Linear regression models were employed to assess the first segment of the mediation pathway, while survival regression models were used to evaluate the second segment involving the incidence of depression and anxiety. Additionally, we investigated whether inflammatory levels mediate the effect of early-life tobacco exposure on regional SF coupling, with both segments of this mediation pathway tested using linear regression models. For mediation models that reached statistical significance, we calculated the proportion of the effect mediated.**

**We further conducted a series of stratified analyses to assess whether each covariate moderated the associations of early-life tobacco exposure with depression and anxiety incidence, inflammatory marker levels, and regional SF coupling. Moderation was tested by including interaction terms between early-life tobacco exposure and each stratifying covariate in the models.**

3. **Inflammatory markers as mediators of the association between early exposure and depression/anxiety incidence**

Each of the nine inflammatory markers was significantly associated with the incidence of depression and anxiety (Figure 3A) after adjusting for covariates and applying Bonferroni correction . Specifically, CRP, leukocytes, and neutrophils exhibited relatively strong effects, with each standard deviation (SD) increase associated with around a 20% higher risk of depression and approximately a 15% higher risk of anxiety. Conversely, higher monocyte percentage and lymphocyte percentage were protective, with each SD increase linked to a 5%-9% lower risk of depression and about a 5% reduction in anxiety risk.

Additionally, non-linear associations were found between all nine inflammatory markers and the incidence of depression (eFigure 1A). Similar non-linear patterns were observed for eight of the nine markers, except neutrophils, in relation to incident anxiety (eFigure 1B). These non-linear patterns were further supported by analyses of the tertiles for each inflammatory marker , which revealed consistent trends across inflammatory markers for both depression and anxiety.

Eight of the nine inflammatory markers (excluding monocyte percentage) significantly mediated the relationship between in utero exposure and the incidence of both depression and anxiety (Figure 3B). For depression, the percent of variance explained by the mediation effect varied between 0.28% and 8.11%, with CRP levels having the strongest mediation effect. For anxiety, the percent of variance explained by the mediation effect ranged from 0.25% to 7.56%, again with CRP showing the strongest mediation effect.

Each of the nine inflammatory markers significantly mediated the association between early initiation and depression (Figure 3C). The proportion of variance due to mediation ranged from 0.45% (monocyte percentage) to 9.71% (leukocyte count) among childhood initiators, and from 0.27% (lymphocyte percentage) to 11.77% (leukocyte count) among adolescent initiators. Anxiety outcomes showed similar patterns, with the mediation effect explaining 0.28%-9.89% of the variance for childhood initiators and 0.18%-11.89% for adolescent initiators, with leukocyte, neutrophil and CRP identified as the strongest mediators in both age groups.

| **eTable 1. Demographic characteristics of participants stratified by early-life tobacco exposure** | | | | | | | | | | |
| --- | --- | --- | --- | --- | --- | --- | --- | --- | --- | --- |
| **Characteristic** | **Level** | **In utero exposure to tobacco smoke** | | |  | **Age of smoking initiation** | | | | |
|  |  | **Unexposed (*n*=292103)** | **Exposed (*n*=115961)** | ***p* value** |  | **Adolescence (*n*=83246)** | **Adulthood**  **(*n*=34970)** | **Childhood (*n*=22163)** | **Never Smokers (*n*=255905)** | ***p* value** |
| Age group | Middle age | 231957 (79.4%) | 98378 (84.8%) | <.001 |  | 63942 (76.8%) | 25719 (73.5%) | 18518 (83.6%) | 211572 (82.7%) | <.001 |
|  | Older age | 60146 (20.6%) | 17583 (15.2%) |  |  | 19304 (23.2%) | 9251 (26.5%) | 3645 (16.4%) | 44333 (17.3%) |  |
| Gender | Male | 159321 (54.5%) | 60732 (52.4%) | <.001 |  | 37313 (44.8%) | 18530 (53%) | 7314 (33%) | 149706 (58.5%) | <.001 |
|  | Female | 132782 (45.5%) | 55229 (47.6%) |  |  | 45933 (55.2%) | 16440 (47%) | 14849 (67%) | 106199 (41.5%) |  |
| Ethnicity | Ethnic Minority | 23669 (8.1%) | 1936 (1.7%) | <.001 |  | 2235 (2.7%) | 2167 (6.2%) | 689 (3.1%) | 18602 (7.3%) | <.001 |
|  | White | 268434 (91.9%) | 114025 (98.3%) |  |  | 81011 (97.3%) | 32803 (93.8%) | 21474 (96.9%) | 237303 (92.7%) |  |
| Deprivation | Lower | 101798 (34.9%) | 37782 (32.6%) | <.001 |  | 25451 (30.6%) | 10071 (28.8%) | 5218 (23.6%) | 94373 (36.9%) | <.001 |
|  | Higher | 92784 (31.8%) | 39609 (34.2%) |  |  | 30388 (36.5%) | 13966 (40%) | 10370 (46.9%) | 74389 (29.1%) |  |
|  | Middle | 97171 (33.3%) | 38422 (33.2%) |  |  | 27325 (32.9%) | 10888 (31.2%) | 6540 (29.6%) | 86827 (34%) |  |
| Education | Less  than college | 186440 (64.3%) | 82527 (71.8%) | <.001 |  | 63448 (76.9%) | 21971 (63.4%) | 18487 (84.1%) | 161852 (63.7%) | <.001 |
|  | Above college | 103427 (35.7%) | 32469 (28.2%) |  |  | 19089 (23.1%) | 12668 (36.6%) | 3491 (15.9%) | 92144 (36.3%) |  |
| Family  average income | Unknown | 29469 (10.6%) | 10133 (9.1%) | <.001 |  | 7742 (9.8%) | 3036 (9.2%) | 1884 (9%) | 26946 (11.1%) | <.001 |
|  | High | 14783 (5.3%) | 5496 (5%) |  |  | 2760 (3.5%) | 1519 (4.6%) | 479 (2.3%) | 13659 (5.6%) |  |
|  | Low | 179893 (64.9%) | 73984 (66.8%) |  |  | 56576 (71.6%) | 22932 (69.4%) | 15684 (75.1%) | 152985 (63%) |  |
|  | Middle | 52928 (19.1%) | 21148 (19.1%) |  |  | 11950 (15.1%) | 5541 (16.8%) | 2844 (13.6%) | 49300 (20.3%) |  |
| BMI type | Normal | 102136 (35.2%) | 34480 (29.9%) | <.001 |  | 23251 (28.1%) | 10983 (31.6%) | 5157 (23.4%) | 91889 (36.1%) | <.001 |
|  | Overweight | 123518 (42.5%) | 49307 (42.7%) |  |  | 37102 (44.8%) | 14816 (42.6%) | 9529 (43.3%) | 106111 (41.7%) |  |
|  | Obesity | 64845 (22.3%) | 31623 (27.4%) |  |  | 22453 (27.1%) | 8964 (25.8%) | 7316 (33.3%) | 56594 (22.2%) |  |
| Alcohol status | Never | 24819 (8.5%) | 7318 (6.3%) | <.001 |  | 47606 (67.1%) | 22199 (73.8%) | 10815 (56.5%) | 165882 (72.6%) | <.001 |
|  | Alcohol use | 267053 (91.5%) | 108575 (93.7%) |  |  | 23344 (32.9%) | 7891 (26.2%) | 8322 (43.5%) | 62634 (27.4%) |  |
| Comparative body size at age 10 | About average | 148857 (52%) | 55086 (48.1%) | <.001 |  | 4833 (5.8%) | 2281 (6.5%) | 1708 (7.7%) | 23727 (9.3%) | <.001 |
|  | Plumper | 42598 (14.9%) | 20559 (18%) |  |  | 78357 (94.2%) | 32654 (93.5%) | 20423 (92.3%) | 231999 (90.7%) |  |
|  | Thinner | 94983 (33.2%) | 38844 (33.9%) |  |  | 41631 (50.9%) | 17801 (51.9%) | 10079 (46.4%) | 129277 (51.6%) |  |
| Comparative height at age 10 | About average | 156561 (54.6%) | 60355 (52.7%) | <.001 |  | 13495 (16.5%) | 5639 (16.4%) | 3903 (18%) | 38237 (15.3%) | <.001 |
|  | Shorter | 56389 (19.7%) | 25639 (22.4%) |  |  | 26662 (32.6%) | 10867 (31.7%) | 7754 (35.7%) | 83073 (33.2%) |  |
|  | Taller | 73999 (25.8%) | 28468 (24.9%) |  |  | 45087 (55%) | 18313 (53.3%) | 11500 (52.8%) | 136627 (54.5%) |  |

Data are presented as n (%). Column percentages are shown.

Abbreviations: BMI, body mass index.

| **eTable 2. Associations between age of smoking initiation and incident depression, adjusted for an extended set of covariates. Never-smokers served as the reference group** | | | | |
| --- | --- | --- | --- | --- |
| **Characteristic** | | **Participants** | **HR (95% CI)** | ***p* value** |
| Smoking initiation age |  |  |  |  |
| Never smokers |  | 234357 (64.5%) |  |  |
| Adolescence |  | 76610 (21.1%) | 1.46 (1.40-1.52) | <.001 |
| Adulthood |  | 31968 (8.8%) | 1.41 (1.33-1.49) | <.001 |
| Childhood |  | 20158 (5.6%) | 1.95 (1.84-2.07) | <.001 |
| Age group |  |  |  |  |
| Middle age |  | 295384 (81.4%) |  |  |
| Older age |  | 67709 (18.6%) | 1.13 (1.08-1.18) | <.001 |
| Gender |  |  |  |  |
| Male |  | 192058 (52.9%) |  |  |
| Female |  | 171035 (47.1%) | 0.69 (0.67-0.71) | <.001 |
| Education |  |  |  |  |
| Less than college |  | 241384 (66.5%) |  |  |
| Above college |  | 121709 (33.5%) | 0.81 (0.77-0.84) | <.001 |
| Ethnicity |  |  |  |  |
| Ethnic minority |  | 19143 (5.3%) |  |  |
| White |  | 343950 (94.7%) | 1.29 (1.19-1.40) | <.001 |
| Deprivation |  |  |  |  |
| Lower |  | 126700 (34.9%) |  |  |
| Higher |  | 114488 (31.5%) | 1.40 (1.35-1.46) | <.001 |
| Middle |  | 121905 (33.6%) | 1.09 (1.05-1.14) | <.001 |
| Family average income |  |  |  |  |
| Unknown |  | 36790 (10.1%) |  |  |
| High |  | 18135 (5.0%) | 0.44 (0.39-0.51) | <.001 |
| Low |  | 239796 (66.0%) | 1.14 (1.08-1.20) | <.001 |
| Middle |  | 68372 (18.8%) | 0.71 (0.66-0.76) | <.001 |
| BMI type |  |  |  |  |
| Normal |  | 121314 (33.4%) |  |  |
| Overweight |  | 155018 (42.7%) | 1.15 (1.10-1.20) | <.001 |
| Obesity |  | 86761 (23.9%) | 1.55 (1.48-1.62) | <.001 |
| Alcohol status |  |  |  |  |
| Never |  | 27460 (7.6%) |  |  |
| Alcohol use |  | 335633 (92.4%) | 0.66 (0.62-0.69) | <.001 |
| Comparative body size at age 10 |  |  |  |  |
| About average |  | 186370 (51.3%) |  |  |
| Plumper |  | 57058 (15.7%) | 1.14 (1.09-1.20) | <.001 |
| Thinner |  | 119665 (33.0%) | 1.17 (1.13-1.22) | <.001 |
| Comparative height at age 10 |  |  |  |  |
| About average |  | 197317 (54.3%) |  |  |
| Shorter |  | 73885 (20.3%) | 1.00 (0.95-1.04) | 0.825 |
| Taller |  | 91891 (25.3%) | 1.04 (1.00-1.08) | 0.058 |
| Data are presented as number of participants (n) and hazard ratio (HR) with 95% confidence interval (CI). Never‑smokers served as the reference group.  Abbreviations: BMI, body mass index; CI, confidence interval; HR, hazard ratio. | | | | |

| **eTable 3. Associations between age of smoking initiation and incident anxiety, adjusted for an extended set of covariates. Never-smokers served as the reference group** | | | | |
| --- | --- | --- | --- | --- |
| **Characteristic** | | **Participants** | **HR (95% CI)** | ***p* value** |
| Smoking initiation age |  |  |  |  |
| Never smokers |  | 234357 (64.5%) |  |  |
| Adolescence |  | 76610 (21.1%) | 1.32 (1.27-1.38) | <.001 |
| Adulthood |  | 31968 (8.8%) | 1.28 (1.21-1.36) | <.001 |
| Childhood |  | 20158 (5.6%) | 1.60 (1.50-1.71) | <.001 |
| Age group |  |  |  |  |
| Middle age |  | 295384 (81.4%) |  |  |
| Older age |  | 67709 (18.6%) | 1.28 (1.23-1.33) | <.001 |
| Gender |  |  |  |  |
| Male |  | 192058 (52.9%) |  |  |
| Female |  | 171035 (47.1%) | 0.59 (0.57-0.62) | <.001 |
| Education |  |  |  |  |
| Less than college |  | 241384 (66.5%) |  |  |
| Above college |  | 121709 (33.5%) | 0.82 (0.78-0.85) | <.001 |
| Ethnicity |  |  |  |  |
| Ethnic minority |  | 19143 (5.3%) |  |  |
| White |  | 343950 (94.7%) | 1.27 (1.17-1.38) | <.001 |
| Deprivation |  |  |  |  |
| Lower |  | 126700 (34.9%) |  |  |
| Higher |  | 114488 (31.5%) | 1.25 (1.20-1.30) | <.001 |
| Middle |  | 121905 (33.6%) | 1.04 (0.99-1.08) | 0.085 |
| Family average income |  |  |  |  |
| Unknown |  | 36790 (10.1%) |  |  |
| High |  | 18135 (5.0%) | 0.41 (0.36-0.47) | <0.001 |
| Low |  | 239796 (66.0%) | 1.01 (0.96-1.06) | 0.744 |
| Middle |  | 68372 (18.8%) | 0.68 (0.63-0.73) | <.001 |
| BMI type |  |  |  |  |
| Normal |  | 121314 (33.4%) |  |  |
| Overweight |  | 155018 (42.7%) | 1.07 (1.02-1.11) | 0.002 |
| Obesity |  | 86761 (23.9%) | 1.24 (1.19-1.30) | <.001 |
| Alcohol status |  |  |  |  |
| Never |  | 27460 (7.6%) |  |  |
| Alcohol use |  | 335633 (92.4%) | 0.73 (0.69-0.77) | <.001 |
| Comparative body size at age 10 |  |  |  |  |
| About average |  | 186370 (51.3%) |  |  |
| Plumper |  | 57058 (15.7%) | 1.08 (1.03-1.13) | 0.003 |
| Thinner |  | 119665 (33.0%) | 1.19 (1.15-1.24) | <.001 |
| Comparative height at age 10 |  |  |  |  |
| About average |  | 197317 (54.3%) |  |  |
| Sho rter |  | 73885 (20.3%) | 1.05 (1.00-1.09) | 0.041 |
| Taller |  | 91891 (25.3%) | 1.04 (1.00-1.09) | 0.042 |
| Data are presented as number of participants (n) and hazard ratio (HR) with 95% confidence interval (CI). Never‑smokers served as the reference group.  Abbreviations: BMI, body mass index; CI, confidence interval; HR, hazard ratio. | | | | |

| **eTable 4. Mediating effect of inflammatory markers on the relationship between in utero exposure to tobacco smoke and incident depression** | | | | | | | | |
| --- | --- | --- | --- | --- | --- | --- | --- | --- |
| **Inflammation mediator** | **Proportion Mediated (%)** | | | | **β ^a^** | ***p* value ^a^** | **HR ^b^** | ***p* value ^b^** |
|  | **Value** | **95% CI (Lower)** | **95% CI**  **(Upper)** | ***p* value** |  |  |  |  |
| CRP | 8.11 | 6.75 | 9.28 | <0.001 | .09 | 2.53x10^-128^ | 1.15 | 2.15x10^-33^ |
| Leukocyte | 5.24 | 4.23 | 6.15 | <0.001 | .06 | 5.08x10^-70^ | 1.13 | 2.55x10^-37^ |
| Lymphocyte | 1.21 | 0.89 | 1.59 | <0.001 | .04 | 5.59x10^-24^ | 1.05 | 3.02x10^-40^ |
| Lymphocyte % | 0.84 | 0.58 | 1.19 | <0.001 | -.02 | 8.01x10^-10^ | 0.94 | 7.47x10^-41^ |
| Monocyte | 1.66 | 1.16 | 2.07 | <0.001 | .04 | 2.11x10^-35^ | 1.06 | 4.72x10^-40^ |
| Monocyte % | 0.06 | -0.10 | 0.23 | .56 | <0.001 | .46 | 0.96 | 3.52x10^-41^ |
| Neutrophill | 4.57 | 3.93 | 5.39 | <0.001 | .06 | 8.79x10^-59^ | 1.13 | 6.46x10^-38^ |
| Neutrophill % | 0.82 | 0.52 | 1.19 | <0.001 | .02 | 5.93x10^-9^ | 1.06 | 7.63x10^-41^ |
| Platelet | 0.28 | 0.09 | 0.51 | <0.001 | .01 | 1.74x10^-3^ | 1.04 | 7.13x10^-41^ |
| Data are presented as proportion mediated (%) with 95% confidence interval (CI), β coefficient (from linear regression for exposure→mediator path), and hazard ratio (HR) from Cox model for mediator→outcome path.  Abbreviations: CI, confidence interval; CRP, C‑reactive protein; HR, hazard ratio.  a. Effect of the first half of the mediation pathway (regression analysis); b. Effect of the second half of the mediation pathway (hazard ratio). | | | | | | | | |

| **eTable 5. Mediating effect of inflammatory markers on the relationship between in utero exposure to tobacco smoke and incident anxiety** | | | | | | | | |  |
| --- | --- | --- | --- | --- | --- | --- | --- | --- | --- |
| **Inflammation mediator** | **Proportion Mediated (%)** | | | | **β ^a^** | ***p* value ^a^** | **HR** **^b^** | ***p* value ^b^** | |
|  | **Value** | **95% CI (Lower)** | **95% CI**  **(Upper)** | ***p* value** |  |  |  |  |  |
| CRP | 7.56 | 5.70 | 10.34 | <0.001 | .09 | 2.53x10^-128^ | 1.07 | 9.59x10^-15^ | |
| Leukocyte | 6.01 | 4.48 | 8.20 | <0.001 | .06 | 5.08x10^-70^ | 1.08 | 9.89x10^-15^ | |
| Lymphocyte | 1.50 | 1.00 | 2.18 | <0.001 | .04 | 5.59x10^-24^ | 1.03 | 5.37x10^-16^ | |
| Lymphocyte % | 0.88 | 0.51 | 1.36 | <0.001 | -.02 | 8.01x10^-10^ | 0.97 | 2.55x10^-16^ | |
| Monocyte | 2.21 | 1.47 | 3.02 | <0.001 | .04 | 2.11x10-^35^ | 1.04 | 6.94x10^-16^ | |
| Monocyte % | 0.06 | -0.08 | 0.21 | .44 | <0.001 | .46 | 0.98 | 1.90x10^-16^ | |
| Neutrophill | 5.24 | 4.15 | 7.02 | <0.001 | .06 | 8.79x10^-59^ | 1.07 | 4.32x10^-15^ | |
| Neutrophill % | 0.90 | 0.57 | 1.44 | <0.001 | .02 | 5.93x10^-9^ | 1.03 | 2.59x10^-16^ | |
| Platelet | 0.25 | 0.08 | 0.61 | <0.001 | .01 | 1.74x10^-3^ | 1.02 | 2.94x10^-16^ | |
| Data are presented as proportion mediated (%) with 95% confidence interval (CI), β coefficient (from linear regression for exposure→mediator path), and hazard ratio (HR) from Cox model for mediator→outcome path.  Abbreviations: CI, confidence interval; CRP, C‑reactive protein; HR, hazard ratio.  a. Effect of the first half of the mediation pathway(regression analysis); b. Effect of the second half of the mediation pathway. | | | | | | | | |  |

| **eTable 6. Mediating effect of inflammatory markers on the relationship between age of smoking initiation and incident depression** | | | | | | | | | | |  |
| --- | --- | --- | --- | --- | --- | --- | --- | --- | --- | --- | --- |
| **Smoking initiation age** | **Inflammation marker** | | **Proportion Mediated (%)** | | | | **β ^a^** | ***p* value ^a^** | **HR ^b^** | ***p* value ^b^** | |
|  |  |  | **Value** | **95% CI (Lower)** | **95% CI**  **(Upper)** | ***p* value** |  |  |  |  |  |
| Childhood | | CRP | 8.76 | 7.73 | 9.78 | <0.001 | .34 | <0.001 | 1.15 | 4.78x10^-133^ | |
| vs. | | Leukocyte | 9.71 | 8.32 | 10.98 | <0.001 | .45 | <0.001 | 1.12 | 8.80x10^-126^ | |
| Never smokers | | Lymphocyte | 2.10 | 1.25 | 2.94 | <0.001 | .33 | <0.001 | 1.03 | 1.69x10^-145^ | |
|  | | Lymphocyte % | 0.49 | 0.32 | 0.67 | <0.001 | -.04 | 3.09x10^-8^ | 0.94 | 2.03x10^-152^ | |
|  | | Monocyte | 2.37 | 1.68 | 3.07 | <0.001 | .26 | 2.19x10^-313^ | 1.05 | 8.78x10^-146^ | |
|  | | Monocyte % | 0.45 | 0.26 | 0.68 | <0.001 | -.07 | 1.08x10^-20^ | 0.97 | 7.80x10^-152^ | |
|  | | Neutrophill | 8.79 | 7.76 | 10.38 | <0.001 | .41 | <0.001 | 1.12 | 2.90x10^-126^ | |
|  | | Neutrophill % | 0.58 | 0.40 | 0.74 | <0.001 | .05 | 2.07x10^-10^ | 1.07 | 4.29x10^-152^ | |
|  | | Platelet | 0.89 | 0.64 | 1.17 | <0.001 | .12 | 1.19x10^-65^ | 1.04 | 3.83x10^-152^ | |
|  | |  |  |  |  |  |  |  |  |  | |
| Adolescence | | CRP | 9.27 | 8.18 | 10.64 | <0.001 | .21 | <0.001 | 1.13 | 5.86x10^-85^ | |
| vs. | | Leukocyte | 11.77 | 10.30 | 13.49 | <0.001 | .29 | <0.001 | 1.12 | 2.10x10^-76^ | |
| Never smokers | | Lymphocyte | 3.07 | 2.21 | 4.02 | <0.001 | .22 | <0.001 | 1.04 | 6.91x10^-91^ | |
|  | | Lymphocyte % | 0.27 | 0.11 | 0.44 | <0.001 | -.01 | <0.001 | 0.94 | 2.40x10^-97^ | |
|  | | Monocyte | 3.30 | 2.54 | 4.16 | <0.001 | .18 | <0.001 | 1.05 | 3.02x10^-91^ | |
|  | | Monocyte % | 0.35 | 0.22 | 0.53 | <0.001 | -.03 | 3.10x10^-13^ | 0.97 | 1.18x10^-96^ | |
|  | | Neutrophill | 9.70 | 8.42 | 10.98 | <0.001 | .25 | <0.001 | 1.11 | 4.64x10^-79^ | |
|  | | Neutrophill % | 0.27 | 0.09 | 0.43 | <0.001 | .01 | <0.001 | 1.06 | 2.98x10^-97^ | |
|  | | Platelet | 0.87 | 0.58 | 1.14 | <0.001 | .06 | 6.29x10^-49^ | 1.04 | 2.28x10^-96^ | |
|  | |  |  |  |  |  |  |  |  |  | |
| Adulthood | | CRP | 8.44 | 7.07 | 9.69 | <0.001 | .17 | 3.42x10^-187^ | 1.14 | 2.23x10^-42^ | |
| vs. | | Leukocyte | 11.35 | 9.49 | 12.82 | <0.001 | .29 | <0.001 | 1.11 | 7.46x10^-39^ | |
| Never smokers | | Lymphocyte | 2.70 | 1.43 | 3.69 | <0.001 | .24 | <0.001 | 1.03 | 8.00x10^-46^ | |
|  | | Lymphocyte % | -0.32 | -0.58 | -0.06 | .02 | .01 | .02 | 0.94 | 2.63x10^-49^ | |
|  | | Monocyte | 2.83 | 2.03 | 3.67 | <0.001 | .16 | 3.55x10^-175^ | 1.05 | 3.91x10^-46^ | |
|  | | Monocyte % | 0.55 | 0.28 | 0.79 | <0.001 | -.05 | 2.53x10^-18^ | 0.97 | 3.64x10^-48^ | |
|  | | Neutrophill | 9.41 | 7.99 | 11.12 | <0.001 | .23 | <0.001 | 1.11 | 5.89x10^-40^ | |
|  | | Neutrophill % | -0.14 | -0.42 | 0.11 | .32 | -.01 | .27 | 1.06 | 4.43x10^-49^ | |
|  | | Platelet | 0.63 | 0.38 | 0.93 | <0.001 | .04 | 6.38x10^-15^ | 1.04 | 1.09x10^-48^ | |
| Data are presented as proportion mediated (%) with 95% confidence interval (CI), β coefficient (from linear regression for exposure→mediator path), and hazard ratio (HR) from Cox model for mediator→outcome path.  Abbreviations: CI, confidence interval; CRP, C‑reactive protein; HR, hazard ratio.  a. Effect of the first half of the mediation pathway(regression analysis); b. Effect of the second half of the mediation pathway. | | | | | | | | | | |  |

| **eTable 7. Mediating effect of inflammatory markers on the relationship between age of smoking initiation and incident anxiety** | | | | | | | | | | |  |
| --- | --- | --- | --- | --- | --- | --- | --- | --- | --- | --- | --- |
| **Smoking initiation age** | **Inflammation**  **marker** | | **Proportion Mediated (%)** | | | | **β ^a^** | ***p* value ^a^** | **HR ^b^** | ***p* value ^b^** | |
|  |  |  | **Value** | **95% CI (Lower)** | **95% CI**  **(Upper)** | ***p* value** |  |  |  |  |  |
| Childhood | | CRP | 7.44 | 6.13 | 8.54 | <0.001 | .34 | <0.001 | 1.07 | 3.28x10^-57^ | |
| vs. | | Leukocyte | 9.89 | 8.36 | 11.67 | <0.001 | .45 | <0.001 | 1.07 | 8.88x10^-53^ | |
| Never smokers | | Lymphocyte | 3.15 | 1.94 | 4.35 | <0.001 | .33 | <0.001 | 1.03 | 2.89x10^-60^ | |
|  | | Lymphocyte % | 0.33 | 0.16 | 0.56 | <0.001 | -.04 | 3.09x10^-8^ | 0.98 | 1.93x10^-64^ | |
|  | | Monocyte | 3.20 | 2.25 | 4.09 | <0.001 | .26 | 2.19x10^-313^ | 1.04 | 1.08x10^-60^ | |
|  | | Monocyte % | 0.28 | 0.06 | 0.52 | .04 | -.07 | 1.08x10^-20^ | 0.99 | 2.61x10^-64^ | |
|  | | Neutrophill | 8.60 | 7.03 | 10.11 | <0.001 | .41 | <0.001 | 1.07 | 8.53x10^-54^ | |
|  | | Neutrophill % | 0.44 | 0.26 | 0.71 | <0.001 | .05 | 2.07x10^-10^ | 1.03 | 2.73x10^-64^ | |
|  | | Platelet | 0.56 | 0.09 | 1.02 | <0.001 | .12 | 1.19x10^-65^ | 1.01 | 2.23x10^-64^ | |
|  | |  |  |  |  |  |  |  |  |  | |
| Adolescence | | CRP | 7.85 | 6.55 | 9.46 | <0.001 | .21 | <0.001 | 1.07 | 6.99x10^-45^ | |
| vs. | | Leukocyte | 11.89 | 9.71 | 14.06 | <0.001 | .29 | <0.001 | 1.07 | 2.23x10^-39^ | |
| Never smokers | | Lymphocyte | 3.55 | 2.35 | 5.08 | <0.001 | .22 | <0.001 | 1.03 | 3.38x10^-46^ | |
|  | | Lymphocyte % | 0.24 | 0.11 | 0.39 | <0.001 | -.01 | .002 | 0.97 | 6.03x10^-50^ | |
|  | | Monocyte | 4.41 | 3.50 | 5.59 | <0.001 | .18 | <.001 | 1.04 | 6.02x10^-46^ | |
|  | | Monocyte % | 0.18 | -0.04 | 0.39 | .10 | -.03 | 3.10x10^-13^ | 0.99 | 1.03x10^-49^ | |
|  | | Neutrophill | 9.66 | 8.11 | 10.98 | <0.001 | .25 | <0.001 | 1.07 | 5.43x10^-41^ | |
|  | | Neutrophill % | 0.25 | 0.09 | 0.43 | <0.001 | .01 | <0.001 | 1.03 | 6.85x10^-50^ | |
|  | | Platelet | 0.53 | 0.11 | 0.94 | .04 | .06 | 6.29x10^-49^ | 1.01 | 9.78x10^-50^ | |
|  | |  |  |  |  |  |  |  |  |  | |
| Adulthood | | CRP | 6.48 | 4.94 | 8.74 | <0.001 | .17 | 3.42x10^-187^ | 1.06 | 7.09x10^-20^ | |
| vs. | | Leukocyte | 11.05 | 8.34 | 14.82 | <0.001 | .29 | <0.001 | 1.06 | 7.55x10^-18^ | |
| Never smokers | | Lymphocyte | 3.38 | 1.49 | 5.24 | <0.001 | .24 | <0.001 | 1.02 | 1.03x10^-20^ | |
|  | | Lymphocyte % | -0.25 | -0.58 | 0.00 | .04 | .01 | .02 | 0.97 | 1.87x10-^22^ | |
|  | | Monocyte | 3.81 | 2.41 | 5.06 | <0.001 | .16 | 3.55x10^-175^ | 1.04 | 8.91x10^-21^ | |
|  | | Monocyte % | 0.23 | -0.09 | 0.81 | .20 | -.05 | 2.53x10^-18^ | 0.99 | 3.88x10^-22^ | |
|  | | Neutrophill | 9.06 | 6.69 | 12.14 | <0.001 | .23 | <0.001 | 1.06 | 1.15x10^-18^ | |
|  | | Neutrophill % | -0.12 | -0.38 | 0.10 | .20 | -.01 | .27 | 1.03 | 2.20x10^-22^ | |
|  | | Platelet | 0.31 | -0.02 | 0.62 | .12 | .04 | 6.38x10^-15^ | 1.01 | 4.79x10^-22^ | |
| Data are presented as proportion mediated (%) with 95% confidence interval (CI), β coefficient (from linear regression for exposure→mediator path), and hazard ratio (HR) from Cox model for mediator→outcome path.  Abbreviations: CI, confidence interval; CRP, C‑reactive protein; HR, hazard ratio.  a. Effect of the first half of the mediation pathway(regression analysis); b. Effect of the second half of the mediation pathway. | | | | | | | | | | |  |

| **eTable 8. Brain regions showing significant associations of structural‑function (SF) coupling with in utero exposure to tobacco** | | | | |
| --- | --- | --- | --- | --- |
| **Brain region** | **Coeff (95% CI)** | | ***t* value** | ***p* value*** |
|  | **Unexposed** | **Exposed** |  |  |
| L_posterior thalamus | 0.134(0.129-0.139) | 0.129(0.124-0.134) | 12.27 | 5.00x10^-4^ |
| L_putamen | 0.104(0.099-0.109) | 0.099(0.094-0.105) | 11.70 | 6.00x10^-4^ |
| L_G_oc_temp_med_Lingual | 0.222(0.218-0.227) | 0.234(0.229-0.239) | 89.67 | <2x10^-16^ |
| L_G_pariet_inf_Angular | 0.180(0.176-0.185) | 0.175(0.170-0.180) | 20.21 | <2x10^-16^ |
| L_S_collat_transv_post | 0.308(0.302-0.313) | 0.318(0.312-0.324) | 38.51 | <2x10^-16^ |
| R_G_oc_temp_med_Lingual | 0.170(0.165-0.175) | 0.179(0.174-0.184) | 45.10 | <2x10^-16^ |
| R_G_Ins_lg_and_S_cent_ins | 0.213(0.208-0.218) | 0.219(0.214-0.224) | 15.92 | 1.00x10^-4^ |
| R_G_and_S_cingul_Mid_Post | 0.188(0.183-0.193) | 0.183(0.177-0.188) | 13.51 | 2.00x10^-4^ |
| R_G_oc_temp_lat_fusifor | 0.270(0.264-0.275) | 0.275(0.269-0.281) | 12.81 | 3.00x10^-4^ |
| R_S_collat_transv_post | 0.252(0.246-0.259) | 0.259(0.252-0.266) | 13.05 | 3.00x10^-4^ |
| L_S_temporal_sup | 0.186(0.182-0.190) | 0.182(0.177-0.186) | 12.65 | 4.00x10^-4^ |
| R_Lat_Fis_post | 0.215(0.209-0.220) | 0.220(0.214-0.226) | 12.00 | 5.00x10^-4^ |
| R_G_and_S_occipital_inf | 0.295(0.289-0.301) | 0.300(0.294-0.307) | 9.93 | 1.60x10^-3^ |
| L_G_and_S_cingul_Mid_Post | 0.181(0.175-0.186) | 0.176(0.170-0.182) | 9.63 | 1.90x10^-3^ |
| R_G_and_S_cingul_Mid_Ant | 0.098(0.093-0.103) | 0.094(0.089-0.099) | 9.41 | 2.20x10^-3^ |
| R_S_precentral_inf_part | 0.264(0.259-0.269) | 0.260(0.255-0.265) | 9.06 | 2.60x10^-3^ |
| L_Pole_occipital | 0.297(0.292-0.302) | 0.301(0.296-0.306) | 8.45 | 3.70x10^-3^ |
| Data are presented as mean (95% confidence interval) of SF coupling. Higher values indicate stronger alignment between structural and functional connectivity.  Abbreviations: CI, confidence interval; Coeff, coefficient; L, left; R, right; SF, structure‑function coupling.  *All p values were two-sided, and no adjustments were made for multiple comparisons. | | | | |

| **eTable 9. Associations of structure-function (SF) coupling in brain regions affected by in utero tobacco exposure with incident depression** | | | | | | | | |
| --- | --- | --- | --- | --- | --- | --- | --- | --- |
| **Brain region** | **Linear association between SF coupling and incident depression** | |  | **Associations between SF coupling tertile and incident depression** | | | | |
|  | **HR (95% CI)** | ***p* value*** |  | **Middle tertile** | |  | **Highest tertile** | |
|  |  |  |  | **HR (95% CI)** | ***p* value*** |  | **HR (95% CI)** | ***p* value*** |
| **L_G_and_S_cingul_Mid_Post** | **1.09 (1.02-1.17)** | **.01** |  | **0.99 (0.83-1.17)** | **.88** |  | **1.23 (1.04-1.45)** | **.01** |
| L_G_oc_temp_med_Lingua | 1.03 (0.96-1.10) | .37 |  | 0.84 (0.71-1.00) | .05 |  | 1.11 (0.95-1.31) | .19 |
| L_G_pariet_inf_Angular | 0.94 (0.88-1.00) | .06 |  | 0.86 (0.73-1.01) | .07 |  | 0.86 (0.73-1.01) | .07 |
| L_Pole_occipital | 1.00 (0.93-1.07) | .91 |  | 0.94 (0.79-1.11) | .46 |  | 1.00 (0.85-1.18) | .97 |
| L_S_collat_transv_post_ | 0.97 (0.91-1.04) | .45 |  | 0.91 (0.77-1.07) | .26 |  | 0.98 (0.83-1.15) | .78 |
| **L_S_temporal_sup** | **0.90 (0.84-0.97)** | **<0.001** |  | **0.87 (0.74-1.02)** | **.08** |  | **0.78 (0.66-0.92)** | **<0.001** |
| **R_G_and_S_occipital_inf** | **1.08 (1.01-1.16)** | **.03** |  | **0.87 (0.73-1.03)** | **.12** |  | **1.17 (1.00-1.37)** | **.06** |
| R_G_and_S_cingul_Mid_Ant | 0.98 (0.92-1.05) | .56 |  | 0.92 (0.78-1.08) | .32 |  | 0.94 (0.80-1.11) | .45 |
| R_G_and_S_cingul_Mid_Post | 1.05 (0.98-1.12) | .16 |  | 1.19 (1.01-1.41) | .04 |  | 1.15 (0.97-1.37) | .10 |
| R_G_Ins_lg_and_S_cent_ins | 1.01 (0.95-1.09) | .69 |  | 1.00 (0.85-1.18) | .98 |  | 1.00 (0.85-1.18) | .98 |
| R_G_oc_temp_lat_fusifor | 1.06 (0.99-1.14) | .09 |  | 1.14 (0.96-1.35) | .12 |  | 1.08 (0.91-1.28) | .36 |
| R_G_oc_temp_med_Lingual | 1.04 (0.97-1.11) | .27 |  | 1.03 (0.88-1.22) | .69 |  | 1.05 (0.89-1.24) | .55 |
| **R_Lat_Fis_post** | **1.10 (1.02-1.17)** | **<0.001** |  | **1.09 (0.92-1.30)** | **.30** |  | **1.26 (1.07-1.49)** | **.01** |
| R_S_collat_transv_post | 0.96 (0.90-1.03) | .23 |  | 1.08 (0.92-1.27) | .36 |  | 0.94 (0.79-1.11) | .46 |
| R_S_precentral_inf_part | 0.99 (0.92-1.06) | .69 |  | 0.94 (0.80-1.11) | .45 |  | 0.93 (0.79-1.10) | .41 |
| L_posterior thalamus | 1.02 (0.95-1.09) | .55 |  | 1.25 (1.05-1.47) | .01 |  | 1.09 (0.92-1.29) | .33 |
| L_putamen | 1.00 (0.93-1.07) | 1.00 |  | 0.94 (0.79-1.11) | .44 |  | 0.99 (0.84-1.16) | .87 |
| Data are presented as hazard ratio (HR) with 95% confidence interval (CI) from Cox proportional hazards models. Tertile analyses used the lowest tertile as the reference.  Abbreviations: CI, confidence interval; HR, hazard ratio; L, left; R, right; SF, structure‑function coupling.  *All p values were two-sided, and no adjustments were made for multiple comparisons. | | | | | | | | |

| **eTable 10. Associations of structure-function (SF) coupling in brain regions affected by in utero tobacco exposure with incident anxiety** | | | | | | | |
| --- | --- | --- | --- | --- | --- | --- | --- |
| **Brain region** | **Linear association between SF coupling and incident anxiety** | |  | **Associations between SF coupling tertile and incident anxiety** | | | |
|  | **HR (95% CI)** | ***p* value*** |  | **Middle tertile** | | **Highest tertile** | |
|  |  |  |  | **HR (95% CI)** | ***p* value*** | **HR (95% CI)** | ***p* value*** |
| L_G_and_S_cingul_Mid_Post | 1.04 (0.97-1.11) | .27 |  | 1.09 (0.92-1.28) | .32 | 1.05 (0.89-1.24) | .54 |
| L_G_oc_temp_med_Lingual | 0.97 (0.91-1.04) | .38 |  | 0.99 (0.85-1.17) | .95 | 1.02 (0.86-1.19) | .86 |
| L_G_pariet_inf_Angular | 0.98 (0.92-1.05) | .60 |  | 0.96 (0.82-1.12) | .60 | 0.92 (0.79-1.09) | .34 |
| L_Pole_occipital | 1.02 (0.96-1.09) | .50 |  | 1.03 (0.87-1.20) | .75 | 0.99 (0.84-1.16) | .91 |
| L_S_collat_transv_post | 0.94 (0.88-1.01) | .08 |  | 0.99 (0.85-1.16) | .93 | 0.91 (0.78-1.07) | .27 |
| L_S_temporal_sup | 0.99 (0.93-1.06) | .82 |  | 1.07 (0.91-1.25) | .41 | 0.97 (0.82-1.14) | .73 |
| R_G_and_S_occipital_inf | 0.96 (0.90-1.03) | .24 |  | 0.92 (0.78-1.08) | .29 | 0.93 (0.80-1.10) | .41 |
| R_G_and_S_cingul_Mid_Ant | 0.98 (0.92-1.05) | .57 |  | 0.93 (0.79-1.09) | .38 | 0.94 (0.80-1.10) | .46 |
| R_G_and_S_cingul_Mid_Post | 1.01 (0.94-1.07) | .87 |  | 1.12 (0.95-1.31) | .17 | 1.01 (0.85-1.19) | .94 |
| R_G_Ins_lg_and_S_cent_ins | 1.05 (0.98-1.12) | .19 |  | 0.99 (0.84-1.16) | .89 | 1.09 (0.93-1.28) | .31 |
| R_G_oc_temp_lat_fusifort | 0.97 (0.91-1.03) | .32 |  | 0.88 (0.75-1.03) | .10 | 0.85 (0.73-1.00) | .05 |
| R_G_oc_temp_med_Lingual | 1.02 (0.96-1.09) | .51 |  | 1.07 (0.92-1.26) | .38 | 1.01 (0.86-1.19) | .92 |
| R_Lat_Fis_postt | 1.04 (0.97-1.11) | .25 |  | 1.04 (0.88-1.23) | .62 | 1.11 (0.94-1.30) | .22 |
| R_S_collat_transv_post | 0.97 (0.90-1.03) | .31 |  | 1.01 (0.86-1.19) | .89 | 0.94 (0.80-1.11) | .48 |
| **R_S_precentral_inf_part** | **0.92 (0.87-0.99)** | **.02** |  | **0.96 (0.82-1.13)** | **.63** | **0.84 (0.71-0.99)** | **.03** |
| L_posterior thalamus | 1.03 (0.96-1.10) | .44 |  | 1.19 (1.01-1.40) | .03 | 1.12 (0.95-1.31) | .19 |
| L_putamen | 0.98 (0.92-1.05) | .64 |  | 1.00 (0.86-1.18) | .95 | 0.96 (0.82-1.13) | .63 |
| Data are presented as hazard ratio (HR) with 95% confidence interval (CI) from Cox proportional hazards models. Tertile analyses used the lowest tertile as the reference.  Abbreviations: CI, confidence interval; HR, hazard ratio; L, left; R, right; SF, structure‑function coupling.  *All p values were two-sided, and no adjustments were made for multiple comparisons. | | | | | | | |

| **eTable 11. Mediating effect of brain structure-function (SF) coupling in critical regions on the relationship between in utero exposure to tobacco and incident depression, anxiety** | | | | | | | | | | |  |  |
| --- | --- | --- | --- | --- | --- | --- | --- | --- | --- | --- | --- | --- |
| **Outcomes** | **Brain region** | **Proportion Mediated (%)** | | | | | **β ^a^** | ***p* value ^a^** | **HR ^b^** | ***p* value ^b^** | |  |
|  |  | **Value** | **95% CI (Lower)** | **95% CI**  **(Upper)** | | ***p* value** |  |  |  |  |  |  |
| Depression | L_G_and_S_cingul_Mid_Post | -1.45 | -5.34 | 0.21 | .08 | | -.04 | 2.74x10^-3^ | 1.52 | 8.58x10^-3^ | |  |
|  | L_S_temporal_sup | 2.79 | 1.02 | 13.17 | <0.001 | | -.04 | 5.79x10^-4^ | 0.43 | .01 | |  |
|  | R_G_and_S_occipital_inf | 1.15 | 0.04 | 4.39 | .04 | | .03 | 8.18x10^-3^ | 1.41 | .01 | |  |
|  | R_Lat_Fis_post | 2.20 | 0.63 | 9.36 | <0.001 | | .05 | 2.86x10^-5^ | 1.57 | .01 | |  |
|  |  |  |  |  |  | |  |  |  |  | |  |
| Anxiety | R_S_precentral_inf_part | 0.20 | -2.03 | 1.61 | .86 | | -.04 | 2.25x10^-3^ | 0.94 | 9.55x10^-3^ | |  |
| Data are presented as proportion mediated (%) with 95% confidence interval (CI), β coefficient (exposure → SF coupling path), and hazard ratio (HR) from Cox model (SF coupling → outcome path).  Abbreviations: CI, confidence interval; HR, hazard ratio; L, left; R, right; SF, structure‑function coupling.  a. Effect of the first half of the mediation pathway; b. Effect of the second half of the mediation pathway. | | | | | | | | | | |  | |
|  | | | | | | | | | | |  | |

| **eTable 12.** **Brain regions showing significant associations of structure-function (SF) coupling with age of smoking initiation** | | | | | | |
| --- | --- | --- | --- | --- | --- | --- |
| **Brain region** | **SF coupling(95% CI)** | | | | ***F* value** | ***p*** **value*** |
|  | **Childhood** | **Adolescence** | **Adulthood** | **Never-smokers** |  |  |
| R_anterior thalamus | 0.134(0.127-0.141) | 0.142(0.137-0.147) | 0.142(0.136-0.148) | 0.147(0.143-0.152) | 9.95 | <2x10^-16^ |
| L_aanterior thalamus | 0.151(0.143-0.159) | 0.161(0.155-0.169) | 0.159(0.152-0.165) | 0.164(0.159-0.169) | 6.11 | 4.00x10^-4^ |
| L_caudate nucleus | 0.200(0.191-0.209) | 0.201(0.195-0.208) | 0.199(0.192-0.207) | 0.210(0.204-0.215) | 10.59 | <2x10^-16^ |
| R_globus pallidus | 0.050(0.042-0.057) | 0.054(0.048-0.060) | 0.053(0.048-0.058) | 0.060(0.056-0.063) | 5.37 | <2x10^-16^ |
| L_G_and_S_transv_frontopol | 0.122(0.111-0.133) | 0.133(0.125-0.141) | 0.130(0.121-0.139) | 0.137(0.130-0.144) | 5.01 | <2x10^-16^ |
| L_G_front_middle | 0.361(0.354-0.367) | 0.364(0.359-0.369) | 0.361(0.355-0.366) | 0.369(0.365-0.373) | 10.05 | <2x10^-16^ |
| L_G_front_sup | 0.246(0.239-0.254) | 0.250(0.245-0.256) | 0.250(0.244-0.256) | 0.254(0.250-0.258) | 4.07 | 0.01 |
| L_G_insular_short | 0.090(0.081-0.098) | 0.086(0.080-0.093) | 0.084(0.077-0.092) | 0.092(0.086-0.098) | 4.60 | <2x10^-16^ |
| L_S_circular_insula_ant | 0.087(0.078-0.097) | 0.080(0.073-0.087) | 0.083(0.075-0.090) | 0.088(0.082-0.094) | 4.97 | <2x10^-16^ |
| L_S_front_inf | 0.338(0.330-0.346) | 0.344(0.338-0.350) | 0.345(0.338-0.351) | 0.347(0.342-0.352) | 3.53 | .01 |
| L_S_front_middle | 0.178(0.166-0.190) | 0.187(0.179-0.196) | 0.186(0.176-0.196) | 0.194(0.186-0.201) | 5.85 | 5.00x10^-4^ |
| L_S_front_sup | 0.214(0.207-0.222) | 0.216(0.211-0.222) | 0.214(0.208-0.221) | 0.222(0.217-0.227) | 7.06 | 1.00x10^-4^ |
| L_S_suborbital | 0.161(0.150-0.172) | 0.165(0.157-0.173) | 0.161(0.152-0.171) | 0.174(0.167-0.181) | 8.98 | <2x10^-16^ |
| L_posterior thalamus | 0.097(0.089-0.104) | 0.101(0.094-0.106) | 0.100(0.094-0.107) | 0.104(0.099-0.109) | 3.24 | .02 |
| R_posterior thalamus | 0.113(0.106-0.121) | 0.113(0.108-0.119) | 0.116(0.109-0.122) | 0.119(0.115-0.124) | 5.20 | <2x10^-16^ |
| R_G_and_S_cingul_Ant | 0.189(0.181-0.197) | 0.186(0.180-0.192) | 0.185(0.178-0.192) | 0.191(0.186-0.196) | 4.52 | <2x10^-16^ |
| R_G_front_middle | 0.335(0.329-0.341) | 0.336(0.331-0.340) | 0.336(0.331-0.341) | 0.342(0.338-0.346) | 10.26 | <2x10^-16^ |
| R_S_front_inf | 0.293(0.285-0.301) | 0.295(0.289-0.301) | 0.297(0.291-0.304) | 0.301(0.296-0.306) | 5.13 | <2x10^-16^ |
| R_S_front_sup | 0.199(0.192-0.207) | 0.199(0.194-0.205) | 0.200(0.194-0.206) | 0.205(0.200-0.209) | 5.03 | <2x10^-1^6 |
| L_putamen | 0.097(0.089-0.104) | 0.101(0.095-0.106) | 0.100(0.094-0.107) | 0.104(0.099-0.109) | 3.35 | .02 |
| L_G_and_S_paracentral | 0.249(0.240-0.257) | 0.258(0.252-0.262) | 0.256(0.249-0.263) | 0.263(0.258-0.268) | 8.43 | <2x10^-16^ |
| L_S_cingul_Marginalis | 0.164(0.156-0.172) | 0.175(0.170-0.181) | 0.169(0.163-0.176) | 0.178(0.173-0.183) | 10.36 | <2x10^-16^ |
| R_G_and_S_paracentral | 0.248(0.239-0.256) | 0.251(0.245-0.257) | 0.248(0.239-0.256) | 0.258(0.253-0.264) | 9.21 | <2x10^-16^ |
| R_G_and_S_cingul_Mid_Ant | 0.092(0.084-0.099) | 0.092(0.086-0.098) | 0.092(0.086-0.098) | 0.099(0.094-0.104) | 8.68 | <2x10^-16^ |
| R_S_precentral_inf_part | 0.260(0.252-0.268) | 0.257(0.252-0.273) | 0.260(0.252-0.268) | 0.265(0.260-0.270) | 7.15 | 1.00x10^-4^ |
| R_S_subparietal | 0.245(0.239-0.252) | 0.245(0.240-0.250) | 0.246(0.240-0.251) | 0.251(0.247-0.255) | 7.35 | 1.00x10^-4^ |
| R_G_precuneus | 0.311(0.305-0.317) | 0.309(0.304-0.313) | 0.308(0.302-0.313) | 0.313(0.310-0.317) | 6.37 | 3.00x10^-4^ |
| L_G_postcentral | 0.263(0.256-0.271) | 0.263(0.258-0.269) | 0.261(0.254-0.267) | 0.268(0.263-0.273) | 5.46 | 9.00x10^-4^ |
| L_S_postcentral | 0.276(0.269-0.282) | 0.279(0.274-0.283) | 0.276(0.270-0.281) | 0.282(0.278-0.286) | 5.34 | 1.10x10^-3^ |
| R_G_and_S_occipital_inf | 0.310(0.300-0.319) | 0.298(0.291-0.304) | 0.298(0.290-0.306) | 0.296(0.289-0.301) | 5.29 | 1.20x10^-3^ |
| R_G_pariet_inf_Angular | 0.188(0.181-0.194) | 0.190(0.185-0.195) | 0.187(0.182-0.193) | 0.193(0.189-0.198) | 5.19 | 1.40x10^-3^ |
| L_S_precentral_sup_part | 0.196(0.188-0.204) | 0.199(0.194-0.205) | 0.200(0.194-0.205) | 0.204(0.200-0.209) | 5.12 | 1.50x10^-3^ |
| R_S_cingul_Marginalis | 0.176(0.169-0.184) | 0.183(0.177-0.188) | 0.176(0.169-0.184) | 0.185(0.180-0.190) | 5.07 | 1.60x10^-3^ |
| R_G_temporal_middle | 0.220(0.212-0.228) | 0.226(0.220-0.232) | 0.220(0.212-0.228) | 0.228(0.223-0.233) | 4.85 | 2.20x10^-3^ |
| R_S_precentral_sup_part | 0.162(0.154-0.169) | 0.163(0.158-0.169) | 0.164(0.158-0.170) | 0.168(0.164-0.173) | 4.48 | 3.80x10^-3^ |
| L_S_central | 0.221(0.214-0.228) | 0.224(0.219-0.229) | 0.224(0.218-0.230) | 0.228(0.224-0.232) | 4.44 | 3.00x10^-3^ |
| R_S_circular_insula_ant | 0.100(0.092-0.109) | 0.104(0.098-0.111) | 0.097(0.090-0.104) | 0.106(0.101-0.111) | 4.37 | 4.40x10^-3^ |
| L_G_precuneus | 0.294(0.289-0.300) | 0.292(0.288-0.297) | 0.294(0.289-0.300) | 0.296(0.292-0.300) | 4.12 | 6.20x10^-3^ |
| R_G_parietal_sup | 0.221(0.214-0.228) | 0.221(0.216-0.226) | 0.221(0.214-0.228) | 0.225(0.221-0.230) | 4.08 | 6.60x10^-3^ |
| R_S_pericallosal | 0.291(0.284-0.298) | 0.283(0.278-0.289) | 0.284(0.278-0.290) | 0.288(0.283-0.292) | 4.05 | 6.90x10^-3^ |
| L_G_temp_sup_G_T_transv | 0.235(0.226-0.244) | 0.225(0.219-0.232) | 0.228(0.220-0.235) | 0.231(0.225-0.237) | 3.87 | 8.90x10^-3^ |
| L_G_Ins_lg_and_S_cent_ins | 0.142(0.133-0.150) | 0.133(0.127-0.139) | 0.128(0.121-0.135) | 0.133(0.127-0.138) | 3.70 | .01 |
| R_S_occipital_ant | 0.222(0212-0.232) | 0.216(0.208-0.219) | 0.211(0.202-0.219) | 0.211(0.204-0.217) | 3.69 | .01 |
| L_G_parietal_sup | 0.197(0.190-0.204) | 0.201(0.197-0.206) | 0.198(0.193-0.204) | 0.203(0.199-0.207) | 3.67 | .01 |
| L_S_oc_middle_and_Lunatus | 0.340(0.333-0.348) | 0.340(0.334-0.345) | 0.343(0.337-0.350) | 0.336(0.332-0.341) | 3.68 | .01 |
| L_G_precentral | 0.276(0.269-0.283) | 0.284(0.279-0.290) | 0.285(0.279-0.291) | 0.286(0.281-0.291) | 3.67 | .01 |
| R_S_intrapariet_and_P_trans | 0.218(0.211-0.224) | 0.220(0.215-0.224) | 0.222(0.216-0.227) | 0.224(0.220-0.228) | 3.65 | .01 |
| L_S_oc_sup_and_transversal | 0.288(0.282-0.294) | 0.285(0.281-0.289) | 0.290(0.285-0.295) | 0.284(0.280-0.288) | 3.64 | .01 |
| R_G_oc_temp_med_Parahip | 0.087(0.078-0.095) | 0.089(0.082-0.095) | 0.089(0.082-0.095) | 0.094(0.088-0.099) | 3.58 | .01 |
| L_G_temp_sup_Lateral | 0.110(0.103-0.117) | 0.120(0.115-0.125) | 0.116(0.110-0.122) | 0.116(0.112-0.121) | 3.57 | .01 |
| Data are presented as mean (95% confidence interval) of SF coupling for each smoking initiation group. ANCOVA was used to compare groups, adjusted for covariates.  Abbreviations: ANCOVA, analysis of covariance; CI, confidence interval; L, left; R, right; SF, structure‑function coupling.  *All p values were two-sided. | | | | | | |

| **eTable 13. Post Hoc p Values for structure-function (SF) coupling by smoking initiation age in ANOVA-significant regions** | | | | | | |
| --- | --- | --- | --- | --- | --- | --- |
| **Brain region** | adolescence vs. adult ^a^ | adolescence vs. childhood | adolescence vs. never_smokers | adult vs. childhood | adult vs. never_smokers | childhood vs. never_smokers |
| R_anterior thalamus |  | 0.026 | 0.002 | 0.031 | 0.031 | 0.000 |
| L_caudate nucleus |  |  | 0.000 |  | 0.001 | 0.015 |
| L_anterior thalamus |  | 0.015 |  |  |  | 0.001 |
| R_globus pallidus |  |  | 0.007 |  |  | 0.018 |
| Rutamen |  |  | 0.003 |  |  |  |
| Lutamen |  |  |  |  |  |  |
| Lutamen |  |  |  |  | 0.019 |  |
| L_G_and_Saracentral |  | 0.016 | 0.016 |  | 0.016 | 0.000 |
| L_G_front_middle |  |  | 0.001 |  | 0.000 | 0.006 |
| L_S_cingul_Marginalis | 0.049 | 0.001 |  |  | 0.001 | 0.000 |
| L_S_suborbital |  |  | 0.001 |  | 0.001 | 0.010 |
| R_G_and_Saracentral |  |  | 0.000 |  | 0.009 | 0.007 |
| R_G_and_S_cingul_Mid_Ant |  |  | 0.000 |  | 0.009 | 0.041 |
| R_G_front_middle |  |  | 0.000 |  | 0.006 | 0.013 |
| L_S_front_sup |  |  | 0.005 |  | 0.005 | 0.041 |
| R_Srecentral_inf |  |  | 0.000 |  |  |  |
| R_S_subparietal |  |  | 0.000 |  | 0.027 |  |
| R_Grecuneus |  |  | 0.003 |  | 0.006 |  |
| L_S_front_middle |  |  | 0.038 |  |  | 0.008 |
| L_Gostcentral |  |  | 0.012 |  | 0.012 |  |
| L_Sostcentral |  |  | 0.050 |  | 0.015 | 0.050 |
| R_G_and_S_occipital |  | 0.009 |  | 0.019 |  | 0.001 |
| R_Gariet_inf_Angular |  |  | 0.032 |  | 0.023 |  |
| L_Srecentral_sup |  |  | 0.018 |  |  | 0.024 |
| R_S_front_inf |  |  | 0.007 |  |  | 0.045 |
| R_S_cingul_Marginalis |  |  |  |  | 0.017 | 0.017 |
| R_S_front_sup |  |  | 0.007 |  |  |  |
| L_G_and_S_transv_frontopol |  |  |  |  |  | 0.006 |
| L_S_circular_insula_ant |  |  | 0.002 |  |  |  |
| R_G_temporal_middle |  |  |  |  | 0.014 | 0.039 |
| L_G_insular_short |  |  | 0.016 |  | 0.016 |  |
| R_G_and_S_cingul_Ant |  |  | 0.013 |  | 0.043 |  |
| R_Srecentral_supart |  |  | 0.018 |  |  |  |
| L_S_central |  |  | 0.041 |  |  | 0.041 |
| R_S_circular_insula_ant | 0.047 |  |  |  | 0.005 |  |
| L_Grecuneus |  |  | 0.029 |  | 0.039 |  |
| R_Garietal_sup |  |  | 0.028 |  |  |  |
| L_G_front_sup |  |  |  |  |  |  |
| R_Sericallosal |  |  | 0.034 |  |  |  |
| L_G_temp_sup_G_T_transv |  | 0.040 | 0.018 |  |  |  |
| L_G_Ins_lg_and_S_cent_ins |  | 0.038 |  | 0.005 |  | 0.022 |
| R_S_occipital_ant |  |  |  |  |  | 0.044 |
| L_Garietal_sup |  |  |  |  |  |  |
| L_S_oc_middle_and_Lunatus |  |  |  |  | 0.027 |  |
| L_Grecentral |  | 0.026 |  | 0.026 |  | 0.007 |
| R_S_intrapariet_and_trans |  |  | 0.043 |  |  |  |
| L_S_oc_sup_and_transversal |  |  |  |  | 0.016 |  |
| R_G_oc_temp_medarahip |  |  | 0.037 |  |  |  |
| L_G_temp_sup_Lateral |  | 0.013 |  |  |  |  |
| L_S_front_inf |  |  |  |  |  | 0.030 |
| Data are presented as p values for post hoc pairwise comparisons (Tukey’s HSD) after ANCOVA. Blank cells indicate corrected p >0.05.  Abbreviations: ANCOVA, analysis of covariance; HSD, honestly significant difference. | | | | | | |
|  |  |  |  |  |  |  |

| **eTable 14. Associations of structure-function (SF) coupling of brain regions affected by age of smoking initiation with incident depression** | | | | | | | | | |
| --- | --- | --- | --- | --- | --- | --- | --- | --- | --- |
| **Brain region** | **Linear association between SF coupling and incident depression** | |  | **Associations between SF coupling tertile and incident depression** | | | | | |
|  | **HR (95% CI)** | ***p* value*** |  | **Middle tertile** | |  | **Highest tertile** | | |
|  |  |  |  | **HR (95% CI)** | ***p* value*** |  | **HR (95% CI)** | ***p* value*** | |
| L_G_and_S_paracentral | 1.00 (0.94-1.07) | .92 |  | 1.04 (0.88-1.23) | .62 |  | 0.93 (0.79-1.10) | .41 |  |
| L_G_and_S_transv_frontopol | 1.00 (0.93-1.07) | .89 |  | 1.11 (0.94-1.31) | .23 |  | 1.01 (0.85-1.19) | .95 |  |
| L_G_front_middle | 0.95 (0.89-1.02) | .16 |  | 1.01 (0.86-1.19) | .90 |  | 0.91 (0.77-1.08) | .29 |  |
| L_G_front_sup | 1.01 (0.94-1.08) | .86 |  | 1.06 (0.89-1.25) | .52 |  | 1.02 (0.86-1.20) | .83 |  |
| L_G_Ins_lg_and_S_cent_ins | 1.03 (0.96-1.10) | .41 |  | 1.09 (0.93-1.29) | .29 |  | 1.08 (0.92-1.28) | .35 |  |
| L_G_insular_short | 1.05 (0.98-1.12) | .18 |  | 0.95 (0.81-1.13) | .59 |  | 1.11 (0.94-1.31) | .22 |  |
| L_G_parietal_sup | 1.04 (0.97-1.11) | .31 |  | 1.09 (0.92-1.29) | .30 |  | 1.05 (0.89-1.24) | .59 |  |
| L_G_postcentral | 0.96 (0.90-1.03) | .26 |  | 0.88 (0.75-1.04) | .14 |  | 0.85 (0.72-1.00) | .06 |  |
| L_G_precentral | 1.01 (0.94-1.08) | .80 |  | 0.97 (0.82-1.14) | .70 |  | 1.01 (0.86-1.19) | .92 |  |
| L_G_precuneus | 1.04 (0.97-1.11) | .31 |  | 1.03 (0.87-1.22) | .74 |  | 1.16 (0.98-1.38) | .08 |  |
| L_G_temp_sup_G_T_transv | 1.02 (0.95-1.09) | .59 |  | 0.87 (0.74-1.03) | .10 |  | 1.00 (0.85-1.18) | 1.00 |  |
| L_G_temp_sup_Lateral | 0.96 (0.90-1.03) | .28 |  | 1.06 (0.90-1.24) | .50 |  | 0.88 (0.75-1.05) | .15 |  |
| L_S_central | 1.04 (0.97-1.12) | .22 |  | 1.04 (0.88-1.23) | .63 |  | 1.12 (0.95-1.33) | .17 |  |
| L_S_cingul_Marginalis | 1.02 (0.95-1.09) | .57 |  | 1.04 (0.88-1.22) | .68 |  | 1.00 (0.85-1.19) | .97 |  |
| L_S_circular_insula_ant | 0.99 (0.92-1.06) | .73 |  | 0.95 (0.81-1.13) | .59 |  | 1.02 (0.86-1.20) | .83 |  |
| L_S_front_inf | 0.97 (0.91-1.04) | .41 |  | 0.97 (0.82-1.14) | .71 |  | 0.93 (0.79-1.10) | .38 |  |
| L_S_front_middle | 0.99 (0.93-1.06) | .82 |  | 0.97 (0.82-1.14) | .69 |  | 1.00 (0.85-1.18) | .97 |  |
| L_S_front_sup | 0.96 (0.89-1.02) | .19 |  | 0.97 (0.83-1.14) | .71 |  | 0.87 (0.73-1.03) | .10 |  |
| L_S_oc_middle_and_Lunatus | 1.06 (0.99-1.13) | .11 |  | 1.06 (0.89-1.25) | .52 |  | 1.14 (0.96-1.34) | .13 |  |
| L_S_oc_sup_and_transversal | 1.09 (1.01-1.16) | .02 |  | 0.95 (0.80-1.12) | .51 |  | 1.10 (0.93-1.29) | .27 |  |
| L_S_postcentral | 0.96 (0.90-1.03) | .24 |  | 1.01 (0.85-1.18) | .95 |  | 0.87 (0.73-1.03) | .10 |  |
| L_S_precentral_sup_part | 0.97 (0.91-1.04) | .35 |  | 1.03 (0.87-1.21) | .74 |  | 0.92 (0.78-1.09) | .32 |  |
| L_S_suborbital | 0.97 (0.91-1.04) | .43 |  | 0.90 (0.76-1.06) | .19 |  | 0.89 (0.75-1.05) | .15 |  |
| R_G_and_S_occipital_inf | 1.08 (1.01-1.16) | .03 |  | 0.87 (0.73-1.03) | .12 |  | 1.17 (1.00-1.37) | .06 |  |
| R_G_and_S_paracentral | 0.97 (0.91-1.04) | .39 |  | 0.92 (0.78-1.08) | .31 |  | 0.86 (0.73-1.01) | .07 |  |
| R_G_and_S_cingul_Ant | 1.08 (1.01-1.16) | .03 |  | 1.33 (1.12-1.58) | <0.001 |  | 1.20 (1.01-1.43) | .04 |  |
| R_G_and_S_cingul_Mid_Ant | 0.98 (0.92-1.05) | .56 |  | 0.92 (0.78-1.08) | .32 |  | 0.94 (0.80-1.11) | .45 |  |
| R_G_front_middle | 0.97 (0.90-1.04) | .35 |  | 0.98 (0.83-1.16) | .83 |  | 0.90 (0.76-1.06) | .22 |  |
| R_G_oc_temp_med_Parahip | 1.00 (0.93-1.07) | .98 |  | 0.90 (0.76-1.06) | .20 |  | 0.99 (0.84-1.17) | .90 |  |
| R_G_pariet_inf_Angular | 0.95 (0.89-1.02) | .16 |  | 0.93 (0.79-1.09) | .36 |  | 0.93 (0.79-1.10) | .39 |  |
| R_G_parietal_sup | 1.03 (0.96-1.10) | .46 |  | 1.07 (0.91-1.27) | .42 |  | 1.05 (0.89-1.24) | .59 |  |
| R_G_precuneus | 1.00 (0.93-1.07) | .97 |  | 1.05 (0.89-1.24) | .56 |  | 1.01 (0.86-1.20) | .87 |  |
| R_G_temporal_middle | 0.98 (0.92-1.05) | .65 |  | 0.99 (0.84-1.16) | .86 |  | 0.94 (0.80-1.11) | .49 |  |
| R_S_cingul_Marginalis | 0.98 (0.92-1.05) | .65 |  | 0.99 (0.84-1.16) | .86 |  | 0.96 (0.82-1.14) | .65 |  |
| R_S_circular_insula_ant | 0.95 (0.89-1.02) | .15 |  | 1.06 (0.90-1.24) | .50 |  | 0.88 (0.74-1.04) | .15 |  |
| R_S_front_inf | 1.01 (0.94-1.08) | .85 |  | 0.91 (0.77-1.07) | .26 |  | 0.98 (0.83-1.16) | .83 |  |
| R_S_front_sup | 0.91 (0.85-0.97) | .01 |  | 0.79 (0.67-0.93) | <0.001 |  | 0.87 (0.74-1.03) | .10 |  |
| R_S_intrapariet_and_P_trans | 1.02 (0.95-1.09) | .61 |  | 1.03 (0.87-1.21) | .77 |  | 1.03 (0.87-1.21) | .77 |  |
| R_S_occipital_ant | 1.06 (0.99-1.14) | .08 |  | 1.01 (0.85-1.19) | .95 |  | 1.24 (1.06-1.47) | .01 |  |
| R_S_pericallosal | 1.02 (0.95-1.09) | .62 |  | 1.13 (0.95-1.34) | .16 |  | 1.10 (0.93-1.31) | .27 |  |
| R_S_precentral_inf_part | 0.99 (0.92-1.06) | .69 |  | 0.94 (0.80-1.11) | .45 |  | 0.93 (0.79-1.10) | .41 |  |
| R_S_precentral_sup_part | 0.96 (0.90-1.03) | .24 |  | 0.84 (0.71-0.99) | .03 |  | 0.88 (0.75-1.04) | .12 |  |
| R_S_subparietal | 1.05 (0.98-1.12) | .21 |  | 1.07 (0.90-1.27) | .43 |  | 1.08 (0.92-1.28) | .34 |  |
| R_posterior thalamus | 0.93 (0.87-0.99) | .03 |  | 0.92 (0.78-1.09) | .33 |  | 0.87 (0.74-1.02) | .09 |  |
| R_anterior thalamus | 0.90 (0.84-0.97) | <0.001 |  | 0.91 (0.77-1.06) | .23 |  | 0.79 (0.67-0.94) | .01 |  |
| R_globus pallidus | 0.97 (0.91-1.04) | .40 |  | 0.94 (0.80-1.10) | .43 |  | 0.89 (0.76-1.05) | .18 |  |
| L_posterior thalamus | 1.02 (0.95-1.09) | .55 |  | 1.25 (1.05-1.47) | .01 |  | 1.09 (0.92-1.29) | .33 |  |
| L_anterior thalamus | 0.94 (0.88-1.01) | .07 |  | 0.88 (0.75-1.04) | .14 |  | 0.85 (0.72-1.00) | .05 |  |
| L_putamen | 1.00 (0.93-1.07) | 1.00 |  | 0.94 (0.79-1.11) | .44 |  | 0.99 (0.84-1.16) | .87 |  |
| L_caudate nucleus | 0.94 (0.88-1.00) | .06 |  | 0.99 (0.84-1.17) | .93 |  | 0.91 (0.77-1.07) | .26 |  |
| Data are presented as hazard ratio (HR) with 95% confidence interval (CI) from Cox proportional hazards models. Tertile analyses used the lowest tertile as the reference.  Abbreviations: CI, confidence interval; HR, hazard ratio; L, left; R, right; SF, structure‑function coupling.  *All p values were two-sided, and no adjustments were made for multiple comparisons. | | | | | | | | |  |

| **eTable 15. Associations of structure-function (SF) coupling of brain regions affected by age of smoking initiation with incident anxiety** | | | | | | | | |
| --- | --- | --- | --- | --- | --- | --- | --- | --- |
| **Brain region** | **Linear association between SF coupling and incident anxiety** | |  | **Associations between SF coupling tertile and incident anxiety** | | | | |
|  | **HR (95% CI)** | ***p* value*** |  | **Middle tertile** | |  | **Highest tertile** | |
|  |  |  |  | **HR (95% CI)** | ***p* value*** |  | **HR (95% CI)** | ***p* value*** |
| L_G_and_S_paracentral | 1.00 (0.94-1.07) | .93 |  | 0.97 (0.83-1.14) | .71 |  | 0.94 (0.80-1.11) | .46 |
| L_G_and_S_transv_frontopol | 0.94 (0.88-1.01) | .07 |  | 0.99 (0.84-1.16) | .86 |  | 0.88 (0.74-1.03) | .11 |
| L_G_front_middle | 0.97 (0.91-1.04) | .36 |  | 0.95 (0.81-1.12) | .54 |  | 1.00 (0.85-1.17) | .98 |
| L_G_front_sup | 1.00 (0.94-1.07) | .97 |  | 0.99 (0.84-1.16) | .86 |  | 1.04 (0.89-1.22) | .64 |
| L_G_Ins_lg_and_S_cent_ins | 1.03 (0.97-1.10) | .34 |  | 0.94 (0.80-1.11) | .47 |  | 1.05 (0.90-1.24) | .51 |
| L_G_insular_short | 1.00 (0.93-1.06) | .89 |  | 0.99 (0.84-1.16) | .86 |  | 0.99 (0.85-1.17) | .94 |
| L_G_parietal_sup | 0.98 (0.92-1.05) | .57 |  | 1.06 (0.90-1.24) | .49 |  | 0.97 (0.82-1.14) | .72 |
| L_G_postcentral | 1.02 (0.95-1.09) | .55 |  | 1.06 (0.91-1.25) | .45 |  | 0.95 (0.81-1.12) | .57 |
| L_G_precentral | 1.04 (0.97-1.11) | .29 |  | 1.00 (0.85-1.18) | 1.00 |  | 0.99 (0.84-1.16) | .92 |
| L_G_precuneus | 0.98 (0.91-1.05) | .50 |  | 0.97 (0.82-1.14) | .69 |  | 1.02 (0.87-1.20) | .80 |
| L_G_temp_sup_G_T_transv | 1.03 (0.96-1.10) | .39 |  | 0.94 (0.80-1.11) | .47 |  | 1.00 (0.85-1.17) | 1.00 |
| L_G_temp_sup_Lateral | 1.04 (0.97-1.11) | .25 |  | 1.16 (0.99-1.37) | .07 |  | 1.08 (0.92-1.27) | .37 |
| L_S_central | 1.09 (1.02-1.17) | .01 |  | 1.15 (0.98-1.35) | .10 |  | 1.15 (0.98-1.36) | .09 |
| L_S_cingul_Marginalis | 0.99 (0.93-1.06) | .87 |  | 0.98 (0.84-1.16) | .84 |  | 1.01 (0.86-1.19) | .86 |
| L_S_circular_insula_ant | 0.99 (0.92-1.05) | .68 |  | 1.04 (0.88-1.22) | .67 |  | 1.06 (0.90-1.25) | .46 |
| L_S_front_inf | 1.00 (0.94-1.07) | .91 |  | 0.92 (0.78-1.08) | .29 |  | 0.94 (0.80-1.10) | .45 |
| L_S_front_middle | 0.96 (0.90-1.02) | .23 |  | 1.05 (0.89-1.23) | .56 |  | 0.91 (0.78-1.08) | .28 |
| L_S_front_sup | 0.99 (0.92-1.05) | .68 |  | 0.92 (0.78-1.08) | .29 |  | 1.00 (0.86-1.18) | .97 |
| L_S_oc_middle_and_Lunatus | 1.03 (0.96-1.10) | .38 |  | 1.05 (0.90-1.24) | .52 |  | 1.07 (0.91-1.26) | .38 |
| L_S_oc_sup_and_transversal | 1.03 (0.97-1.11) | .33 |  | 0.94 (0.80-1.11) | .48 |  | 1.00 (0.85-1.17) | .98 |
| L_S_postcentral | 0.96 (0.90-1.03) | .24 |  | 1.05 (0.89-1.23) | .58 |  | 0.90 (0.77-1.06) | .22 |
| L_S_precentral_sup_part | 0.99 (0.93-1.06) | .82 |  | 1.00 (0.85-1.17) | .98 |  | 0.96 (0.82-1.13) | .65 |
| L_S_suborbital | 1.00 (0.94-1.07) | .98 |  | 0.91 (0.77-1.07) | .26 |  | 1.02 (0.87-1.19) | .85 |
| R_G_and_S_occipital_inf | 0.96 (0.90-1.03) | .24 |  | 0.92 (0.78-1.08) | .29 |  | 0.93 (0.80-1.10) | .41 |
| R_G_and_S_paracentral | 0.95 (0.89-1.01) | .11 |  | 0.94 (0.80-1.11) | .48 |  | 0.92 (0.79-1.08) | .33 |
| R_G_and_S_cingul_Ant | 1.02 (0.95-1.09) | .59 |  | 0.99 (0.84-1.16) | .89 |  | 1.05 (0.89-1.24) | .54 |
| R_G_and_S_cingul_Mid_Ant | 0.98 (0.92-1.05) | .57 |  | 0.93 (0.79-1.09) | .38 |  | 0.94 (0.80-1.10) | .46 |
| R_G_front_middle | 0.93 (0.87-0.99) | .02 |  | 0.96 (0.82-1.12) | .60 |  | 0.83 (0.71-0.98) | .03 |
| R_G_oc_temp_med_Parahip | 0.98 (0.92-1.05) | .56 |  | 1.00 (0.85-1.17) | .98 |  | 0.96 (0.81-1.13) | .60 |
| R_G_pariet_inf_Angular | 1.00 (0.94-1.07) | .98 |  | 1.00 (0.85-1.18) | 1.00 |  | 1.08 (0.92-1.27) | .35 |
| R_G_parietal_sup | 1.01 (0.95-1.08) | .71 |  | 1.11 (0.95-1.31) | .19 |  | 1.03 (0.88-1.22) | .71 |
| R_G_precuneus | 0.96 (0.90-1.02) | .22 |  | 1.18 (1.01-1.38) | .04 |  | 0.91 (0.77-1.08) | .27 |
| R_G_temporal_middle | 1.01 (0.94-1.08) | .78 |  | 0.99 (0.84-1.16) | .86 |  | 1.00 (0.85-1.18) | .99 |
| R_S_cingul_Marginalis | 0.97 (0.91-1.04) | .42 |  | 0.93 (0.79-1.10) | .40 |  | 0.94 (0.80-1.11) | .47 |
| R_S_circular_insula_ant | 0.95 (0.89-1.01) | .09 |  | 0.94 (0.80-1.10) | .45 |  | 0.84 (0.72-0.99) | .04 |
| R_S_front_inf | 1.00 (0.93-1.07) | .96 |  | 0.91 (0.78-1.08) | .28 |  | 1.02 (0.87-1.20) | .78 |
| R_S_front_sup | 0.90 (0.84-0.96) | <0.001 |  | 0.81 (0.69-0.95) | .01 |  | 0.81 (0.69-0.94) | .01 |
| R_S_intrapariet_and_P_trans | 0.94 (0.88-1.00) | .05 |  | 0.95 (0.81-1.11) | .51 |  | 0.90 (0.77-1.06) | .22 |
| R_S_occipital_ant | 1.04 (0.97-1.11) | .24 |  | 1.01 (0.86-1.19) | .86 |  | 1.07 (0.91-1.25) | .42 |
| R_S_pericallosal | 0.97 (0.91-1.03) | .34 |  | 0.93 (0.79-1.10) | .40 |  | 0.92 (0.78-1.08) | .31 |
| R_S_precentral_inf_part | 0.92 (0.87-0.99) | .02 |  | 0.96 (0.82-1.13) | .63 |  | 0.84 (0.71-0.99) | .03 |
| R_S_precentral_sup_part | 0.92 (0.86-0.98) | .01 |  | 0.90 (0.77-1.05) | .19 |  | 0.84 (0.72-0.99) | .04 |
| R_S_subparietal | 0.97 (0.91-1.04) | .37 |  | 1.00 (0.85-1.17) | 1.00 |  | 0.95 (0.81-1.12) | .52 |
| R_posterior thalamus | 0.99 (0.93-1.06) | .86 |  | 1.09 (0.92-1.28) | .32 |  | 1.07 (0.91-1.25) | .44 |
| R_anterior thalamus | 0.94 (0.88-1.01) | .09 |  | 1.00 (0.85-1.17) | .97 |  | 0.95 (0.81-1.12) | .56 |
| R_globus pallidus | 0.96 (0.90-1.03) | .28 |  | 0.88 (0.75-1.03) | .12 |  | 0.90 (0.77-1.05) | .18 |
| L_posterior thalamus | 1.03 (0.96-1.10) | .44 |  | 1.19 (1.01-1.40) | .03 |  | 1.12 (0.95-1.31) | .19 |
| L_anterior thalamus | 0.98 (0.92-1.05) | .65 |  | 0.94 (0.80-1.10) | .45 |  | 0.95 (0.81-1.12) | .57 |
| L_putamen | 0.98 (0.92-1.05) | .64 |  | 1.00 (0.86-1.18) | .95 |  | 0.96 (0.82-1.13) | .63 |
| L_caudate nucleus | 0.96 (0.90-1.03) | .25 |  | 0.99 (0.85-1.16) | .93 |  | 0.90 (0.77-1.06) | .22 |
| Data are presented as hazard ratio (HR) with 95% confidence interval (CI) from Cox proportional hazards models. Tertile analyses used the lowest tertile as the reference.  Abbreviations: CI, confidence interval; HR, hazard ratio; L, left; R, right; SF, structure‑function coupling.  *All p values were two-sided, and no adjustments were made for multiple comparisons. | | | | | | | | |

| **eTable 16. Mediating effect of brain structure-function (SF) coupling in critical regions on the relationship between age of smoking initiation and incident depression** | | | | | | | | | |  |
| --- | --- | --- | --- | --- | --- | --- | --- | --- | --- | --- |
| **Smoking initiation age** | **Brain region mediator** | **Proportion Mediated (%)** | | | | **β ^a^** | ***p* value ^a^** | **HR ^b^** | ***p* value ^b^** |  |
|  |  | **Value** | **95% CI (Lower)** | **95% CI**  **(Upper)** | ***p* value** |  |  |  |  |  |
| Childhood | L_S_oc_sup_and_transversal | 0.77 | -0.15 | 2.30 | .10 | .05 | .09 | 2.31 | 7.45x10^-6^ |  |
| vs. | R_G_and_S_occipital_inf | 0.96 | -0.29 | 2.71 | .20 | .11 | 2.15x10^-4^ | 1.35 | 8.30x10^-6^ |  |
| Never smokers | R_G_and_S_cingul_Ant | -0.18 | -1.55 | 0.64 | .50 | -.01 | .63 | 1.62 | 6.81x10^-6^ |  |
|  | R_S_front_sup | 0.64 | -0.12 | 2.71 | .14 | -.05 | .07 | 0.60 | 8.38x10^-6^ |  |
|  | R_posterior thalamus | 0.81 | -0.12 | 3.20 | .22 | -.06 | .04 | 0.57 | 8.46x10^-6^ |  |
|  | R_anterior thalamus | 2.43 | 0.86 | 7.19 | .02 | -.13 | 8.30x10^-6^ | 0.44 | 1.22x10^-5^ |  |
|  |  |  |  |  |  |  |  |  |  |  |
| Adolescence | L_S_oc_sup_and_transversal | 0.46 | -0.11 | 1.94 | .30 | .02 | .14 | 1.82 | 5.69x10^-5^ |  |
| vs. | R_G_and_S_occipital_inf | 0.55 | -0.18 | 1.64 | .18 | .02 | .10 | 1.50 | 5.80x10^-5^ |  |
| Never smokers | R_G_and_S_cingul_Ant | -0.73 | -2.14 | 0.34 | .20 | -.05 | 2.32x10^-3^ | 1.40 | 4.83x10^-5^ |  |
|  | R_S_front_sup | 1.44 | 0.21 | 3.61 | .02 | -.06 | 2.92x10^-4^ | 0.54 | 6.97x10^-5^ |  |
|  | R_posterior thalamus | 1.55 | 0.21 | 3.17 | .02 | -.05 | 6.83x10^-4^ | 0.51 | 7.16x10^-5^ |  |
|  | R_anterior thalamus | 1.60 | 0.30 | 4.59 | .02 | -.05 | 6.93x10^-4^ | 0.47 | 6.99x10^-5^ |  |
|  |  |  |  |  |  |  |  |  |  |  |
| Adulthood | L_S_oc_sup_and_transversal | 1.72 | -3.00 | 18.00 | .12 | .07 | 1.91x10^-3^ | 1.81 | .03 |  |
| vs. | R_G_and_S_occipital_inf | 0.84 | -1.53 | 7.94 | .38 | .03 | .18 | 1.52 | .03 |  |
| Never smokers | R_G_and_S_cingul_Ant | -0.89 | -5.61 | 1.07 | .36 | -.05 | .02 | 1.37 | .02 |  |
|  | R_S_front_sup | 1.68 | -0.74 | 7.89 | .10 | -.05 | .02 | 0.54 | .03 |  |
|  | R_posterior thalamus | 1.41 | -0.04 | 5.81 | .06 | -.04 | .07 | 0.52 | .03 |  |
|  | R_anterior thalamus | 1.82 | -11.46 | 7.08 | .08 | -.05 | .02 | 0.51 | .03 |  |
| Data are presented as proportion mediated (%) with 95% confidence interval (CI), β coefficient (exposure → SF coupling path), and hazard ratio (HR) from Cox model (SF coupling → outcome path).  Abbreviations: CI, confidence interval; HR, hazard ratio; L, left; R, right; SF, structure‑function coupling.  a. Effect of the first half of the mediation pathway; b. Effect of the second half of the mediation pathway. | | | | | | | | | |  |
|  | | | | | | | | | |  |

| **eTable 17. Mediating effect of critical structure-function (SF) coupling markers on the relationship between age of smoking initiation and incident anxiety** | | | | | | | | | | |
| --- | --- | --- | --- | --- | --- | --- | --- | --- | --- | --- |
| **Smoking initiation age** | **Brain region mediator** | **Proportion Mediated (%)** | | | | **β ^a^** | ***p* value ^a^** | **HR ^b^** | | ***p* value ^b^** |
|  |  | **Value** | **95% CI (Lower)** | **95% CI**  **(Upper)** | ***p* value** |  |  |  |  |  |
| Childhood | L_S_central | -1.67 | -17.86 | 6.92 | .26 | -.07 | .02 | 1.41 | | .13 |
| vs. | R_G_front_middle | 2.69 | -9.71 | 47.83 | .16 | -.08 | 3.32x10^-3^ | 0.59 | | .15 |
| Never smokers | R_S_front_sup | 2.19 | -44.23 | 20.78 | .26 | -.05 | .07 | 0.57 | | .15 |
|  | R_S_precentral_inf_part | 3.36 | -9.88 | 27.40 | .20 | -.07 | .02 | 0.52 | | .16 |
|  | R_S_precentral_sup_part | 2.04 | -12.36 | 15.49 | .20 | -.08 | 5.51x10^-3^ | 0.70 | | .15 |
|  |  |  |  |  |  |  |  |  | |  |
| Adolescence | L_S_central | -1.25 | -4.44 | -0.08 | .04 | -.05 | 2.65x10^-3^ | 1.60 | | 2.94x10^-4^ |
| vs. | R_G_front_middle | 1.22 | -0.70 | 3.78 | .28 | -.08 | 2.75x10^-7^ | 0.73 | | 4.11x10^-4^ |
| Never smokers | R_S_front_sup | 1.83 | 0.40 | 5.30 | <0.001 | -.06 | 2.92x10^-4^ | 0.58 | | 4.51x10^-4^ |
|  | R_S_precentral_inf_part | 2.39 | 0.32 | 7.01 | .04 | -.09 | 1.59x10^-8^ | 0.64 | | 4.92x10^-4^ |
|  | R_S_precentral_sup_part | 1.30 | 0.11 | 3.35 | .02 | -.06 | 2.86x10^-4^ | 0.68 | | 4.12x10^-4^ |
|  |  |  |  |  |  |  |  |  | |  |
| Adulthood | L_S_central | -1.00 | -10.19 | 0.45 | .24 | -.05 | .03 | 1.41 | | .02 |
| vs. | R_G_front_middle | 0.93 | -2.52 | 5.98 | .38 | -.07 | 7.59x10^-4^ | 0.78 | | .02 |
| Never smokers | R_S_front_sup | 1.94 | -2.19 | 14.17 | .06 | -.05 | .02 | 0.55 | | .02 |
|  | R_S_precentral_inf_part | 1.32 | -0.08 | 9.34 | .08 | -.04 | .05 | 0.62 | | .02 |
|  | R_S_precentral_sup_part | 1.03 | -0.47 | 5.40 | .06 | -.05 | .02 | 0.73 | | .02 |
| Data are presented as proportion mediated (%) with 95% confidence interval (CI), β coefficient (exposure → SF coupling path), and hazard ratio (HR) from Cox model (SF coupling → outcome path).  Abbreviations: CI, confidence interval; HR, hazard ratio; L, left; R, right; SF, structure‑function coupling.  a. Effect of the first half of the mediation pathway; b. Effect of the second half of the mediation pathway. | | | | | | | | |  | |

| **eTable 18. Significant associations between inflammatory markers and structure-function (SF) coupling in regions affected by in utero tobacco exposure** | | | | |
| --- | --- | --- | --- | --- |
| **Inflammation marker** | **Brain region** | ***b* value** | ***p* value** | ***p*_-FDR*_** |
| CRP | L_G_pariet_inf_Angular | -0.02 | <2x10^-16^ | <2x10^-16^ |
|  | R_G_oc_temp_med_Lingual | 0.02 | <2x10^-16^ | <2x10^-16^ |
|  | L_S_temporal_sup | -0.03 | <2x10^-16^ | <2x10^-16^ |
|  | R_S_precentral_inf_part | -0.02 | 5.00x10^-4^ | 2.13x10^-3^ |
|  | L_G_oc_temp_med_Lingual | 0.02 | 8.00x10^-4^ | 2.72x10^-3^ |
|  | R_S_collat_transv_post | -0.01 | 7.60x10^-3^ | .02 |
|  | R_Lat_Fis_post | 0.01 | .01 | .03 |
|  | R_G_and_S_cingul_Mid_Post | 0.01 | .02 | .04 |
|  |  |  |  |  |
| Leukocyte | R_Lat_Fis_post | 0.02 | 2.00x10^-4^ | 3.40x10^-3^ |
|  | L_G_oc_temp_med_Lingual | 0.02 | 5.00x10^-4^ | 4.25x10^-3^ |
|  | L_S_temporal_sup | -0.02 | 1.90x10^-3^ | .01 |
|  | R_G_oc_temp_lat_fusifor | 0.02 | 2.40x10^-3^ | .01 |
|  | R_G_and_S_occipital_inf | 0.02 | 3.80x10^-3^ | .01 |
|  |  |  |  |  |
| Lymphocyte | R_S_precentral_inf_part | -0.02 | 1.20x10^-3^ | .02 |
|  | L_S_temporal_sup | -0.02 | 2.50x10^-3^ | .02 |
|  | R_G_and_S_cingul_Mid_Ant | 0.01 | 6.80x10^-3^ | .04 |
|  |  |  |  |  |
| Lymphocyte % | R_S_precentral_inf_part | -0.02 | 2.80x10^-3^ | .05 |
|  |  |  |  |  |
| Monocyte | R_Lat_Fis_post | 0.03 | <2x10^-16^ | <2x10^-16^ |
|  |  |  |  |  |
| Neutrophill | R_Lat_Fis_post | 0.02 | 6.00x10^-4^ | .01 |
|  | L_G_oc_temp_med_Lingual | 0.02 | 3.10x10^-3^ | .03 |
| Data are presented as unstandardized regression coefficient (b) with 95% confidence interval (CI). Linear regression models adjusted for covariates. FDR correction was applied across brain regions.  Abbreviations: CI, confidence interval; CRP, C‑reactive protein; FDR, false discovery rate; L, left; R, right; SF, structure‑function coupling.  *All p values were two-sided. The *p*_-FDR_ values were calculated using the Benjamini-Hochberg FDR method. | | | | |

| **eTable 19.** **Mediating effect of inflammation markers on the relationship between in utero exposure to tobacco and structure-function (SF) coupling in critical regions** | | | | | | | | | | |  |  |
| --- | --- | --- | --- | --- | --- | --- | --- | --- | --- | --- | --- | --- |
| **Inflammation mediator** | **Brain region** | **Proportion Mediated (%)** | | | | | **β ^a^** | ***p* value ^a^** | **β ^b^** | ***p* value ^b^** | |  |
|  |  | **Value** | **95 CI (Lower)** | **95 CI**  **(Upper)** | | ***p* value** |  |  |  |  |  |  |
| CRP | L_G_pariet_inf_Angular | 2.05 | 0.75 | 5.13 | <0.001 | | .05 | 3.95x10^-5^ | -.020 | 2.72x10^-4^ | |  |
|  | R_G_oc_temp_med_Lingual | 1.22 | 0.43 | 2.56 | <0.001 | | .05 | 3.95x10^-5^ | .019 | 6.67x10^-4^ | |  |
|  | L_S_temporal_sup | 2.80 | 0.97 | 9.44 | 5.00x10^-3^ | | .05 | 3.95x10^-5^ | -.021 | 1.98x10^-4^ | |  |
|  | R_S_precentral_inf_part | 2.99 | 0.90 | 12.75 | .01 | | .05 | 3.95x10^-5^ | -.019 | 6.65x10^-4^ | |  |
|  | L_G_oc_temp_med_Lingual | 0.70 | 0.21 | 1.46 | 3.00x10^-3^ | | .05 | 3.95x10^-5^ | .016 | 3.81x10^-3^ | |  |
|  | R_S_collat_transv_post | -1.87 | -8.32 | -0.17 | .03 | | .05 | 3.95x10^-5^ | -.012 | .03 | |  |
|  |  |  |  |  |  | |  |  |  |  | |  |
| Leukocyte | R_Lat_Fis_post | 1.17 | 0.19 | 3.30 | .02 | | 7.24x10^-3^ | .02 | .085 | 2.30x10^-4^ | |  |
|  | L_G_oc_temp_med_Lingual | 0.41 | 0.05 | 1.03 | .02 | | 7.24x10^-3^ | .02 | .067 | 3.56x10^-3^ | |  |
|  | L_S_temporal_sup | 1.34 | 0.18 | 4.30 | .02 | | 7.24x10^-3^ | .02 | -.080 | 5.00x10^-4^ | |  |
|  | R_G_oc_temp_lat_fusifor | 1.10 | 0.09 | 3.96 | .02 | | 7.24x10^-3^ | .02 | .063 | 6.47x10^-3^ | |  |
|  | R_G_and_S_occipital_inf | 1.10 | 0.01 | 5.59 | .05 | | 7.24x10^-3^ | .02 | .051 | .03 | |  |
|  |  |  |  |  |  | |  |  |  |  | |  |
| Lymphocyte | R_S_precentral_inf_part | 2.11 | 0.46 | 8.93 | .02 | | 9.00x10^-3^ | 2.04x10^-4^ | -.081 | 4.92x10^-3^ | |  |
|  | L_S_temporal_sup | 1.50 | 0.25 | 4.64 | .01 | | 9.00x10^-3^ | 2.04x10^-4^ | -.073 | .01 | |  |
|  | R_G_and_S_cingul_Mid_Ant | -1.45 | -4.64 | -0.23 | .02 | | 9.00x10^-3^ | 2.04x10^-4^ | .070 | .02 | |  |
|  |  |  |  |  |  | |  |  |  |  | |  |
| Monocyte | R_Lat_Fis_post | 1.46 | 0.32 | 3.86 | 9.00x10^-3^ | | 3.31x10^-3^ | 9.85x10^-3^ | .231 | 2.21x10^-5^ | |  |
| Data are presented as proportion mediated (%) with 95% confidence interval (CI), β coefficients for exposure→inflammation and inflammation→SF coupling paths.  Abbreviations: CI, confidence interval; CRP, C‑reactive protein; SF, structure‑function coupling.  a. Effect of the first half of the mediation pathway; b. Effect of the second half of the mediation pathway. | | | | | | | | | | |  | |
|  | | | | | | | | | | |  | |

| **eTable 20. Significant associations between inflammatory markers and structure-function (SF) coupling in regions affected by age of smoking initiation** | | | | |
| --- | --- | --- | --- | --- |
| **Inflammation marker** | **Brain region** | ***b* value** | ***p* value** | ***p*_-FDR*_** |
| CRP | R_G_front_middle | -0.02 | <2x10^-16^ | <2x10^-16^ |
|  | R_G_pariet_inf_Angular | -0.03 | <2x10^-16^ | <2x10^-16^ |
|  | L_S_precentral_sup_part | -0.02 | <2x10^-16^ | <2x10^-16^ |
|  | R_S_front_inf | -0.03 | <2x10^-16^ | <2x10^-16^ |
|  | L_S_front_inf | -0.02 | <2x10^-16^ | <2x10^-16^ |
|  | R_S_precentral_sup_part | -0.02 | 1.00x10^-4^ | 5.56x10^-4^ |
|  | R_S_occipital_ant | 0.02 | 1.00x10^-4^ | 5.56x10^-4^ |
|  | L_G_precentral | -0.02 | 1.00x10^-4^ | 5.56x10^-4^ |
|  | L_G_temp_sup_Lateral | -0.02 | 1.00x10^-4^ | 5.56x10^-4^ |
|  | R_S_front_sup | -0.02 | 4.00x10^-4^ | 2.00x10^-3^ |
|  | R_S_precentral_inf_part | -0.02 | 5.00x10^-4^ | 2.27x10^-3^ |
|  | L_S_front_sup | -0.02 | 1.50x10^-3^ | 6.15x10^-3^ |
|  | L_S_oc_sup_and_transversal | 0.02 | 1.60x10^-3^ | 6.15x10^-3^ |
|  | R_G_and_S_cingul_Ant | 0.02 | 2.20x10^-3^ | 7.86x10^-3^ |
|  | R_S_subparietal | 0.02 | 4.30x10^-3^ | .01 |
|  | L_G_front_middle | -0.01 | .01 | .04 |
|  | L_S_suborbital | -0.01 | .01 | .04 |
|  |  |  |  |  |
| Leukocyte | L_S_precentral_sup_part | -0.02 | 4.00x10^-4^ | .01 |
|  | R_S_precentral_sup_part | -0.02 | 4.00x10^-4^ | .01 |
|  | R_G_pariet_inf_Angular | -0.02 | 1.60x10^-3^ | .02 |
|  | R_G_and_S_paracentral | -0.02 | 1.90x10^-3^ | .02 |
|  | R_G_and_S_occipital_inf | 0.02 | 3.80x10^-3^ | .03 |
|  | L_caudate nucleus | -0.01 | 4.70x10^-3^ | .03 |
|  | R_G_front_middle | -0.01 | 4.70x10^-3^ | .03 |
|  | L_S_oc_middle_and_Lunatus | 0.01 | 5.20x10-^3^ | .03 |
|  |  |  |  |  |
| Lymphocyte | R_S_front_inf | -0.02 | <2x10^-16^ | <2x10^-16^ |
|  | R_G_front_middle | -0.02 | 5.00x10^-4^ | .01 |
|  | R_S_precentral_inf_part | -0.02 | 1.20x10^-3^ | .01 |
|  | L_S_precentral_sup_part | -0.02 | 1.30x10^-3^ | .01 |
|  | R_S_precentral_sup_part | -0.02 | 1.40x10^-3^ | .01 |
|  | R_G_pariet_inf_Angular | -0.02 | 1.70x10^-3^ | .01 |
|  | R_G_and_S_cingul_Mid_Ant | 0.01 | 6.80x10^-3^ | .05 |
|  |  |  |  |  |
| Lymphocyte % | L_G_temp_sup_G_T_transv | 0.02 | 6.00x10^-4^ | .03 |
|  |  |  |  |  |
| Monocyte | R_S_precentral_sup_part | -0.02 | 1.10x10^-3^ | .03 |
|  | L_S_oc_sup_and_transversal | 0.02 | 1.30x10^-3^ | .03 |
|  | L_caudate nucleus | -0.02 | 1.50x10^-3^ | .03 |
|  |  |  |  |  |
| Neutrophill % | L_G_temp_sup_G_T_transv | -0.02 | 9.00x10^-4^ | .05 |
|  |  |  |  |  |
| Platelet | R_S_front_sup | -0.02 | 8.00x10^-4^ | .02 |
|  | R_G_pariet_inf_Angular | -0.02 | 1.30x10^-3^ | .02 |
|  | L_G_temp_sup_G_T_transv | -0.02 | 1.40x10^-3^ | .02 |
| Data are presented as unstandardized regression coefficient (b) with 95% confidence interval (CI). Linear regression models adjusted for covariates. FDR correction was applied across brain regions.  Abbreviations: CI, confidence interval; CRP, C‑reactive protein; FDR, false discovery rate; L, left; R, right; SF, structure‑function coupling.  *All p values were two-sided. The *p*_-FDR_ values were calculated using the Benjamini-Hochberg FDR method. | | | | |

| **eTable 21.** **Mediating effect of inflammatory markers on the relationship between childhood smoking initiation and structure-function (SF) coupling in critical regions** | | | | | | | | | |  |
| --- | --- | --- | --- | --- | --- | --- | --- | --- | --- | --- |
| **Inflammation mediator** | **Brain region** | **Proportion Mediated (%)** | | | | **β ^a^** | ***p* value ^a^** | **β ^b^** | ***p* value ^b^** | |
|  |  | **Value** | **95 CI (Lower)** | **95 CI**  **(Upper)** | ***p* value** |  |  |  |  |  |
| CRP | R_G_front_middle | 7.60 | 2.46 | 30.61 | 9.00x10^-3^ | .30 | 2.96x10^-23^ | -.021 | 1.52x10^-3^ | |
|  | R_G_pariet_inf_Angular | 9.97 | 2.60 | 49.31 | .03 | .30 | 2.96x10^-23^ | -.023 | 4.33x10^-4^ | |
|  | L_S_precentral_sup_part | 9.26 | 3.84 | 32.48 | 9.00x10^-3^ | .30 | 2.96x10^-23^ | -.027 | 5.71x10^-5^ | |
|  | R_S_front_inf | 9.52 | 4.07 | 33.28 | 9.00x10^-3^ | .30 | 2.96x10^-23^ | -.028 | 3.09x10^-5^ | |
|  | L_S_front_inf | 5.06 | 0.98 | 15.93 | .01 | .30 | 2.96x10^-23^ | -.016 | .01 | |
|  | R_S_precentral_sup_part | 11.69 | 4.06 | 56.58 | .03 | .30 | 2.96x10^-23^ | -.028 | 2.32x10^-5^ | |
|  | R_S_occipital_ant | 7.06 | 2.10 | 26.65 | 4.00x10^-3^ | .30 | 2.96x10^-23^ | .019 | 4.22x10^-3^ | |
|  | L_G_precentral | 5.79 | 1.76 | 17.47 | <0.001 | .30 | 2.96x10^-23^ | -.019 | 3.43x10^-3^ | |
|  | L_G_temp_sup_Lateral | 8.98 | 2.78 | 44.50 | .02 | .30 | 2.96x10^-23^ | -.022 | 7.78x10^-4^ | |
|  | R_S_front_sup | 8.89 | 2.95 | 40.80 | .02 | .30 | 2.96x10^-23^ | -.023 | 5.75x10^-3^ | |
|  | R_S_precentral_inf_part | 7.65 | 0.19 | 51.70 | .05 | .30 | 2.96x10^-23^ | .016 | .01 | |
|  | L_S_front_sup | 7.60 | 2.46 | 30.61 | 9.00x10^-3^ | .30 | 2.96x10^-23^ | -.021 | 6.07x10^-4^ | |
|  | L_S_oc_sup_and_transversal | 9.97 | 2.60 | 49.31 | .03 | .30 | 2.96x10^-23^ | -.023 | .02 | |
|  |  |  |  |  |  |  |  |  |  | |
| Leukocyte | L_S_precentral_sup_part | 3.91 | 0.16 | 11.67 | .05 | .07 | 4.03x10^-23^ | -.057 | .04 | |
|  | R_G_pariet_inf_Angular | 6.81 | 0.78 | 31.64 | .03 | .07 | 4.03x10^-23^ | -.072 | 9.69x10^-3^ | |
|  | R_G_and_S_occipital_inf | 5.69 | 2.03 | 14.07 | <0.001 | .07 | 4.03x10^-23^ | .089 | 1.26x10^-3^ | |
|  |  |  |  |  |  |  |  |  |  | |
| Lymphocyte | R_S_front_inf | 5.51 | 1.54 | 17.16 | .01 | .05 | 3.47x10^-18^ | -.103 | 2.77x10^-3^ | |
|  | R_G_front_middle | 6.25 | 2.07 | 20.14 | .01 | .05 | 3.47x10^-18^ | -.113 | 1.06x10^-3^ | |
|  | L_S_precentral_sup_part | 3.81 | 0.49 | 11.03 | .02 | .05 | 3.47x10^-18^ | -.080 | .02 | |
|  | R_G_pariet_inf_Angular | 5.78 | 0.69 | 27.07 | .03 | .05 | 3.47x10^-18^ | -.086 | .01 | |
|  |  |  |  |  |  |  |  |  |  | |
| Monocyte | R_S_precentral_sup_part | 5.26 | 1.14 | 19.17 | .01 | .02 | 1.03x10^-16^ | -.176 | 6.29x10^-3^ | |
|  | L_caudate nucleus | 4.23 | 0.74 | 13.27 | .01 | .02 | 1.03x10^-16^ | -.159 | .01 | |
| Data are presented as proportion mediated (%) with 95% confidence interval (CI), β coefficients for exposure→inflammation and inflammation→SF coupling paths.  Abbreviations: CI, confidence interval; CRP, C‑reactive protein; SF, structure‑function coupling.  a. Effect of the first half of the mediation pathway; b. Effect of the second half of the mediation pathway. | | | | | | | | | |  |

| **eTable 22. Mediating effect of inflammatory markers on the relationship between adolescence smoking initiation and structure-function (SF) coupling in critical regions** | | | | | | | | | |  |  |
| --- | --- | --- | --- | --- | --- | --- | --- | --- | --- | --- | --- |
| **Inflammation mediator** | **Brain region** | **Proportion Mediated (%)** | | | | **β ^a^** | ***p* value ^a^** | **β ^b^** | ***p* value ^b^** | |  |
|  |  | **Value** | **95 CI (Lower)** | **95 CI**  **(Upper)** | ***p* value** |  |  |  |  |  |  |
| CRP | R_G_front_middle | 4.97 | 2.17 | 9.73 | <0.001 | .19 | 7.63x10^-33^ | -.022 | 3.68x10^-4^ | |  |
|  | R_G_pariet_inf_Angular | 11.22 | 4.79 | 42.46 | .01 | .19 | 7.63x10^-33^ | -.026 | 2.81x10^-5^ | |  |
|  | L_S_precentral_sup_part | 8.49 | 4.26 | 19.87 | <0.001 | .19 | 7.63x10^-33^ | -.027 | 7.13x10^-6^ | |  |
|  | R_S_front_inf | 7.85 | 3.89 | 17.34 | <0.001 | .19 | 7.63x10^-33^ | -.027 | 1.17x10^-5^ | |  |
|  | L_S_front_inf | 6.71 | 1.36 | 25.55 | .02 | .19 | 7.63x10^-33^ | -.016 | 9.75x10^-3^ | |  |
|  | R_S_precentral_sup_part | 8.86 | 4.34 | 22.02 | <0.001 | .19 | 7.63x10^-33^ | -.027 | 1.17x10^-5^ | |  |
|  | R_S_front_sup | 6.83 | 2.96 | 16.42 | <0.001 | .19 | 7.63x10^-33^ | -.022 | 2.42x10^-4^ | |  |
|  | R_S_precentral_inf_part | 3.06 | 0.58 | 6.53 | .01 | .19 | 7.63x10^-33^ | -.015 | .01 | |  |
|  | L_S_front_sup | 6.38 | 2.59 | 15.19 | <0.001 | .19 | 7.63x10^-33^ | -.021 | 5.59x10^-4^ | |  |
|  | R_S_subparietal | -4.62 | -12.97 | -0.56 | .02 | .19 | 7.63x10^-33^ | .013 | .03 | |  |
|  | L_G_front_middle | 3.99 | 0.70 | 9.46 | .01 | .19 | 7.63x10^-33^ | -.015 | .02 | |  |
|  | L_S_suborbital | 3.42 | 0.20 | 8.46 | .04 | .19 | 7.63x10^-33^ | -.013 | .04 | |  |
|  |  |  |  |  |  |  |  |  |  | |  |
| Leukocyte | L_S_precentral_sup_part | 6.18 | 1.63 | 17.28 | .01 | .05 | 1.59x10^-39^ | -.069 | 6.12x10^-3^ | |  |
|  | R_S_precentral_sup_part | 5.40 | 0.94 | 14.69 | .02 | .05 | 1.59x10^-39^ | -.062 | .01 | |  |
|  | R_G_pariet_inf_Angular | 6.40 | 0.10 | 26.23 | .05 | .05 | 1.59x10^-39^ | -.054 | .03 | |  |
|  |  |  |  |  |  |  |  |  |  | |  |
| Lymphocyte | R_S_front_inf | 5.42 | 1.18 | 13.16 | .01 | .04 | 6.16x10^-42^ | -.082 | 8.72x10^-3^ | |  |
|  | R_G_front_middle | 5.15 | 1.94 | 10.03 | <0.001 | .04 | 6.16x10^-42^ | -.104 | 8.96x10^-4^ | |  |
|  | R_S_precentral_inf_part | 3.55 | 0.62 | 7.44 | .02 | .04 | 6.16x10^-42^ | -.077 | .01 | |  |
|  | L_S_precentral_sup_part | 7.31 | 2.38 | 19.17 | <0.001 | .04 | 6.16x10^-42^ | -.097 | 2.03x10^-3^ | |  |
|  | R_S_precentral_sup_part | 5.26 | 0.66 | 14.53 | .02 | .04 | 6.16x10^-42^ | -.072 | .02 | |  |
|  | R_G_pariet_inf_Angular | 8.28 | 1.77 | 32.87 | .02 | .04 | 6.16x10^-42^ | -.085 | 6.55x10^-3^ | |  |
|  | R_G_and_S_cingul_Mid_Ant | -4.16 | -10.71 | -0.25 | .04 | .04 | 6.16x10^-42^ | .064 | .04 | |  |
|  |  |  |  |  |  |  |  |  |  | |  |
| Lymphocyte % | L_G_temp_sup_G_T_transv | -1.39 | -4.57 | -0.21 | .02 | .37 | 8.17x10^-4^ | .002 | 0.01 | |  |
|  |  |  |  |  |  |  |  |  |  | |  |
| Monocyte | R_S_precentral_sup_part | 4.26 | 1.61 | 11.43 | <0.001 | .01 | 3.75x10^-14^ | -.200 | 7.88x10^-4^ | |  |
|  | L_caudate nucleus | 2.51 | 0.51 | 5.87 | .01 | .01 | 3.75x10^-14^ | -.151 | .01 | |  |
| Data are presented as proportion mediated (%) with 95% confidence interval (CI), β coefficients for exposure→inflammation and inflammation→SF coupling paths.  Abbreviations: CI, confidence interval; CRP, C‑reactive protein; SF, structure‑function coupling.  a. Effect of the first half of the mediation pathway; b. Effect of the second half of the mediation pathway. | | | | | | | | | | |  |

| **eTable 23. Mediating effect of inflammatory markers on the relationship between adulthood smoking initiation and structure-function (SF) coupling in critical regions** | | | | | | | | | |  |  |
| --- | --- | --- | --- | --- | --- | --- | --- | --- | --- | --- | --- |
| **Inflammation mediator** | **Brain region** | **Proportion Mediated(%)** | | | | **β ^a^** | ***p* value ^a^** | **β ^b^** | ***p* value ^b^** | |  |
|  |  | **Value** | **95 CI (Lower)** | **95 CI**  **(Upper)** | ***p* value** |  |  |  |  |  |  |
| CRP | R_G_front_middle | 3.81 | 1.34 | 10.65 | <0.001 | .15 | 2.20x10^-11^ | -.020 | 1.75x10^-3^ | |  |
|  | R_G_pariet_inf_Angular | 5.28 | 2.33 | 15.55 | 1.00x10^-3^ | .15 | 2.20x10^-11^ | -.026 | 5.65x10^-5^ | |  |
|  | L_S_precentral_sup_part | 7.85 | 2.43 | 41.00 | .03 | .15 | 2.20x10^-11^ | -.027 | 3.62x10^-5^ | |  |
|  | R_S_precentral_sup_part | 7.68 | 2.57 | 36.77 | .03 | .15 | 2.20x10^-11^ | -.027 | 2.50x10^-5^ | |  |
|  | R_S_front_sup | 6.62 | 1.94 | 32.85 | .03 | .15 | 2.20x10^-11^ | -.023 | 2.95x10^-4^ | |  |
|  | L_S_front_sup | 4.82 | 1.65 | 16.92 | 7.00x10^-3^ | .15 | 2.20x10^-11^ | -.021 | 1.05x10^-3^ | |  |
|  | L_S_oc_sup_and_transversal | 3.66 | 0.99 | 10.83 | 4.00x10^-3^ | .15 | 2.20x10^-11^ | .018 | 6.71x10^-3^ | |  |
|  | R_S_subparietal | -4.07 | -14.74 | -0.83 | .02 | .15 | 2.20x10^-11^ | .017 | .01 | |  |
|  | L_G_front_middle | 2.37 | 0.34 | 6.14 | .02 | .15 | 2.20x10^-11^ | -.015 | 0.02 | |  |
|  | L_S_suborbital | 3.60 | 0.96 | 11.33 | 4.00x10^-3^ | .15 | 2.20x10^-11^ | -.018 | 6.55x10^-3^ | |  |
|  |  |  |  |  |  |  |  |  |  | |  |
| Leukocyte | R_G_pariet_inf_Angular | 4.43 | 1.19 | 13.71 | .01 | .04 | 7.46x10^-15^ | -.076 | 4.90x10^-3^ | |  |
|  | L_S_oc_middle_and_Lunatus | 4.12 | 0.66 | 15.00 | .02 | .04 | 7.46x10^-15^ | .064 | .02 | |  |
|  |  |  |  |  |  |  |  |  |  | |  |
| Lymphocyte | R_S_front_inf | 8.04 | 0.92 | 42.35 | .04 | .04 | 1.77x10^-19^ | -.102 | 2.53x10^-3^ | |  |
|  | R_G_front_middle | 5.63 | 2.05 | 15.13 | 1.00x10^-3^ | .04 | 1.77x10^-19^ | -.113 | 7.45x10^-4^ | |  |
|  | R_G_pariet_inf_Angular | 5.19 | 1.43 | 16.00 | 3.00x10^-3^ | .04 | 1.77x10^-19^ | -.097 | 3.94x10^-3^ | |  |
|  |  |  |  |  |  |  |  |  |  | |  |
| Monocyte | R_S_precentral_sup_part | 3.54 | 0.73 | 13.11 | .01 | .01 | 6.64x10^-9^ | -.169 | 7.56x10^-3^ | |  |
|  | L_S_oc_sup_and_transversal | 4.56 | 1.84 | 13.70 | <0.001 | .01 | 6.64x10^-9^ | .239 | 1.49x10^-4^ | |  |
|  | L_caudate nucleus | 2.07 | 0.28 | 5.44 | .02 | .01 | 6.64x10^-9^ | -.146 | .02 | |  |
| Data are presented as proportion mediated (%) with 95% confidence interval (CI), β coefficients for exposure→inflammation and inflammation→SF coupling paths.  Abbreviations: CI, confidence interval; CRP, C‑reactive protein; SF, structure‑function coupling.  a. Effect of the first half of the mediation pathway; b. Effect of the second half of the mediation pathway. | | | | | | | | | |  | |
|  | | | | | | | | | |  | |

| **eTable 24. Interactive effects between in utero exposure to tobacco and age of smoking initiation on inflammatory markers** | | | | | | | | | | | | |
| --- | --- | --- | --- | --- | --- | --- | --- | --- | --- | --- | --- | --- |
| **Inflammation marker** | **Mean inflammation levels (95% CI)** | | | | | | | | |  | ***F* value ^a^** | ***p* value*** |
|  | **Unexposed** | | | |  | **Exposed** | | | | |  |  |
|  | **Childhood** | **Adolescence** | **Adulthood** | **Never-smokers** |  | **Childhood** | **Adolescence** | **Adulthood** | **Never-smokers** | |  |  |
| CRP | 0.23 (0.21-0.25) | 0.16 (0.15-0.18) | 0.13 (0.12-0.15) | 0.02 (0.01-0.03) |  | 0.28 (0.26-0.30) | 0.21 (0.19-0.22) | 0.20 (0.17-0.22) | 0.04 (0.03-0.05) | | 5.15 | 1.47x10^-3^ |
| Leukocyte | 0.30 (0.28-0.32) | 0.18 (0.17-0.19) | 0.18 (0.16-0.19) | -0.06 (-0.07- -0.05) |  | 0.36 (0.33-0.38) | 0.24 (0.22-0.25) | 0.25 (0.22-0.27) | -0.06 (-0.07- -0.05) | | 21.74 | 4.54X10^-14^ |
| Lymphocyte | 0.21 (0.19-0.23) | 0.13 (0.12-0.15) | 0.16 (0.14-0.17) | -0.05 (-0.06- -0.04) |  | 0.25 (0.23-0.28) | 0.18 (0.16-0.19) | 0.18 (0.16-0.21) | -0.06 (-0.07- -0.05) | | 8.66 | 9.61x10^-6^ |
| Lymphocyte % | -0.024 (-0.046- -0.003) | -0.008 (-0.02-0.005) | 0.02 (0.01-0.04) | 0.004 (-0.005-0.01) |  | -0.04 (-0.06- -0.01) | -0.01 (-0.03-0.004) | -0.01 (-0.04-0.01) | -0.02 (-0.03- -0.007) | | 0.97 | .40 |
| Monocyte | 0.21 (0.19-0.23) | 0.14 (0.13-0.15) | 0.12 (0.10-0.14) | -0.01 (-0.02- -0.003) |  | 0.22 (0.20-0.25) | 0.17 (0.16-0.19) | 0.17 (0.15-0.19) | -0.007 (-0.01-0.004) | | 7.22 | 7.67x10^-5^ |
| Monocyte % | 0.002 (-0.02-0.02) | 0.02 (0.004-0.03) | -0.004 (-0.02-0.01) | 0.03 (0.02-0.04) |  | -0.04 (-0.06- -0.01) | 0.004 (-0.01-0.02) | -0.01 (-0.03-0.02) | 0.04 (0.03-0.05) | | 1.61 | .18 |
| Neutrophill | 0.26 (0.24-0.28) | 0.14 (0.13-0.15) | 0.13 (0.12-0.15) | -0.06 (-0.07- -0.05) |  | 0.32 (0.30-0.34) | 0.20 (0.18-0.21) | 0.20 (0.17-0.22) | -0.05 (-0.06- -0.04) | | 19.52 | 1.19x10^-12^ |
| Neutrophill % | 0.01 (-0.01-0.03) | -0.01 (-0.02-0.002) | -0.03 (-0.05- -0.02) | -0.02 (-0.03- -0.01) |  | 0.04 (0.01-0.06) | -0.001 (-0.02-0.01) | 0.0004 (-0.02-0.02) | 0.001 (-0.01-0.01) | | 1.29 | .28 |
| Platelet | 0.01 (-0.006-0.04) | -0.04 (-0.05- -0.03) | -0.04 (-0.06- -0.02) | -0.08 (-0.09- -0.07) |  | 0.01 (-0.01-0.03) | -0.02 (-0.04- -0.01) | -0.05 (-0.08- -0.03) | -0.09 (-0.10- -0.07) | | 2.80 | .04 |
| Data are presented as mean (95% confidence interval) of log‑transformed and z‑standardized inflammatory marker levels. Means are adjusted for covariates.  Abbreviations: CI, confidence interval; CRP, C‑reactive protein.  * All *p* values are two-sided. a. This is the F value for the 2x4 ANCOVA. | | | | | | | | | | | | |

| **eTable 25.** **Associations between age of smoking initiation and incident depression stratified by BMI, ethnicity, alcohol status, deprivation, and age** | | | | | | | | | |
| --- | --- | --- | --- | --- | --- | --- | --- | --- | --- |
| **Characteristic** | | **Childhood** | |  | **Adolescence** | |  | **Adulthood** | |
|  |  | **HR (95% CI)** | *p* _interaction_***** |  | **HR (95% CI)** | *p* _interaction_ |  | **HR (95% CI)** | *p* _interaction_ |
| BMI | Obesity | 1.59 (1.44-1.76) | 6.39x10^-3^ |  | 1.23 (1.14-1.32) | .06 |  | 1.21 (1.09-1.34) | 6.60x10^-3^ |
|  | Overweight | 2.06 (1.87-2.26) | 2.64x10^-9^ |  | 1.54 (1.45-1.64) | 4.10x10^-10^ |  | 1.41 (1.29-1.54) | 8.47x10^-7^ |
|  | Normal | 2.46 (2.18-2.78) | Reference |  | 1.66 (1.53-1.79) | Reference |  | 1.68 (1.51-1.86) | Reference |
| Ethnicity | White | 1.91 (1.80-2.03) | 9.52x10^-3^ |  | 1.43 (1.38-1.49) | 6.15x10^-4^ |  | 1.38 (1.30-1.46) | 3.24x10^-6^ |
|  | Ethnic minority | 2.93 (2.12-4.03) | Reference |  | 2.31 (1.85-2.88) | Reference |  | 1.97 (1.56-2.49) | Reference |
| Alcohol status | Alcohol use | 1.90 (1.78-2.02) | 2.25x10^-3^ |  | 1.43 (1.37-1.49) | 1.59x10^-3^ |  | 1.37 (1.29-1.46) | .02 |
|  | Never | 2.27 (1.91-2.69) | Reference |  | 1.66 (1.46-1.88) | Reference |  | 1.68 (1.42-1.99) | Reference |
| Deprivation | Higher | 2.12 (1.95-2.30) | 2.97x10^-4^ |  | 1.53 (1.44-1.63) | .07 |  | 1.44 (1.33-1.57) | .42 |
|  | Middle | 1.78 (1.59-2.00) | .35 |  | 1.37 (1.27-1.47) | .45 |  | 1.43 (1.29-1.58) | .49 |
|  | Lower | 1.71 (1.49-1.97) | Reference |  | 1.47 (1.36-1.59) | Reference |  | 1.36 (1.21-1.52) | Reference |
| Age | Older age | 1.70 (1.45.1.99) | .002 |  | 1.46 (1.34-1.59) | .26 |  | 1.27 (1.14-1.42) | .01 |
|  | Middle age | 2.00 (1.87-2.13) | Reference |  | 1.46 (1.39-1.53) | Reference |  | 1.46 (1.37-1.56) | Reference |
| Data are presented as hazard ratio (HR) with 95% confidence interval (CI) for each stratum. p~interaction~ values are from models including an interaction term between smoking initiation age and the stratifying covariate, adjusted for other covariates.  Abbreviations: BMI, body mass index; CI, confidence interval; HR, hazard ratio.  * All p values are two-sided. p interaction were FDR-corrected, with significance defined as p < .05. | | | | | | | | | |

| **eTable 26. Associations between age of smoking initiation and incident anxiety stratified by deprivation, alcohol status and ethnicity** | | | | | | | | | |
| --- | --- | --- | --- | --- | --- | --- | --- | --- | --- |
| **Characteristic** | | **Childhood** | |  | **Adolescence** | |  | **Adulthood** | |
|  |  | **HR (95% CI)** | *p* _interaction_ ***** |  | **HR (95% CI)** | *p* _interaction_ |  | **HR (95% CI)** | *p* _interaction_ |
| Deprivation | Higher | 1.75 (1.59-1.91) | 3.97x10^-3^ |  | 1.44 (1.35-1.54) | 3.72x10^-3^ |  | 1.30 (1.19-1.43) | .37 |
|  | Middle | 1.50 (1.32-1.71) | .56 |  | 1.26 (1.17-1.35) | .89 |  | 1.33 (1.21-1.48) | .25 |
|  | Lower | 1.41 (1.22-1.64) | Reference |  | 1.26 (1.16-1.36) | Reference |  | 1.21 (1.09-1.36) | Reference |
| Alcohol status | Alcohol use | 1.57 (1.47-1.69) | .03 |  | 1.30 (1.25-1.36) | 1.81x10^-3^ |  | 1.26 (1.19-1.34) | .09 |
|  | Never | 1.78 (1.46-2.16) | Reference |  | 1.52 (1.33-1.73) | Reference |  | 1.44 (1.20-1.74) | Reference |
| Ethnicity | White | 1.58 (1.48-1.67) | .51 |  | 1.31 (1.26-1.37) | .23 |  | 1.26 (1.19-1.33) | 3.74x10^-3^ |
|  | Ethnic minority | 2.27 (1.58-3.25) | Reference |  | 1.67 (1.31-2.14) | Reference |  | 1.75 (1.37-2.23) | Reference |
| Data are presented as hazard ratio (HR) with 95% confidence interval (CI) for each stratum. p~interaction~ values are from models including an interaction term between smoking initiation age and the stratifying covariate, adjusted for other covariates.  Abbreviations: BMI, body mass index; CI, confidence interval; HR, hazard ratio.  * All *p* values are two-sided. *p* _interaction_ were FDR-corrected, with significance defined as *p* < .05. | | | | | | | | |  |

| **eTable 27. Associations between in utero exposure to tobacco smoke and inflammation markers by covariates** | | | | | | | | | | |
| --- | --- | --- | --- | --- | --- | --- | --- | --- | --- | --- |
| **Characteristic** | | **CRP** | **Leukocyte** | **Lymphocyte** | **Lymphocyte %** | **Monocyte** | **Monocyte %** | **Neutrophill** | **Neutrophill %** | **Platelet** |
| Ethnicity |  |  | *p* _interaction_<0.001 | *p* _interaction_<0.001 | *p* _interaction_<0.001 | *p* _interaction_=1.00x10^-5^ |  | *p* _interaction_<0.001 | *p* _interaction_<0.001 | *p* _interaction_=8.68x10^-3^ |
|  | Ethnic Minority |  | 0.05(0.002-0.098) | 0.15(0.10-0.20) | 0.15(0.10-0.20) | -0.04(-0.09-0.004) |  | -0.01(-0.06-0.03) | -0.12(-0.17- -0.08) | -0.07(-0.12- -0.02) |
|  | White |  | 0.04(0.03-0.05) | -0.01(-0.01- -0.0006) | -0.05(-0.06- -0.04) | 0.08(0.07-0.09) |  | 0.04(0.03-0.04) | 0.03(0.02-0.03) | -0.06(-0.07- -0.05) |
|  |  |  |  |  |  |  |  |  |  |  |
| Deprivation |  | *p* _interaction_<0.001 | *p* _interaction_<0.001 | *p* _interaction_=.02 | *p* _interaction_=2.40x10^-4^ | *p* _interaction_=.03 |  | *p* _interaction_<0.001 | *p* _interaction_=1.10x10^-4^ | *p* _interaction_=3.10x10^-4^ |
|  | Lower | 0.18(0.17-0.19) | 0.08(0.07-0.10) | 0.20(0.19-0.21) | 0.19(0.17-0.20) | 0.01(-0.004-0.02) |  | 0.02(0.004-0.030) | -0.17(-0.18- -0.15) | -0.07(-0.08- -0.06) |
|  | Higher | -0.01(-0.02- -0.003) | -0.08(-0.09- -0.07) | 0.09(0.08-0.10) | 0.21(0.20-0.22) | -0.06(-0.07- -0.05) |  | -0.14(-0.15- -0.13) | -0.20(-0.21- -0.19) | -0.11(-0.12- -0.10) |
|  | Middle | 0.07(0.06-0.08) | -0.04(-0.06- -0.03) | 0.15(0.13-0.16) | 0.24(0.23-0.26) | -0.04(-0.05- -0.02) |  | -0.13(-0.14- -0.11) | -0.23(-0.24- -0.22) | -0.11(-0.12- -0.10) |
|  |  |  |  |  |  |  |  |  |  |  |
| Income |  |  | *p* _interaction_=0.045 |  |  |  |  | *p* _interaction_=0.048 |  |  |
|  | Unknown |  | -0.01(-0.04-0.01) |  |  |  |  | -0.09(-0.11- -0.07) |  |  |
|  | High |  | -0.17(-0.20- -0.14) |  |  |  |  | -0.25(-0.28- -0.23) |  |  |
|  | Low |  | 0.03(0.02-0.04) |  |  |  |  | -0.04(-0.05- -0.03) |  |  |
|  | Middle |  | -0.08(-0.10- -0.07) |  |  |  |  | -0.17(-0.18- -0.15) |  |  |
|  |  |  |  |  |  |  |  |  |  |  |
| BMI |  | *p* _interaction_=.03 |  |  |  |  |  |  | *p* _interaction_=.03 |  |
|  | Normal | -0.39(-0.40- -0.38) |  |  |  |  |  |  | -0.16(-0.18- -0.15) |  |
|  | Overweight | 0.08(0.07-0.10) |  |  |  |  |  |  | -0.23(-0.24- -0.22) |  |
|  | Obesity | 0.62(0.61-0.63) |  |  |  |  |  |  | -0.21(-0.22- -0.20 |  |
| Alcohol status |  |  |  |  |  |  | *p* _interaction_=6.53x10^-3^ | *p* _interaction_=.02 |  |  |
|  | Never |  |  |  |  |  | -0.11(-0.13- -0.08) | 0.06(0.04-0.09) |  |  |
|  | Alcohol use |  |  |  |  |  | -0.025(-0.03- -0.02) | -0.10(-0.11- -0.09) |  |  |
|  |  |  |  |  |  |  |  |  |  |  |
| Comparative body size at age 10 |  |  |  |  |  |  | *p* _interaction_=.01 |  |  |  |
|  | About average |  |  |  |  |  | -0.11(-0.12- -0.10) |  |  |  |
|  | Plumper |  |  |  |  |  | -0.02(-0.03- -0.002) |  |  |  |
|  | Thinner |  |  |  |  |  | -0.05(-0.07- -0.04) |  |  |  |
| Data are presented as mean (95% confidence interval) of inflammatory marker levels (log‑transformed and z‑standardized) for each stratum, or as β (95% CI) for the difference between exposed and unexposed within each stratum.  Abbreviations: BMI, body mass index; CI, confidence interval; CRP, C‑reactive protein.  * All *p* values were two-sided. *p* _interaction_ were FDR-corrected, with significance defined as *p* < .05. | | | | | | | | | | |

| **eTable 28. Associations between age of smoking initiation and Inflammation makers by covariates** | | | | | | | | | | | |
| --- | --- | --- | --- | --- | --- | --- | --- | --- | --- | --- | --- |
| **Characteristics** | | **Smoking initiation age** | **CRP** | **Leukocyte** | **Lymphocyte** | **Lymphocyte %** | **Monocyte** | **Monocyte %** | **Neutrophill** | **Neutrophill %** | **Platelet** |
| Age |  |  |  | *p* _interaction_<0.001 | *p* _interaction_<0.001 | *p* _interaction_<0.001 |  |  | *p* _interaction_=7.81x10^-3^ | *p* _interaction_<0.001 |  |
|  | Middle age | adulthood |  | 0.12(0.11-0.14) | 0.32(0.30-0.33) | 0.29(0.27-0.30) |  |  | -0.22(-0.23- -0.21) | -0.25(-0.26- -0.23) |  |
|  |  | adolescence |  | 0.11(0.10-0.12) | 0.30(0.29-0.32) | 0.28(0.27-0.29) |  |  | 0.02(0.01-0.03) | -0.24(-0.26- -0.23) |  |
|  |  | childhood |  | 0.27(0.25-0.28) | 0.42(0.40-0.43) | 0.26(0.25-0.28) |  |  | 0.01(0.002-0.024) | -0.22(-0.24- -0.20) |  |
|  | Older age | adulthood |  | 0.15(0.13-0.17) | 0.22(0.20-0.24) | 0.15(0.12-0.17) |  |  | 0.16(0.15-0.18) | -0.15(-0.18- -0.13) |  |
|  |  | adolescence |  | 0.17(0.16-0.19) | 0.21(0.19-0.23) | 0.12(0.11-0.14) |  |  | -0.12(-0.14- -0.11) | -0.14(-0.16- -0.12) |  |
|  |  | childhood |  | 0.29(0.25-0.32) | 0.24(0.21-0.28) | 0.06(0.02-0.09) |  |  | 0.06(0.04-0.09) | -0.08(-0.12- -0.04) |  |
|  |  |  |  |  |  |  |  |  |  |  |  |
| Gender |  |  | *p* _interaction_<0.001 | *p* _interaction_<0.001 | *p* _interaction_=1.60x10^-4^ | *p* _interaction_<0.001 | *p* _interaction_<0.001 | *p* _interaction_=.02 | *p* _interaction_<0.001 | *p* _interaction_<0.001 | *p* _interaction_=.046 |
|  | Female | adulthood | 0.14(0.12-0.16) | 0.17(0.15-0.19) | 0.17(0.15-0.18) | 0.06(0.05-0.08) | 0.30(0.29-0.32) | 0.19(0.18-0.21) | 0.09(0.08-0.11) | -0.15(-0.17- -0.13) | -0.30(-0.32- -0.28) |
|  |  | adolescence | 0.16(0.15-0.17) | 0.17(0.15-0.18) | 0.15(0.14-0.17) | 0.06(0.05-0.07) | 0.31(0.30-0.32) | 0.21(0.20-0.22) | 0.09(0.08-0.10) | -0.15(-0.16- -0.13) | -0.29(-0.31- -0.28) |
|  |  | childhood | 0.28(0.26-0.30) | 0.29(0.27-0.31) | 0.26(0.24-0.28) | 0.07(0.05-0.08) | 0.38(0.36-0.40) | 0.18(0.16-0.20) | 0.20(0.18-0.22) | -0.15(-0.17- -0.13) | -0.24(-0.26- -0.22 |
|  | Male | adulthood | 0.14(0.12-0.16) | 0.13(0.11-0.14) | 0.39(0.38-0.41) | 0.38(0.36-0.39) | -0.19(-0.20- -0.17) | -0.30(-0.32- -0.29) | 0.01(-0.01-0.03) | -0.26(-0.27- -0.24) | 0.15(0.13-0.17) |
|  |  | adolescence | 0.18(0.17-0.19) | 0.12(0.11-0.13) | 0.39(0.38-0.41) | 0.38(0.37-0.39) | -0.18(-0.19- -0.17) | -0.30(-0.31- -0.28) | 0.004(-0.01-0.02) | -0.26(-0.28- -0.25) | 0.16(0.15-0.17) |
|  |  | childhood | 0.28(0.25-0.30) | 0.31(0.28-0.33) | 0.49(0.47-0.52) | 0.32(0.29-0.34) | -0.11(-0.13- -0.08) | -0.35(-0.37- -0.32) | 0.20(0.17-0.22) | -0.19(-0.22- -0.16) | 0.22(0.19-0.24) |
|  |  |  |  |  |  |  |  |  |  |  |  |
|  |  |  |  | *p* _interaction_<0.001 |  | *p* _interaction_<0.001 |  |  | *p* _interaction_<0.001 | *p* _interaction_<0.001 |  |
| Ethnicity | Ethnic Minority | adulthood |  | 0.14(0.09-0.19) |  | 0.47(0.42-0.52) |  |  | -0.04(-0.09-0.01) | -0.41(-0.46- -0.37) |  |
|  |  | adolescence |  | 0.16(0.12-0.21) |  | 0.39(0.34-0.43) |  |  | 0.16(0.14-0.17) | -0.33(-0.38- -0.28) |  |
|  |  | childhood |  | 0.31(0.23-0.40) |  | 0.31(0.23-0.39) |  |  | 0.01(-0.03-0.06) | -0.25(-0.33- -0.17) |  |
|  | White | adulthood |  | 0.18(0.17-0.19) |  | -0.05(-0.06- -0.04) |  |  | 0.16(0.15-0.16) | 0.03(0.02-0.04) |  |
|  |  | adolescence |  | 0.18(0.17-0.19) |  | -0.06(-0.07- -0.05) |  |  | 0.19(0.10-0.27) | 0.03(0.03-0.04) |  |
|  |  | childhood |  | 0.33(0.32-0.34) |  | -0.08(-0.09- -0.06) |  |  | 0.30(0.29-0.32) | 0.06(0.05-0.08) |  |
|  |  |  |  |  |  |  |  |  |  |  |  |
| Deprivation |  |  | *p* _interaction_<0.001 | *p* _interaction_<0.001 | *p* _interaction_<0.001 |  | *p* _interaction_<0.001 | *p* _interaction_<0.001 | *p* _interaction_<0.001 | *p* _interaction_=0.04 | *p* _interaction_=1.00x10^-5^ |
|  | Lower | adulthood | 0.09(0.06-0.11) | 0.06(0.04-0.08) | 0.23(0.21-0.25) |  | 0.03(0.01-0.05) | -0.02(-0.040- -0.003) | -0.05(-0.07- -0.03) | -0.24(-0.26- -0.22) | -0.06(-0.08- -0.03) |
|  |  | adolescence | 0.10(0.08-0.11) | 0.06(0.05-0.08) | 0.22(0.21-0.24) |  | 0.04(0.03-0.06) | -0.01(-0.02-0.01) | -0.04(-0.05- -0.02) | -0.23(-0.25- -0.22) | -0.08(-0.09- -0.06) |
|  |  | childhood | 0.16(0.13-0.19) | 0.14(0.11-0.17) | 0.29(0.26-0.32) |  | 0.10(0.07-0.13) | -0.01(-0.03-0.02) | 0.02(-0.01-0.05) | -0.24(-0.27- -0.21) | -0.05(-0.08- -0.03) |
|  | Higher | adulthood | 0.18(0.16-0.20) | 0.22(0.20-0.24) | 0.32(0.30-0.34) |  | 0.08(0.06-0.10) | -0.08(-0.10- -0.06) | 0.13(0.11-0.15) | -0.18(-0.20- -0.16) | -0.08(-0.10- -0.06) |
|  |  | adolescence | 0.23(0.22-0.25) | 0.24(0.23-0.26) | 0.33(0.31-0.34) |  | 0.10(0.08-0.11) | -0.08(-0.10- -0.07) | 0.15(0.14-0.17) | -0.17(-0.18- -0.15) | -0.05(-0.06- -0.03) |
|  |  | childhood | 0.38(0.35-0.40) | 0.43(0.41-0.46) | 0.45(0.43-0.47) |  | 0.19(0.17-0.22) | -0.13(-0.15- -0.11) | 0.34(0.32-0.36) | -0.12(-0.15- -0.10) | 0.03(0.005-0.048) |
|  | Middle | adulthood | 0.10(0.08-0.13) | 0.13(0.11-0.15) | 0.27(0.25-0.29) |  | 0.05(0.03-0.07) | -0.05(-0.07- -0.03) | 0.02(0.002-0.044) | -0.21(-0.24- -0.19) | -0.09(-0.11- -0.07) |
|  |  | adolescence | 0.15(0.13-0.16) | 0.11(0.09-0.12) | 0.26(0.24-0.27) |  | 0.06(0.05-0.08) | -0.02(-0.03- -0.01) | 0.004(-0.01-0.02) | -0.22(-0.23- -0.21) | -0.07(-0.09- -0.06) |
|  |  | childhood | 0.24(0.22-0.27) | 0.20(0.18-0.23) | 0.32(0.29-0.35) |  | 0.11(0.09-0.14) | -0.04(-0.06- -0.01) | 0.10(0.08-0.13) | -0.20(-0.23- -0.18) | -0.03(-0.04- -0.002) |
|  |  |  |  |  |  |  |  |  |  |  |  |
| Education |  |  | *p* _interaction_<0.001 | *p* _interaction_<0.001 | *p* _interaction_<0.001 | *p* _interaction_=3.00x10^-5^ | *p* _interaction_<0.001 | *p* _interaction_=.01 | *p* _interaction_<0.001 | *p* _interaction_=5.80x10^-4^ |  |
|  | Above college | adulthood | 0.01(-0.01-0.03) | 0.04(0.02-0.06) | 0.24(0.23-0.26) | 0.22(0.20-0.23) | -0.08(-0.10- -0.07) | 0.01(-0.01-0.03) | -0.23(-0.25- -0.21) | -0.05(-0.07- -0.03) |  |
|  |  | adolescence | 0.04(0.02-0.06) | 0.02(0.01-0.04) | 0.26(0.25-0.28) | 0.21(0.20-0.23) | -0.02(-0.04- -0.01) | 0.003(-0.01-0.02) | -0.25(-0.27- -0.23) | -0.07(-0.09- -0.06) |  |
|  |  | childhood | 0.21(0.17-0.24) | 0.18(0.14-0.21) | 0.25(0.22-0.29) | 0.33(0.29-0.36) | -0.06(-0.07- -0.05) | 0.09(0.06-0.12) | -0.24(-0.28- -0.21) | 0.06(0.02-0.09) |  |
|  | Less  than college | adulthood | 0.26(0.25-0.18) | 0.24(0.23-0.26) | 0.20(0.18-0.21) | 0.33(0.32-0.35) | -0.02(-0.03- -0.001) | 0.10(0.09-0.12) | -0.17(-0.19- -0.16) | 0.14(0.12-0.16) |  |
|  |  | adolescence | 0.29(0.28-0.31) | 0.24(0.22-0.25) | 0.18(0.17-0.19) | 0.31(0.30-0.32) | -0.10(-0.11- -0.08) | 0.12(0.11-0.13) | -0.16(-0.17- -0.15) | 0.14(0.13-0.15) |  |
|  |  | childhood | 0.40(0.39-0.42) | 0.38(0.36-0.40) | 0.16(0.14-0.17) | 0.41(0.39-0.43) | -0.03(-0.07- 0.001) | 0.19(0.17-0.21) | -0.13(-0.15- -0.11) | 0.28(0.26-0.30) |  |
|  |  |  |  |  |  |  |  |  |  |  |  |
| Income |  |  | *p* _interaction_<0.001 | *p* _interaction_<0.001 | *p* _interaction_<0.001 |  | *p* _interaction_<0.001 | *p* _interaction_=8.00x10^-5^ | *p* _interaction_<0.001 |  |  |
|  | Unknow | adulthood | 0.18(0.14-0.22) | 0.15(0.11-0.19) | 0.28(0.25-0.32) |  | -0.09(-0.10- -0.08) | -0.04(-0.08- -0.003) | 0.04(-0.0001-0.08) |  |  |
|  |  | adolescence | 0.17(0.14-0.19) | 0.14(0.11-0.16) | 0.27(0.24-0.29) |  | 0.07(0.03-0.10) | -0.02(-0.04-0.01) | 0.03(0.01-0.06) |  |  |
|  |  | childhood | 0.30(0.26-0.35) | 0.26(0.21-0.31) | 0.33(0.28-0.37) |  | 0.09(0.07-0.11) | -0.09(-0.13- -0.04) | 0.18(0.13-0.22) |  |  |
|  | High | adulthood | -0.05(-0.10- -0.001) | -0.04(-0.09- -0.03) | 0.22(0.16-0.27) |  | 0.11(0.07-0.16) | -0.03(-0.08-0.02) | -0.14(-0.19- -0.09) |  |  |
|  |  | adolescence | -0.03(-0.07- -0.01) | -0.07(-0.11- -0.03) | 0.22(0.18-0.25) |  | -0.17(-0.19- -0.15) | -0.03(-0.06-0.01) | -0.18(-0.21- -0.14) |  |  |
|  |  | childhood | 0.05(-0.04-0.14) | -0.02(-0.11-0.07) | 0.24(0.15-0.33) |  | -0.05(-0.10-0.01) | 0.05(-0.04-0.14) | -0.14(-0.23- -0.05) |  |  |
|  | Low | adulthood | 0.16(0.15-0.18) | 0.18(0.16-0.19) | 0.29(0.28-0.31) |  | -0.07(-0.10- -0.03) | -0.07(-0.08- -0.05) | 0.09(0.07-0.10) |  |  |
|  |  | adolescence | 0.21(0.20-0.22) | 0.19(0.17-0.20) | 0.28(0.27-0.29) |  | 0.01(-0.08-0.10) | -0.05(-0.06- -0.04) | 0.09(0.08-0.11) |  |  |
|  |  | childhood | 0.32(0.30-0.34) | 0.34(0.32-0.36) | 0.39(0.37-0.41) |  | -0.09(-0.10- -0.08) | -0.08(-0.10- -0.07) | 0.25(0.23-0.26) |  |  |
|  | Middle | adulthood | 0.03(0.01-0.06) | 0.06(0.03-0.09) | 0.25(0.23-0.28) |  | 0.09(0.08-0.10) | -0.02(-0.05-0.01) | -0.05(-0.08- -0.03) |  |  |
|  |  | adolescence | 0.04(0.02-0.06) | 0.02(0.002-0.042) | 0.24(0.22-0.26) |  | 0.17(0.16-0.19) | -0.02(-0.04- -0.003) | -0.09(-0.11- -0.07) |  |  |
|  |  | childhood | 0.17(0.13-0.20) | 0.13(0.09-0.17) | 0.33(0.29-0.36) |  | -0.12(-0.13- -0.11) | -0.05(-0.08- -0.01) | 0.01(-0.03-0.04) |  |  |
|  |  |  |  |  |  |  |  |  |  |  |  |
| BMI |  |  | *p* _interaction_<0.001 | *p* _interaction_<0.001 | *p* _interaction_<0.001 | *p* _interaction_=3.59x10^-3^ | *p* _interaction_=6.00x10^-5^ | *p* _interaction_<0.001 | *p* _interaction_<0.001 |  | *p* _interaction_<0.001 |
|  | Normal | adulthood | -0.32(-0.34- -0.30) | 0.004(-0.02-0.02) | 0.13(0.10-0.15) | 0.18(0.16-0.20) | -0.06(-0.08- -0.04) | -0.07(-0.09- -0.05) | -0.03(-0.05- -0.01) |  | -0.08(-0.10- -0.06) |
|  |  | adolescence | -0.26(-0.27- -0.24) | 0.03(0.01-0.04) | 0.13(0.12-0.15) | 0.17(0.15-0.18) | -0.05(-0.07- -0.03) | -0.07(-0.09- -0.06) | -0.005(-0.02-0.01) |  | -0.06(-0.07- -0.04) |
|  |  | childhood | -0.13(-0.15- -0.10) | 0.24(0.21-0.27) | 0.24(0.21-0.27) | 0.10(0.07-0.13) | 0.07(0.05-0.01) | -0.09(-0.12- -0.07) | 0.22(0.19-0.25) |  | 0.02(-0.01-0.05) |
|  | Overweight | adulthood | 0.14(0.13-0.16) | -0.13(-0.14- -0.13) | 0.28(0.26-0.30) | 0.25(0.23-0.27) | 0.05(0.03-0.06) | -0.04(-0.06- -0.03) | 0.01(-0.01-0.02) |  | -0.08(-0.09- -0.06) |
|  |  | adolescence | 0.18(0.17-0.19) | 0.12(0.10-0.14) | 0.26(0.25-0.27) | 0.23(0.21-0.24) | 0.05(0.03-0.06) | -0.04(-0.06- -0.03) | 0.02(0.005-0.031) |  | -0.06(-0.07- -0.04) |
|  |  | childhood | 0.25(0.23-0.27) | 0.12(0.11-0.13) | 0.35(0.33-0.37) | 0.22(0.20-0.25) | 0.10(0.08-0.12) | -0.08(-0.10- -0.05) | 0.13(0.11-0.15) |  | -0.01(-0.03-0.01) |
|  | Obesity | adulthood | 0.64(0.62-0.67) | 0.23(0.21-0.26) | 0.45(0.42-0.47) | 0.21(0.19-0.24) | 0.21(0.19-0.23) | -0.06(-0.08- -0.04) | 0.22(0.19-0.24) |  | -0.07(-0.10- -0.05) |
|  |  | adolescence | 0.63(0.62-0.64) | 0.36(0.34-0.38) | 0.44(0.43-0.46) | 0.23(0.22-0.25) | 0.24(0.22-0.26) | -0.01(-0.03-0.01) | 0.17(0.16-0.19) |  | -0.09(-0.10- -0.07) |
|  |  | childhood | 0.70(0.68-0.73) | 0.33(0.31-0.35) | 0.52(0.49-0.54) | 0.21(0.18-0.23) | 0.28(0.26-0.31) | -0.06(-0.09- -0.04) | 0.30(0.27-0.32) |  | -0.03(-0.05--0.004) |
|  |  |  |  |  |  |  |  |  |  |  |  |
| Alcohol status |  |  |  | *p* _interaction_<0.001 | *p* _interaction_<0.001 |  |  | *p* _interaction_<0.001 | *p* _interaction_<0.001 |  |  |
|  | Never | adulthood |  | 0.30(0.26-0.35) | 0.34(0.30-0.39) |  |  | -0.18(-0.22- -0.13) | 0.24(0.19-0.28) |  |  |
|  |  | adolescence |  | 0.28(0.25-0.31) | 0.34(0.31-0.37) |  |  | -0.13(-0.16- -0.09) | 0.20(0.17-0.23) |  |  |
|  |  | childhood |  | 0.47(0.42-0.53) | 0.50(0.45-0.55) |  |  | -0.20(-0.25- -0.15) | 0.38(0.32-0.43) |  |  |
|  | Alcohol use | adulthood |  | 0.13(0.12-0.14) | 0.27(0.26-0.29) |  |  | -0.04(-0.06- -0.03) | 0.03(0.01-0.04) |  |  |
|  |  | adolescence |  | 0.13(0.12-0.14) | 0.27(0.25-0.28) |  |  | -0.03(-0.05- -0.02) | 0.03(0.02-0.04) |  |  |
|  |  | childhood |  | 0.27(0.26-0.29) | 0.36(0.35-0.38) |  |  | -0.06(-0.08- -0.05) | 0.17(0.16-0.19) |  |  |
|  |  |  |  |  |  |  |  |  |  |  |  |
| Comparative body size at age 10 |  |  |  |  | *p* _interaction_=.04 |  |  |  |  |  | *p* _interaction_=.03 |
|  | About average | adulthood |  |  | 0.27(0.25-0.28) |  |  |  |  |  | -0.08(-0.10- -0.07) |
|  |  | adolescence |  |  | 0.26(0.25-0.27) |  |  |  |  |  | -0.07(-0.08- -0.05) |
|  |  | childhood |  |  | 0.35(0.33-0.37) |  |  |  |  |  | -0.03(-0.05- -0.01) |
|  | Plumper | adulthood |  |  | 0.31(0.28-0.34) |  |  |  |  |  | -0.08(-0.11- -0.05) |
|  |  | adolescence |  |  | 0.31(0.29-0.33) |  |  |  |  |  | -0.09(-0.11- -0.07) |
|  |  | childhood |  |  | 0.46(0.42-0.49) |  |  |  |  |  | -0.04(-0.07- -0.004) |
|  | Thinner | adulthood |  |  | 0.29(0.27-0.31) |  |  |  |  |  | -0.06(-0.08- -0.04) |
|  |  | adolescence |  |  | 0.27(0.26-0.28) |  |  |  |  |  | -0.05(-0.07- -0.04) |
|  |  | childhood |  |  | 0.36(0.34-0.39) |  |  |  |  |  | 0.03(0.003-0.05) |
|  |  |  |  |  |  |  |  |  |  |  |  |
| Comparative height at age 10 |  |  |  |  |  |  | *p* _interaction_=.04 | *p* _interaction_=3.97x10^-3^ |  |  |  |
|  | About average | adulthood |  |  |  |  | 0.04(0.03-0.06) | -0.07(-0.09- -0.05) |  |  |  |
|  |  | adolescence |  |  |  |  | 0.07(0.06-0.09) | -0.04(-0.05- -0.03) |  |  |  |
|  |  | childhood |  |  |  |  | 0.15(0.13-0.17) | -0.08(-0.10- -0.06) |  |  |  |
|  | Shorter | adulthood |  |  |  |  | 0.11(0.08-0.13) | -0.05(-0.08- -0.03) |  |  |  |
|  |  | adolescence |  |  |  |  | 0.10(0.08-0.12) | -0.07(-0.08- -0.05) |  |  |  |
|  |  | childhood |  |  |  |  | 0.22(0.19-0.25) | -0.05(-0.08- -0.02) |  |  |  |
|  | Taller | adulthood |  |  |  |  | 0.05(0.03-0.07) | -0.03(-0.05- -0.01) |  |  |  |
|  |  | adolescence |  |  |  |  | 0.04(0.03-0.06) | -0.02(-0.04- -0.01) |  |  |  |
|  |  | childhood |  |  |  |  | 0.09(0.06-0.12) | -0.10(-0.13- -0.07) |  |  |  |
| Data are presented as mean (95% confidence interval) of inflammatory marker levels (log‑transformed and z‑standardized) for each stratum, or as β (95% CI) for the difference between exposed and unexposed within each stratum.  Abbreviations: BMI, body mass index; CI, confidence interval; CRP, C‑reactive protein.  * All p values were two-sided. *p* _interaction_ were FDR-corrected, with significance defined as *p* < .05. | | | | | | | | | | | |

| **eTable 29.** **Associations between early-life tobacco exposure and structure-function (SF) coupling by BMI** | | | | | | | | |  |
| --- | --- | --- | --- | --- | --- | --- | --- | --- | --- |
|  |  | **In utero exposure to tobacco smoke** | |  | **Age of smoking initiation** | | | |  |
|  |  | **Exposed** | |  | **Adulthood** | | **Adolescence** | **Childhood** |  |
| **Characteristic** | | **R_G_insular_short** |  |  | | **L_G_and_S_cingul** | | | |
| BMI |  | *p* _interaction_=.03 | |  | *p* _interaction_=.02 | | | |  |
|  | Normal | -0.13(-0.18- -0.08) | |  | -0.17(-0.24- -0.09) | | -0.18(-0.24- -0.13) | -0.19(-0.31- -0.08) |  |
|  | Overweight | -0.15(-0.19- -0.10) | |  | -0.25(-0.32- -0.19) | | -0.16(-0.21- -0.10) | -0.20(-0.29- -0.12) |  |
|  | Obesity | -0.09(-0.15- -0.04) | |  | -0.17(-0.26- -0.07) | | -0.26(-0.33- -0.20) | -0.11(-0.22-0.004) |  |
| Data are presented as β (95% confidence interval) for the association between early‑life tobacco exposure and SF coupling, stratified by BMI category.  Abbreviations: BMI, body mass index; CI, confidence interval; L, left; R, right; SF, structure‑function coupling.  * All *p* values were two-sided. *p* _interaction_ were FDR-corrected, with significance defined as *p* < .05. | | | | | | | | |  |

| **eTable 30. Destrieux and subcortical region abbreviations and full names used in the structure-function (SF) coupling analyses** | |
| --- | --- |
| **Abbreviation** | **Full name** |
| L_G_and_S_frontomargin | Left frontomarginalis Gyrus and Sulcus |
| L_G_and_S_occipital_inf | Left Inferior Occipital Gyrus and Sulcus |
| L_G_and_S_paracentral | Left Paracentral Gyrus and Sulcus |
| L_G_and_S_subcentral | Left Subcentral Gyrus and Sulcus |
| L_G_and_S_transv_frontopol | Left Transverse Frontopolar Gyrus and Sulcus |
| L_G_and_S_cingul_Ant | Left Anterior Cingulate Gyrus and Sulcus |
| L_G_and_S_cingul_Mid_Ant | Left Middle Anterior Cingulate Gyrus and Sulcus |
| L_G_and_S_cingul_Mid_Post | Left Middle Posterior Cingulate Gyrus and Sulcus |
| L_G_cingul_Post_dorsal | Left Dorsal Posterior Cingulate Gyrus |
| L_G_cingul_Post_ventral | Left Ventral Posterior Cingulate Gyrus |
| L_G_cuneus | Left Cuneus Gyrus |
| L_G_front_inf_Opercular | Left Inferior Frontal Gyrus (Pars Opercularis) |
| L_G_front_inf_Orbital | Left Inferior Frontal Gyrus (Pars Orbitalis) |
| L_G_front_inf_Triangul | Left Inferior Frontal Gyrus (Pars Triangularis) |
| L_G_front_middle | Left Middle Frontal Gyrus |
| L_G_front_sup | Left Superior Frontal Gyrus |
| L_G_Ins_lg_and_S_cent_ins | Left Long Insular Gyrus and Central Insular Sulcus |
| L_G_insular_short | Left Short Insular Gyrus |
| L_G_occipital_middle | Left Middle Occipital Gyrus |
| L_G_occipital_sup | Left Superior Occipital Gyrus |
| L_G_oc_temp_lat_fusifor | Left Lateral Occipitotemporal Fusiform Gyrus |
| L_G_oc_temp_med_Lingual | Left Medial Occipitotemporal Lingual Gyrus |
| L_G_oc_temp_med_Parahip | Left Medial Occipitotemporal Parahippocampal Gyrus |
| L_G_orbital | Left Orbital Gyrus |
| L_G_pariet_inf_Angular | Left Inferior Parietal Angular Gyrus |
| L_G_pariet_inf_Supramar | Left Inferior Parietal Supramarginal Gyrus |
| L_G_parietal_sup | Left Superior Parietal Gyrus |
| L_G_postcentral | Left Postcentral Gyrus |
| L_G_precentral | Left Precentral Gyrus |
| L_G_precuneus | Left Precuneus Gyrus |
| L_G_rectus | Left Rectus Gyrus (Straight Gyrus) |
| L_G_subcallosal | Left Subcallosal Gyrus |
| L_G_temp_sup_G_T_transv | Left Superior Temporal Transverse Gyrus (Heschl's Gyrus) |
| L_G_temp_sup_Lateral | Left Lateral Superior Temporal Gyrus |
| L_G_temp_sup_Plan_polar | Left Planum Polare of Superior Temporal Gyrus |
| L_G_temp_sup_Plan_tempo | Left Planum Temporale of Superior Temporal Gyrus |
| L_G_temporal_inf | Left Inferior Temporal Gyrus |
| L_G_temporal_middle | Left Middle Temporal Gyrus |
| L_Lat_Fis_ant_Horizont | Left Anterior Horizontal Ramus of Lateral Fissure |
| L_Lat_Fis_ant_Vertical | Left Anterior Vertical Ramus of Lateral Fissure |
| L_Lat_Fis_post | Left Posterior Ramus of Lateral Fissure |
| L_Pole_occipital_ | Left Occipital Pole |
| L_Pole_temporal | Left Temporal Pole |
| L_S_calcarine | Left Calcarine Sulcus |
| L_S_central | Left Central Sulcus (Rolandic Fissure) |
| L_S_cingul_Marginalis | Left Marginal Cingulate Sulcus |
| L_S_circular_insula_ant | Left Anterior Circular Sulcus of Insula |
| L_S_circular_insula_inf | Left Inferior Circular Sulcus of Insula |
| L_S_circular_insula_sup | Left Superior Circular Sulcus of Insula |
| L_S_collat_transv_ant | Left Anterior Collateral Transverse Sulcus |
| L_S_collat_transv_post | Left Posterior Collateral Transverse Sulcus |
| L_S_front_inf | Left Inferior Frontal Sulcus |
| L_S_front_middle | Left Middle Frontal Sulcus |
| L_S_front_sup | Left Superior Frontal Sulcus |
| L_S_interm_prim_Jensen | Left Intermediate Primordial Sulcus of Jensen |
| L_S_intrapariet_and_P_trans | Left Intraparietal and Parieto-occipital Transition Sulcus |
| L_S_oc_middle_and_Lunatus | Left Middle Occipital and Lunate Sulcus |
| L_S_oc_sup_and_transversal | Left Superior Occipital and Transverse Sulcus |
| L_S_occipital_ant | Left Anterior Occipital Sulcus |
| L_S_oc_temp_lat | Left Lateral Occipitotemporal Sulcus |
| L_S_oc_temp_med_and_Lingual | Left Medial Occipitotemporal and Lingual Sulcus |
| L_S_orbital_lateral | Left Lateral Orbital Sulcus |
| L_S_orbital_med_olfact | Left Medial Orbital (Olfactory) Sulcus |
| L_S_orbital_H_Shaped | Left H-shaped Orbital Sulcus |
| L_S_parieto_occipital | Left Parieto-occipital Sulcus |
| L_S_pericallosal | Left Pericallosal Sulcus |
| L_S_postcentral | Left Postcentral Sulcus |
| L_S_precentral_inf_part | Left Inferior Part of Precentral Sulcus |
| L_S_precentral_sup_part | Left Superior Part of Precentral Sulcus |
| L_S_suborbital | Left Suborbital Sulcus |
| L_S_subparietal | Left Subparietal Sulcus |
| L_S_temporal_inf | Left Inferior Temporal Sulcus |
| L_S_temporal_sup | Left Superior Temporal Sulcus |
| L_S_temporal_transverse | Left Transverse Temporal Sulcus |
| R_G_and_S_frontomargin | Right Gyrus and Sulcus frontomarginalis |
| R_G_and_S_occipital_inf | Right Inferior Occipital Gyrus and Sulcus |
| R_G_and_S_paracentral | Right Paracentral Gyrus and Sulcus |
| R_G_and_S_subcentral | Right Subcentral Gyrus and Sulcus |
| R_G_and_S_transv_frontopol | Right Transverse Frontopolar Gyrus and Sulcus |
| R_G_and_S_cingul_Ant | Right Anterior Cingulate Gyrus and Sulcus |
| R_G_and_S_cingul_Mid_Ant | Right Middle Anterior Cingulate Gyrus and Sulcus |
| R_G_and_S_cingul_Mid_Post | Right Middle Posterior Cingulate Gyrus and Sulcus |
| R_G_cingul_Post_dorsal | Right Dorsal Posterior Cingulate Gyrus |
| R_G_cingul_Post_ventral | Right Ventral Posterior Cingulate Gyrus |
| R_G_cuneus | Right Cuneus Gyrus |
| R_G_front_inf_Opercular | Right Inferior Frontal Gyrus (Pars Opercularis) |
| R_G_front_inf_Orbital | Right Inferior Frontal Gyrus (Pars Orbitalis) |
| R_G_front_inf_Triangul | Right Inferior Frontal Gyrus (Pars Triangularis) |
| R_G_front_middle | Right Middle Frontal Gyrus |
| R_G_front_sup | Right Superior Frontal Gyrus |
| R_G_Ins_lg_and_S_cent_ins | Right Long Insular Gyrus and Central Insular Sulcus |
| R_G_insular_short | Right Short Insular Gyrus |
| R_G_occipital_middle | Right Middle Occipital Gyrus |
| R_G_occipital_sup | Right Superior Occipital Gyrus |
| R_G_oc_temp_lat_fusifor | Right Lateral Occipitotemporal Fusiform Gyrus |
| R_G_oc_temp_med_Lingual | Right Medial Occipitotemporal Lingual Gyrus |
| R_G_oc_temp_med_Parahip | Right Medial Occipitotemporal Parahippocampal Gyrus |
| R_G_orbital | Right Orbital Gyrus |
| R_G_pariet_inf_Angular | Right Inferior Parietal Angular Gyrus |
| R_G_pariet_inf_Supramar | Right Inferior Parietal Supramarginal Gyrus |
| R_G_parietal_sup | Right Superior Parietal Gyrus |
| R_G_postcentral | Right Postcentral Gyrus |
| R_G_precentral | Right Precentral Gyrus |
| R_G_precuneus | Right Precuneus Gyrus |
| R_G_rectus | Right Rectus Gyrus (Straight Gyrus) |
| R_G_subcallosal | Right Subcallosal Gyrus |
| R_G_temp_sup_G_T_transv | Right Superior Temporal Transverse Gyrus (Heschl's Gyrus) |
| R_G_temp_sup_Lateral | Right Lateral Superior Temporal Gyrus |
| R_G_temp_sup_Plan_polar | Right Planum Polare of Superior Temporal Gyrus |
| R_G_temp_sup_Plan_tempo | Right Planum Temporale of Superior Temporal Gyrus |
| R_G_temporal_inf | Right Inferior Temporal Gyrus |
| R_G_temporal_middle | Right Middle Temporal Gyrus |
| R_Lat_Fis_ant_Horizont | Right Anterior Horizontal Ramus of Lateral Fissure |
| R_Lat_Fis_ant_Vertical | Right Anterior Vertical Ramus of Lateral Fissure |
| R_Lat_Fis_post | Right Posterior Ramus of Lateral Fissure |
| R_Pole_occipital | Right Occipital Pole |
| R_Pole_temporal | Right Temporal Pole |
| R_S_calcarine | Right Calcarine Sulcus |
| R_S_central | Right Central Sulcus (Rolandic Fissure) |
| R_S_cingul_Marginalis | Right Marginal Cingulate Sulcus |
| R_S_circular_insula_ant | Right Anterior Circular Sulcus of Insula |
| R_S_circular_insula_inf | Right Inferior Circular Sulcus of Insula |
| R_S_circular_insula_sup | Right Superior Circular Sulcus of Insula |
| R_S_collat_transv_ant | Right Anterior Collateral Transverse Sulcus |
| R_S_collat_transv_post | Right Posterior Collateral Transverse Sulcus |
| R_S_front_inf | Right Inferior Frontal Sulcus |
| R_S_front_middle | Right Middle Frontal Sulcus |
| R_S_front_sup | Right Superior Frontal Sulcus |
| R_S_interm_prim_Jensen | Right Intermediate Primordial Sulcus of Jensen |
| R_S_intrapariet_and_P_trans | Right Intraparietal and Parieto-occipital Transition Sulcus |
| R_S_oc_middle_and_Lunatus | Right Middle Occipital and Lunate Sulcus |
| R_S_oc_sup_and_transversal | Right Superior Occipital and Transverse Sulcus |
| R_S_occipital_ant | Right Anterior Occipital Sulcus |
| R_S_oc_temp_lat | Right Lateral Occipitotemporal Sulcus |
| R_S_oc_temp_med_and_Lingual | Right Medial Occipitotemporal and Lingual Sulcus |
| R_S_orbital_lateral | Right Lateral Orbital Sulcus |
| R_S_orbital_med_olfact | Right Medial Orbital (Olfactory) Sulcus |
| R_S_orbital_H_Shaped | Right H-shaped Orbital Sulcus |
| R_S_parieto_occipital | Right Parieto-occipital Sulcus |
| R_S_pericallosal | Right Pericallosal Sulcus |
| R_S_postcentral | Right Postcentral Sulcus |
| R_S_precentral_inf_part | Right Inferior Part of Precentral Sulcus |
| R_S_precentral_sup_part | Right Superior Part of Precentral Sulcus |
| R_S_suborbital | Right Suborbital Sulcus |
| R_S_subparietal | Right Subparietal Sulcus |
| R_S_temporal_inf | Right Inferior Temporal Sulcus |
| R_S_temporal_sup | Right Superior Temporal Sulcus |
| R_S_temporal_transverse | Right Transverse Temporal Sulcus |
| R_hippocampus | Right Hippocampus |
| R_amygdala | Right Amygdala |
| R_posterior Thalamus | Right Posterior Thalamus |
| R_anterior Thalamus | Right Anterior Thalamus |
| R_nucleus accumbens | Right Nucleus Accumbens |
| R_globus pallidus | Right Globus Pallidus |
| R_putamen | Right Putamen |
| R_caudate nucleus | Right Caudate Nucleus |
| L_hippocampus | Left Hippocampus |
| L_amygdala | Left Amygdala |
| L_posterior thalamus | Left Posterior Thalamus |
| L_anterior thalamus | Left Anterior Thalamus |
| L_nucleus accumbens | Left Nucleus Accumbens |
| L_globus pallidus | Left Globus Pallidus |
| L_putamen | Left Putamen |
| L_caudate nucleus | Left Caudate Nucleus |
| Region labels follow the naming conventions of the Destrieux cortical atlas and the subcortical parcellation used in the present study. L indicates left; R indicates right; G, gyrus; and S, sulcus. | |

**eFigure 1. Associations between inflammatory markers and the incidence of depression and anxiety**

**(A)** Exposure–response curves between inflammatory markers and depression risk revealed significant non-linear associations for all nine markers. **(B)** Exposure–response curves for inflammatory markers and anxiety risk revealed significant non-linear associations for eight markers (excluding neutrophils).

**eFigure 2. Associations between brain regional SF coupling and the incidence of depression and anxiety**

**(A)** Among the 17 brain regions affected by in utero tobacco exposure, four regions showed significant associations with depression incidence: the left mid-posterior cingulate gyrus and sulcus, left superior temporal sulcus, right inferior occipital gyrus and sulcus, and right posterior lateral fissure.**(B)** Among the 17 regions affected by in utero tobacco exposure, only the right inferior part of the precentral sulcus was significantly associated with anxiety incidence. **(C)** Among the 50 brain regions differing by age of smoking initiation, six regions were significantly associated with depression incidence: the left superior occipital sulcus and transversal sulcus, right inferior occipital gyrus and sulcus, right anterior cingulate gyrus and sulcus, right superior frontal sulcus, right posterior thalamus, and right anterior thalamus. **(D**) Among the 50 regions differing by age of smoking initiation, five regions were significantly associated with anxiety incidence: the left central sulcus, right middle frontal gyrus, right superior frontal sulcus, right inferior part of the precentral sulcus, and right superior part of the precentral sulcus.

**eFigure 3. Brain regional SF coupling associated with early-life tobacco exposure was significantly linked to inflammatory markers.**

**(A)** Following FDR correction (p < 0.05), six inflammatory markers—C-reactive protein (CRP), leukocyte count, lymphocyte percentage, lymphocyte count, monocyte count, and neutrophil count—showed significant associations with SF coupling in brain regions previously identified as affected by in utero tobacco exposure. **(B)** Following FDR correction (p < 0.05), seven inflammatory markers—CRP, leukocyte count, lymphocyte count, lymphocyte percentage, monocyte count, neutrophil percentage, and platelet count—were significantly associated with SF coupling in brain regions previously identified as affected by the age of smoking initiation. L, left; R, right. (C) Age of smoking initiation influences SF coupling in specific brain regions through shared inflammatory markers. The y axis represents the proportion of total variance due to mediation (%). Blue, green, and orange dots indicate the mediation proportions for childhood, adolescence, and adulthood smoking initiation, respectively. Each panel represents a specific inflammatory marker (CRP, leukocyte, lymphocyte, and monocyte), with corresponding brain regions displayed above the bars.

**eFigure 4. Associations between age of smoking initiation and incident depression stratified by covariates**

**(A) Among all covariates, depression interacted significantly with several covariates including BMI, ethnicity, alcohol use, deprivation and age on incident depression. The dashed vertical line indicates threshold of significance after correcting for multiple testing (−log(0.05/10) = 2.30). (B) Among all covariates, only deprivation, alcohol use and ethnicity showed a significant interaction with age of smoking initiation on incident anxiety. The dashed vertical line indicates threshold of significance after correcting for multiple testing (−log(0.05/10) = 2.30).**

eReferences

1	Miller, K. L., Alfaro-Almagro, F., Bangerter, N. K., Thomas, D. L., Yacoub, E., Xu, J., et al. Multimodal population brain imaging in the UK Biobank prospective epidemiological study. *Nature neuroscience* **19**, 1523-1536 (2016).

2	Alfaro-Almagro, F., Jenkinson, M., Bangerter, N. K., Andreassen, O. A., Griffanti, L., Douaud, G., et al*.* Image processing and Quality Control for the first 10,000 brain imaging datasets from UK Biobank. *Neuroimage* **166**, 400-424 (2018).
